# Five undervalued edible species inherent to autumn-winter season: nutritional composition, bioactive constituents and volatiles profile

**DOI:** 10.7717/peerj.12488

**Published:** 2021-11-23

**Authors:** Tamara Fukalova Fukalova, María Dolores García Martínez, María Dolores Raigón

**Affiliations:** 1 Facultad de Ciencias Químicas, Universidad Central del Ecuador, Quito, Pichincha, Ecuador; 2 Instituto de Conservación y Mejora de la Agrobiodiversidad Valenciana, Universitat Politècnica de València, Valencia, Spain

**Keywords:** Antioxidants, Chlorophylls, Nutritional quality, Undervalued species, Volatile profiles

## Abstract

**Background:**

Wild edible herbs have historically been used as local nutritional and medicinal sources. These plants grow spontaneously, depending on the season. They adapt well to different edaphoclimatic conditions, generating a diversity constituent beneficial to health. They impart compounds needed in the human diet in regard to macro and micronutrients. When consumed raw, they keep their properties intact and provide health benefits. Five undervalued edible plants: *Stellaria media* (L.) Vill, *Tropaeolum majus* L*., Sonchus oleraceus* L*., Chenopodium album* L. and *Diplotaxis erucoides* (L.) DC are characteristic of the autumn-winter season in the Valencian coastal region and could have new sustainable agro-ecological potential for the local commercial sector. However, little information is available from the nutritional quality and bioactive composition viewpoint for these species. Concurrently, the volatiles compounds profiles describing the characteristic flavors are unknown.

**Methods:**

Nutritional characteristics, bioactive compounds, and other chemical components of the fresh leaves were analyzed. In addition, the volatiles composite profile was performed. The analyzed species come from the soil reservoir; their wild growth is adjusted to the autumn season. The proximate analysis was carried out by Association of Official Analytical Chemists methods. Total antioxidants were measured as 2.2-diphenyl-1-picrylhydrzyl hydrate (DPPH) and total polyphenols content *via* the Folin-Ciocalteu procedure. Volatiles profile was determined by gas chromatography-mass spectrometry. The vegetative part analyzed was the tender leaves with edible potential.

**Results:**

A high variability has been obtained in the composition of the species studied. The proximate analysis found a considerable content of fiber (1.22–5.4 g·100 g^−1^), potassium (157.7–1,250.6 mg·100 g^−1^), iron (0.6–2.0 mg·100 g^−1^), and a low caloric value (16.1–43.02 kcal·mg·100 g^−1^). In bioactive compounds analysis, a high level of antioxidants was highlighted (1,604.3–4,874.6 μmol·100 g^−1^), followed by chlorophylls. Volatiles profile revealed that the species were rich in benzenoids (33.8–89.9%) as the majority family. The pyrazines class was characteristic only in *D. erucoides* L.

**Discussion:**

Fresh edible leaves of the undervalued plants show considerable nutritional potential and a high bioactive components level, which highlight the antioxidant capacity. Leaves of *C. album* L. stand out due to their higher concentration of nutritional compounds, while *D. erucoides* L. is noted for its higher antioxidant capacity. Aromatic descriptor of pyrazines detected in the leaves of *D. erucoides* L. is associated with the slightly spicy flavors that characterize this species. Results suggest that studied species could be of great relevance in introducing these five edible herbs as a source of new grown material, postulating them as healthy food ingredients with attractive flavors for the gourmet cuisine industry.

## Introduction

Wild resources are an essential part of biocultural heritage for all cultures, which have used them for centuries (Rotherham, 2015; Tanús et al., 2019). Within these resources, the plants are a vital support in extreme environmental conditions and threatened habitats, as they possess many health-promoting values (Murthy & Paek, 2020). It has been widely observed (De Cortes Sánchez-Mata & Tardío, 2016) that the Mediterranean macrobioclimate and its ecosystems show remarkably high diversity in the heterogeneity of their plants. This biodiversity includes the wild plants that are used for nutritional and therapeutical purposes. Many wild edible plants are characteristic of their seasonality and can only be consumed in certain seasons of the year. The local people appreciate these plants for their organoleptic properties and a large number of edible wild plants is still included in traditional diets. Nevertheless, the use of wild vegetables has often been relegated to local perception and has been globally undervalued, despite its valuable contribution of minerals and vitamins in certain seasons.

Climate change is expected to have a negative impact on the four pillars of food security: availability, access, utilization, and stability, which will affect the food system (Mbow et al., 2019). Food systems such as food biodiversity also contribute to human and animal diets. A Global Burden Disease study report carried out in 204 countries concluded that there is an urgency to carry out a coordinated global effort to improve the quality of the human diet (Vos et al., 2020). According to this report, undervalued wild plants could be considered to have potential to improve the quality of the human diet for their micronutrient content, especially vitamins, minerals and other phytochemical compounds with antioxidant properties. Moreover, the epidemiological evidence indicates a correlation between the intake of food rich in antioxidants and the reduction of certain non-communicable diseases (Wagner & Brath, 2012; Iriti, Varoni & Vitalini, 2020; Ros & Gonzalez, 2020).

The continental Spanish flora contains 6,152 species, 53% of them being European flora and 15% of plants being endemic to this area (Aedo, Medina & Fenandez-Albert, 2013). This abundance of herbs explains the extensive consumption of wild edible plants in food traditions and why the Mediterranean diet is declared Intangible Heritage of Humanity by UNESCO (González-Turmo & Medina, 2012). Seasonality is very strong under Mediterranean climate conditions. For this reason, certain wild plants are collected and consumed only at specific times of the year (De Cortes Sánchez-Mata & Tardío, 2016). The leaves of some species are predominantly eaten either raw or cooked. Eating the fresh raw materials fresh is probably the best way of getting all the benefits attributed to these vegetables (Bennett et al., 2006), because the nutrients and bioactive compounds present in the plants are fully preserved when consumed fresh. In addition, the leaves are characterized by individual tastes, depending on the species, genetic diversity, and environment.

Another important aspect provided by the undervalued species of edible leaf is the taste and smell of gastronomic preparations. This makes edible leaf a good material for culinary innovation (García-Herrera et al., 2020). In addition, research on functional ingredients such as vitamins, antioxidants, fatty acids, fiber, and other supposedly therapeutic substances is helping to the development of the functional food market, that becoming the star up of nutrition (Rodríguez, Perea & Anta, 2015).

Nowadays, in most cases, ethnobotanical studies reveal either a dramatic or a gradual loss of traditional knowledge and practices. Only a few species are still widely collected and consumed by older people, but their ethnobotanical knowledge is not being absorbed by the younger generation. This situation leads to the underutilization of many plants, due to the discontinuity of expertise.

According to their high cultural relevance, as shown in the previous ethnobotanical review (Chidrawar et al., 2011; Morales et al., 2014; Poonia & Upadhayay, 2015; Avato & Argentieri, 2015; Tardío et al., 2016; Jakubczyk et al., 2018; Panfili et al., 2020), as well as the growing popularity of vegetable salads in the traditional diet of the Valencian coast, five different undervalued wild species were chosen: *Stellaria media* (L.) Vill, *Tropaeolum majus* L., *Sonchus oleraceus* L., *Chenopodium album* L., *Diplotaxis erucoides* (L.) DC. The five species studied in this research have in common the temporal coexistence of germinating in the early autumn and reaching optimal vegetative development in the autumn-winter period, on the Valencian coast.

Taking into consideration all the points mentioned above, the aim was to analyze the chemical composition, including proximate composition traits (moisture, titrable acidity and nutrients), bioactive compounds (chlorophylls, total phenolics and antioxidant activity), and minerals. An additional goal was to make the volatiles profile as odor-active compounds of fresh leaves. To the authors’ knowledge, the volatiles profile research is the first study of the selected species. Supplementary to this, it was proposed to obtain information about the nutritional potential of these plants in order to support their consumption, with the aim of promoting knowledge about the benefits of these undervalued wild edible plants and thus enhancing their uses as suitable raw materials in healthy and sustainable diets, and also as food recovery for application in gourmet gastronomy.

## Materials & methods

Field experiments were approved by the Free Research of the Universitat Politécnica de Valencia (ID Tesis 1316).

### Plant material and sample preparation

The five species studied have been chosen for having an abundant presence in Mediterranean soils from the beginning of autumn, expanding their presence throughout the winter. In all cases, these are undervalued species for current consumption. The species became present in popular gastronomy years ago, but at the present time, they are not part of the common intake of the area. Due to their organoleptic properties, they could be of great interest in food production and consumption. The five species appear spontaneously, after the first autumn rains (late September) and with the drop-in soil temperature. The life cycle varies, depending on the genetics and the edaphoclimatic conditions.

*Stellaria media* (L.) Vill is native to Europe, but has been spread by human activities throughout the world, and is now one of the most widespread weeds in the world. Reproduction is predominantly by seeds accumulated in soil. *Tropaeolum majus* L. is an invasive species in many warm regions. It reproduces easily through seeds, but in Mediterranean conditions, deep fleshy roots remain in the ground, leading to spontaneous budding in late October.

*Sonchus oleraceus* L. is an annual plant and a predominantly winter-active weed species (Peerzada, O’Donnell & Adkins, 2019); it is considered as weed lettuce and its reproduction is predominantly by seeds accumulated in soil. *Chenopodium album* L. is an annual herb that grows in all types of soils rich in nitrogen, and is widely spread across the globe, reproduced solely by seeds. There is archaeological evidence to suggest it was cultivated as a pseudo-cereal in Europe in prehistory (Stokes & Rowley-Conwy, 2002). *Diplotaxis erucoides* (L.) DC, is a Mediterranean winter annual weed, which has been identified by analyzing cohorts that emerge in autumn (Sans & Masalles, 1994).

Brief additional descriptions of each species, such as their family, plant name, vernacular names, traditional and medicinal uses are contained in Table 1.

**Table 1:** Description of the wild plant species collected and their uses.

Family	Plant name	Vernacular names	Traditional uses	Medicinal uses	Reference
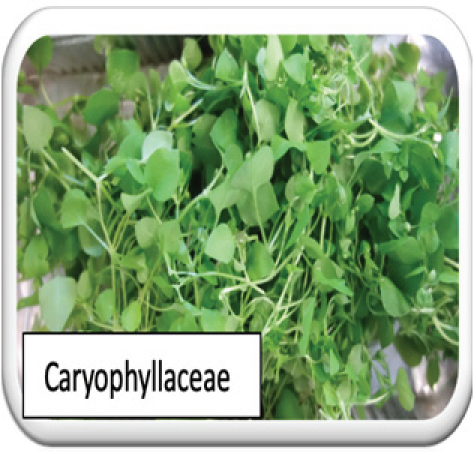	*Stellaria media* (L.) Vill	Common chickweed, chickenwort, craches, maruns, pamplina	Fresh salad, infusion, soup, spice, stews	Expectorant, mucolytic, diuretic, healing, emollient	(Alvarez, 2019; Soutullo et al., 2015)
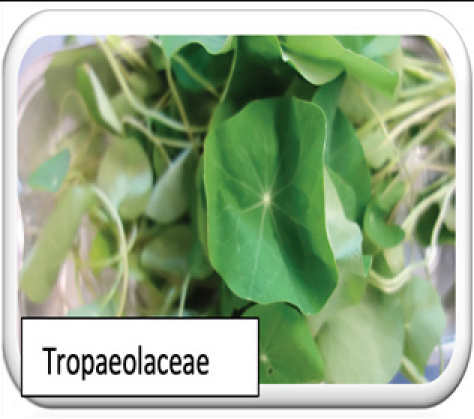	*Tropaeolum majus* L.	Indian cress, climbing nasturtium, monk crees, empress of india	Leaves us salad, fruits us pickled, flowers us desserts and drinks	Aperitif, anti-inflammatory, diuretic, circulation	(Brondani et al., 2016; Junior et al., 2011)
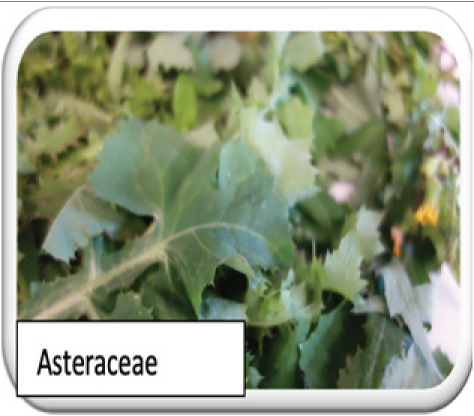	*Sonchus oleraceus* L.	Smooth sowthistle, common sowthistle, milky tasel, swinies	Fresh salad, soup, fried, stewed, infusion, decoction	Laxative, diuretic, digestive, astringent, hypoglycemic	(Chen, Teng & Cao, 2019; Vilela et al., 2010)
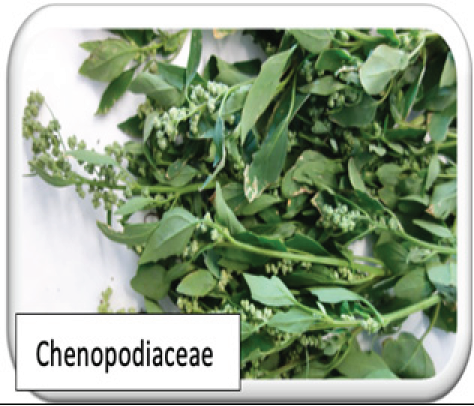	*Chenopodium album* L.	Fat hen baconweed, pigweed, wild spinach, white goosefoot	Vegetable, infusion, seeds to obtain semolina, condiment	Laxative, antiparasitic, antifungal, sedative	(Nengroo & Rauf, 2021; Del Vitto, Petenatti & Petenatti, 1998)
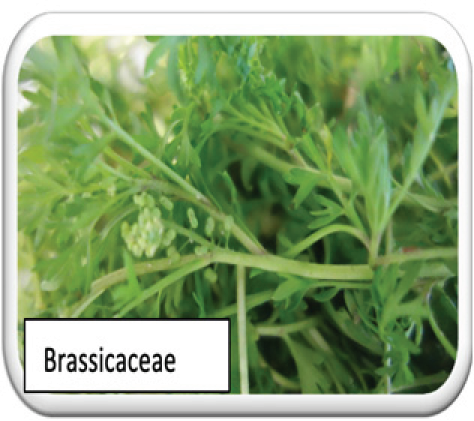	*Diplotaxis erucoides* (L.) DC	White rocket, white rabaniza, mediterranean wasabi, fine caterpillar	Fresh salad, dressing, soup, decoction	Stimulant, diuretic, expectorant, antibacterial	(Bell & Wagstaff, 2019; Ballesta & Negre, 2018)

The random choice of the fresh samples in their natural habitat was carried out in conjunction with ecological cooperative members in rural environments. The area is located within latitudes N 39°45′13″ and longitudes W 0°12′21″, with ecological characterization code 81 (Montesinos, Otto & Fernández Palacios, 2009). Approximately 1 kg of the aerial parts of each species was collected.

The plant samples were manually cleaned by removing soil particles and damaged parts before performing the analysis. Only whole leaves were used, while non-edible portions were eliminated. Parts of the fresh leaves were used for extractions and analytical quantifications of total antioxidants (methanolic extract); polyphenols, nitrates, pH and total acidity (aqueous extract), and chlorophylls (acetonic extract). The rest of the aerial parts were air-dried in ovens (J.P. Selecta, 2000787 model) at 70 °C with low humidity conditions. Dried samples were powdered with a grinder (Retsch KG-5657 Haan) and stored at 4 °C until subsequent analysis (for nutritional components and mineral elements).

### Standards and reagents

All chemicals and solvents were analytically graded. The methanol (80% v/v) and acetone (80% v/v) solutions were prepared from solvents. Sodium carbonate, citric acid, boric acid; sulfuric acid, hydrochloric acid, phosphoric acid, lanthanum (III) chloride and sodium hydroxide (Scharlau). Trolox (6-hydroxy-2,5,7,8-tetramethylchroman-2-carboxylic acid), 2,2′-azobis-2-methyl-propanimidamide, 1,1-diphenyl-2-picrylhydrazyl radical (DPPH), Folin-Ciocalteu reagent (FCR), iron (III) chloride hexahydrate, and gallic acid were purchased from Sigma-Aldrich Co. Water was treated in Water Still Aquatron A4000.

### Nutritional composition

Prior to the sample’s analysis, all analytical methods were optimized and fine-tuned for the specific analysis of this type of matrix. All determinations were performed in triplicate.

#### Proximate analysis

Analyzes were performed following the methods recommended by the Association of Official Analytical Chemists (AOAC) to determine the moisture content (AOAC 984.25), crude protein (AOAC 984.13), fat (AOAC 983.23), crude fiber (AOAC 991.43) and ash (AOAC 923.03). The total carbohydrate content was calculated by difference. The results were expressed in g·100 g^−1^ fresh weight (fw).

#### Mineral analysis

The samples were digested in a Carbolite CWF 1100 muffle at 550 °C, with reference to the AOAC 985.35 method. The calibration curves were established using working standards for each element. Minerals were analyzed by atomic absorption spectroscopy (EAA), using the thermo elemental AA series Spectrometer, software v.11.03 and hollow cathode lamps for each element, except phosphorus, which was analyzed by colorimetry (AOAC, 2005).

### Bioactive constituents

Methanolic extract was obtained by mixing 0.8 g of fresh leaves and tender stems in 5 mL methanol solution (80% v/v); it was stirred for 1 h at room temperature, using an orbital shaker SO1 (Stuart Scientific, Chelmsford Essex, UK). Aqueous extract was obtained by grinding the fresh aerial parts of each plant with water in a ratio of 2:1 (solvent: plant). Extracts were used immediately in respective determinations. Tree replicates were performed for each analysis.

#### Total antioxidants

To measure the extracts effect on the DPPH radical, the optimized method of Brand-Williams, Cuvelier & Berset (1995) was estimated. The DPPH solution (25 ppm in methanol 80% v/v) was prepared, and 3.9 mL of this solution was mixed with 0.1 mL methanolic extract (each sample). Absorbance was measured at 515 nm after 45 min of incubation with DPPH solution in the dark by a spectrophotometer (Schott UV line 9400). The antioxidant Trolox was used as standard, and the results were expressed as micromoles of Trolox equivalents in each 100 g of fresh weight (µmol TE·100 g^−1^ fw).

#### Total polyphenols

Total phenolics were determined according to the Folin-Ciocalteu procedure, with some modifications. An aliquot of aqueous extract (50 µL) was mixed with 500 µL Folin-Ciocalteu Reagent (previously diluted with water 1:10 v/v) and 500 µL Na_2_CO_3_ solution of 6% (w/v). The cuvettes were mixed for 10 s and allowed to stand for 1 h at room temperature for color development. Absorbance was measured at 750 nm (Jenway 6715/UV-Vis spectrophotometer). Gallic acid was used to calculate the standard curve (25 ppm to 400 ppm) and the results were expressed as mg of gallic acid equivalents in each 100 g of fresh weight (mg GAE·100 g^−1^ fw).

#### Chlorophylls a, b and total

All chlorophylls were determined using an adapted method proposed by Hansmann (1973). The crushed aerial parts of each plant were suspended in acetone extraction solution (80% v/v), then they were stirred and filtered to avoid turbidity, and the volume was completed with the same extraction solution. Absorbance was measured at 645, 653 and 663 nm (Schott UV line 9400) immediately. The wavelengths corresponded to chlorophylls a, b and total respectively. The results were expressed as micrograms of grams of fresh weight (μg·g^−1^ fw).

### Other chemical components

Fresh plant subsamples were mixed with water in a ratio of 1:2 (w/v) at a temperature below 30 °C by mechanical grinder. The nitrates and pH direct analysis were performed immediately with respective electrodes by pH & Ion-Meter GLP 22+ (Crison Instruments, Barcelona, Spain). The results for nitrates were expressed as milligram per each kg of fresh wight (mg NO_3_^−^·kg^−1^ fw). Content in total acidity was determined potentiometrically, with titration of the NaOH 0.05 N solution. The results were expressed as a citric acid percentage.

### Analysis of volatiles profile

Preparation of samples (5 g) and extraction of volatile compounds was performed by the HS-SPME technique according to Moreno et al. (2012). Volatile aroma compounds of the leaves were analyzed by gas chromatography-mass spectrometry (GC-MS), using a 6890N Network GC System with autosampler coupled to a 5973 Inert Mass Selective Detector (Agilent Technologies, Santa Clara, CA, USA). Analytical conditions: the stationary phase was HP-5MS J&W silica capillary column (5% phenyl-95% methylpolysiloxane); the carrier gas was helium at a constant flow of 1 mL min^−1^; the transfer line was maintained at 220 °C; the electron impact (EI) mode with 70 eV ionization energy (source temperature 225 °C) was used for detection by the mass spectrometer, and acquisition was performed in scanning mode (mass range m/z 35–350 amu). Volatile extractions were run in triplicate.

### Statistical analysis

The individual values of nutritional, mineral, and bioactive analysis were used to obtain the mean value and the standard error for the five species studied. Data were analysed using a one-way analysis of variance (ANOVA), considering the type of specie as a factor. Statistical significance was evaluated using Tukey Honestly Significant Difference (HSD), with a cut-off significance of *p* < 0.05. Principal component analysis (PCA) was applied to improve the visualization of the results. This analysis was carried out by considering two cases: (A) The parameters of the nutrients-minerals-chemical group; and (B) the parameters of the bioactive components group. To perform PCA, linear regressions were operated on the data of covariance matrix in order to select the two highest principal components of each group. All statistical analysis was affected in the Statgraphics Plus software, version 5.1 (Manugistics. Inc., Rockville, MD, USA). Finally, for the analysis of volatiles profiles, the relative abundances of each group of chemical family were calculated against the total identified in each sample, and were expressed as percentages; an illustrative comparison of profiles was performed on Excel sheets.

## Results

The nutritional profile, minerals, and chemical composition of the studied five wild species, expressed as fresh weights (fw), were evaluated and well summarized in Table 2. Proximate composition showed moisture, ash, crude protein, fat, crude fiber and carbohydrate contents. Mineral content was determined in terms of macro minerals (Ca, Mg, K, P, Na) and micro minerals (Fe, Cu, Zn), expressed as mg in each·100 g of fresh leaves. The chemical components, such as NO_3_^−^, pH and total acidity, were also determined, because they have importance in edible plants.

**Table 2:** Mean values with standard deviation and coefficient of variability of nutritional, mineral and chemical composition of five wild species.

		*S.* *media (*L.) Vill		*T.* *majus* L.		*S.* *oleraceus* L.		*C.* *album* L.		*D. erucoides* (L.) DC	
		CV (%)		CV (%)		CV (%)		CV (%)		CV (%)	
**Nutritional value (g 100 g** ^**−1**^ **)**	Humidity	91.64 ± 2.08^a^	2.27	89.59 ± 0.25^a^	0.27	89.21 ± 1.08^a^	1.21	80.19 ± 0.91^b^	1.13	88.27 ± 0.74^a^	0.84
	Ash	2.01 ± 0.47^a^	23.48	1.87 ± 0.04^a^	1.88	2.11 ± 0.23^a^	11.06	3.97 ± 0.24^a^	6.15	2.18 ± 0.19^a^	8.87
	Proteins	0.20 ± 0.04^c^	23.45	1.82 ± 0.10^b^	5.47	1.76 ± 0.08^b^	4.69	2.24 ± 0.05^a^	2.04	2.25 ± 0.05^a^	2.03
	Fat	0.39 ± 0.12^b^	30.64	0.45 ± 0.11^a^	23.35	0.35 ± 0.02^b^	5.36	0.25 ± 0.03^c^	13.72	0.25 ± 0.04^c^	14.16
	Fiber	1.22 ± 0.42^c^	34.19	5.08 ± 0.62^a^	12.13	3.66 ± 0.85^b^	23.13	5.40 ± 0.44^a^	8.19	2.93 ± 0.35^b^	11.78
	Carbohyrate	4.55 ± 1.29^b^	28.38	1.18 ± 0.34^c^	28.88	2.90 ± 0.15^d^	5.34	7.94 ± 0.36^a^	4.58	4.13 ± 0.64^b^	15.43
	Energy value (kcal 100 g^−1^)	22.47 ± 4.31	19.20	16.10 ± 3.35	20.84	21.78 ± 0.29	1.35	43.02 ± 1.00	2.34	27.73 ± 2.57	9.27
**Minerals (mg 100 g** ^**−1**^ **)**	Calcium	71.5 ± 21.5^c^	30.06	119.6 ± 12.8^b^	10.72	119.2 ± 8.5^b^	7.13	313.2 ± 50.5^a^	16.12	60.0 ± 27.2^c^	45.38
	Magnesium	82.6 ± 20.1^c^	24.28	67.4 ± 9.0^c^	13.38	78.7 ± 8.9^c^	11.29	480.6 ± 107.3^a^	22.34	114.1 ± 26.3^b^	23.07
	Potassium	710.1 ± 99.7^b^	14.04	574.7 ± 61.3^c^	10.67	714.9 ± 115.9^b^	16.21	1,250.6 ± 59.6^a^	4.77	157.7 ± 11.6^d^	7.36
	Phosphorus	44.7 ± 12.8^b^	28.54	49.3 ± 3.1^b^	6.25	50.1 ± 6.1^b^	12.18	81.8 ± 6.2^a^	7.59	47.7 ± 6.8^b^	14.32
	Sodium	24.8 ± 8.9^b^	36.12	16.1 ± 0.3^b^	1.76	39.4 ± 8.2^a^	20.79	7.5 ± 0.8^c^	10.57	14.8 ± 1.1^c^	7.47
	Iron	1.3 ± 0.4^b^	33.69	0.6 ± 0.0^c^	5.36	1.5 ± 0.3^a^	19.71	2.0 ± 0.2^a^	11.26	1.2 ± 0.1^b^	12.06
	Copper	0.1 ± 0.01^b^	20.25	0.1 ± 0.0^b^	59.91	0.1 ± 0.03^b^	47.52	0.2 ± 0.09^a^	48.06	0.1 ± 0.0^b^	47.52
	Zinc	0.8 ± 0.2^a^	32.34	0.7 ± 0.1^b^	12.65	0.7 ± 0.1^b^	12.88	0.8 ± 0.1^a^	16.59	0.5 ± 0.1^c^	16.79
**Chemicals**	Nitrates (mg NO_3_^−^·kg^−1^)	75.62 ± 6.00^b^	7.9	56.33 ± 5.21^c^	9.2	92.82 ± 10.3^a^	11.1	31.61 ± 6.29^d^	19.9	17.96 ± 2.86^e^	15.9
	pH	6.12 ± 0.11^b^	1.9	6.00 ± 0.19^b^	3.3	6.21 ± 0.06^b^	1.0	6.54 ± 0.15^a^	2.3	5.72 ± 0.05^c^	0.9
	Acidity total (% cítric acid)	0.15 ± 0.01^b^	8.9	0.17 ± 0.02^b^	10.6	0.12 ± 0.02^b^	17.1	0.12 ± 0.01^c^	6.0	0.29 ± 0.05^a^	15.9

**Note:**

^a–e^Superscript showed that a significant difference exist: humidity, ash, crude protein, crude fiber, carbohydrate, Ca, Mg, K, Zn, NO_3_^−^, acidity total (*p* = 0.000); fat (*p* = 0.028); P, Fe, Cu (*p* = 0.001) and Na, pH (*p* = 0.003).

High moisture values were observed in most vegetables’ species. There are significant differences (*p* = 0.000) between the moisture content of the edible leaves of *C. album* L. (80.18%) compared to the moisture values of the rest of the leaves analyzed. The ash content ranged between 1.87% (*T. majus* L.) and 3.97% (*C. album* L.). The ash parameter presented the highest variability values, being the leaves of *S. media* L.-those that presented high variation coefficients (23.48%) in this parameter-followed by *S. oleraceus* (11.06%). Most species had a low protein content, ranging from 0.20% (*S. media* L.) to 2.25% (*D. erucoides* L.), and a low fat content from 0.25% (*C*. *album* L. and *D. erucoides* L.) to 0.45% (*T. majus* L.). In general, the species were characterized mainly by a high content of fiber, carbohydrates and minerals. For the total fiber content, levels were between 1.22% (*S. media* L.) and 5.40% (*C. album* L.); for carbohydrates they were between 1.18% (*T. majus* L.) and 7.94% (*C. album* L.). All undervalued vegetable species analyzed showed a very low energetic value, less than 30 kcal 100 g^−1^, except for *C. album* L. with 42.03 kcal 100 g^−1^. The lowest caloric value was for *T. majus* L. (16.10 kcal 100 g^−1^ fresh leaves).

The mineral contents in the leaves and tender stems of studied plants, expressed as mg·100 g^−1^ fresh weight, are presented in Table 2. The relative standard deviation of mean values was wide in some cases. The richest source of all macro minerals, except sodium, was the *C. album* L. species, with 313.2 mg 100 g^−1^ (Ca), 480.6 mg 100 g^−1^ (Mg), 1,250.6 mg 100 g^−1^ (K), and 81.8 mg 100 g^−1^ (P). The highest content of sodium was found in *S. oleraceus* L., with 39.4 mg 100 g^−1^. The variations in potassium content were highly significant (*p* = 0.000), with *D. erucoides* L. being the one with the lowest content (157.7 mg 100 g^−1^) and *C. album* L. being the analyzed species with the highest concentration in this macroelement. Magnesium, as the second most important element in the studied leaves, showed high variability among the studied species, with *C. album* L. being the one with the highest concentration (*p* = 0.000). As regards calcium, not all species had a good contribution of this element, as it ranged from 60.0 mg 100 g^−1^ (*D. erucoides* L.) to the content in *C. album* L. (statistically significant differences). Sodium was a characteristic element in *S. oleraceus* L., with its highest value being (*p* = 0.003). Phosphorus had similar concentrations in all species, except for *C. album* L. (*p* = 0.001), where it exceeded by 1.6 times the values found in the other species. The same species was also shown as a rich source of microminerals. The iron content of the edible part of the leaves was significantly higher (*p* = 0.001) for *C. album* L. (2.0 mg 100 g^−1^), followed by *S. oleraceus* L. (1.5 mg 100 g^−1^). These values differed from the remaining analyzed species (ranging from 0.6 to 1.3 mg 100 g^−1^). The richest source of zinc was in *S. media* L. and *C. album* L. (0.8 mg 100 g^−1^).

The results of the determination of chemical content on fresh weight are shown in Table 2. Nitrates were found to have the highest content in *S. oleraceus* L. (92.82 mg NO_3_^−^ kg^−1^) and the least content in *D. erucoides* L. (17.96 mg NO_3_^−^ kg^−1^); significant differences (*p* = 0.000) were found between nitrate values in fresh leaves. In the other species, nitrates were detected at concentrations between 31.61–75.62 mg NO_3_^−^ kg^−1^. Total acidity values were inverse of pH values. There are significant differences (*p* = 0.003) between the values. The most acidic pH was found in *D. erucoides* L., with 5.72, which also had the highest total acidity in the same species, with 0.29% citric acid.

The result of the determination of the bioactive components content in species is shown in Table 3, highlighting the low intraspecific variability of the antioxidant capacity values. The highest TAO concentrations were found in *T. majus* L. (4,874.6 μmol TE·100 g^−1^) and *D. erucoides* L. (4,227.4 μmol TE·100 g^−1^). The TAO value of the three remaining species ranged from 1,537.1 to 1,669.9 μmol TE·100 g^−1^.

**Table 3:** Mean values with standard deviation and coefficient of variability of bioactive compounds of the leaves of five wild species.

		*S.* *media (*L.) Vill		*T. majus* L.		*S. oleraceus* L.		*C. album* L.		*D. erucoides* (L.) DC	
		CV (%)		CV (%)		CV (%)		CV (%)		CV (%)	
**Bioactive components**	TAO (μmol TE·100 g^−1^ fw)	1,604.3 ± 239.8^b^	14.9	4,874.6 ± 132.3^a^	27.1	1,537.1 ± 187.2^b^	12.2	1,669.9 ± 194.4^b^	11.6	4,227.4 ± 74.6^a^	1.8
	TPP (mg GAE·100 g^−1^ fw)	398.8 ± 165.7^a^	41.5	378.1 ± 117.9^a^	31.2	237.6 ± 49.5^a^	20.8	398.8 ± 165.7^a^	41.6	208.6 ± 31.6^a^	15.2
	Chl a (μg·g^−1^ fw)	1.07 ± 0.02^c^	2.0	0.93 ± 0.39^c^	43.0	2.26 ± 0.14^a^	6.4	1.62 ± 0.43^b^	26.5	0.92 ± 0.20^c^	21.2
	Chl b (μg·g^−1^ fw)	0.46 ± 0.04^b^	7.8	0.81 ± 0.29^a^	35.8	0.83 ± 0.05^a^	6.1	0.47 ± 0.11^b^	23.1	0.33 ± 0.05^b^	16.7
	Chl total (μg·g^−1^ fw)	1.53 ± 0.04^b^	2.6	1.74 ± 0.69^b^	39.7	3.09 ± 0.19^a^	6.3	2.08 ± 0.54^a^	25.7	1.25 ± 0.25^b^	19.9

**Note:**

^a–c^Superscript showed that a significant difference exists: TAO (*p* = 0.000); Chl a (*p* = 0.001); Chl b (*p* = 0.004); Chl total (*p* = 0.002).

In the case of TPP, differences were less remarkable in absolute values, and all species presented a similar average value of 303.7 mg GAE·100 g^−1^. The highest chlorophyll a content was accentuated in *S. oleraceus* L. (2.26 μg·g^−1^) and *C. album* L. (1.62 μg·g^−1^). High values of chlorophyll b presented in *T. majus* L. and S. *oleraceus* L. Consequently, high total chlorophyll values were found in the same species, because this one maintains, in general, a similar trend observed for chlorophyll a.

**Figure 1:**
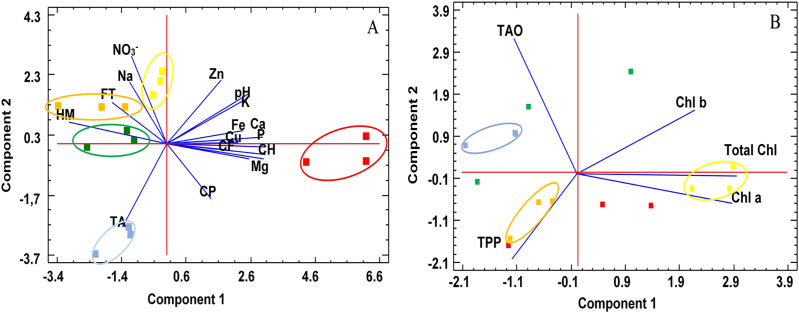
Principal component analysis. (A) Nutrients, minerals and chemicals dispersion diagrams and (B) bioactive dispersion diagram. Orange: *S. media* (L.) Vill L.; Green: *T. majus* L.; Red: *C. album* L.; Blue: *D. erucoides* (L.) DC; Yellow: *S. oleraceus* L.

To reduce the dimensionality of a data set containing many inter-related variables, the PCA method was applied. This allowed global analysis of the results and suggested which attributes characterize the samples. The PCA where the total of the parameters (nutrients-minerals-chemicals) are included showed the first and second components of the PCA, which accounted, respectively, for 54.3% and 20.5% of the total variation. For the first component, all the mineral elements except Na, the nutrients such as protein, fiber and carbohydrates, and the pH value had positive values, whereas fat, moisture, Na, nitrates and total acidity had negative values (Fig. 1A). Regarding the second component, all the parameters had a positive value, except carbohydrates, protein, Mg and total acidity (Fig. 1A). The projections of the combinations on the PCA graph clearly show that the first component mostly separates *C. album* from the rest of the edible plants analyzed. Component 1 was highly correlated with carbohydrates, all minerals (except Na) and pH, and to a lesser degree with crude fiber and protein in the species *C. album* L. Component 2 was highly correlated with total fiber, sodium, and nitrates in the species *S. media* L*., S. oleraceus* L. and *T. majus* L. For *D. erucoides* L., both components showed a negative correlation with total acidity.

The PCA for the bioactive components showed that the first and second components of the PCA accounted, respectively, for 55.2 and 26.1% of the total variation. In this case, the first component, all the chlorophylls (a, b and total) had positive values (Fig. 1B). Regarding the second component, total polyphenols and chlorophyll a had a negative value (Fig. 1B). For *D. erucoides* L. and *T. majus* L., Component 2 was highly correlated with total antioxidants. On the contrary, for *S. oleraceus* L., Component 1 showed a strong relationship with chlorophylls. Finally, both principal components presented positive correlation with total antioxidants in the species *S. media* L.

**Figure 2:**
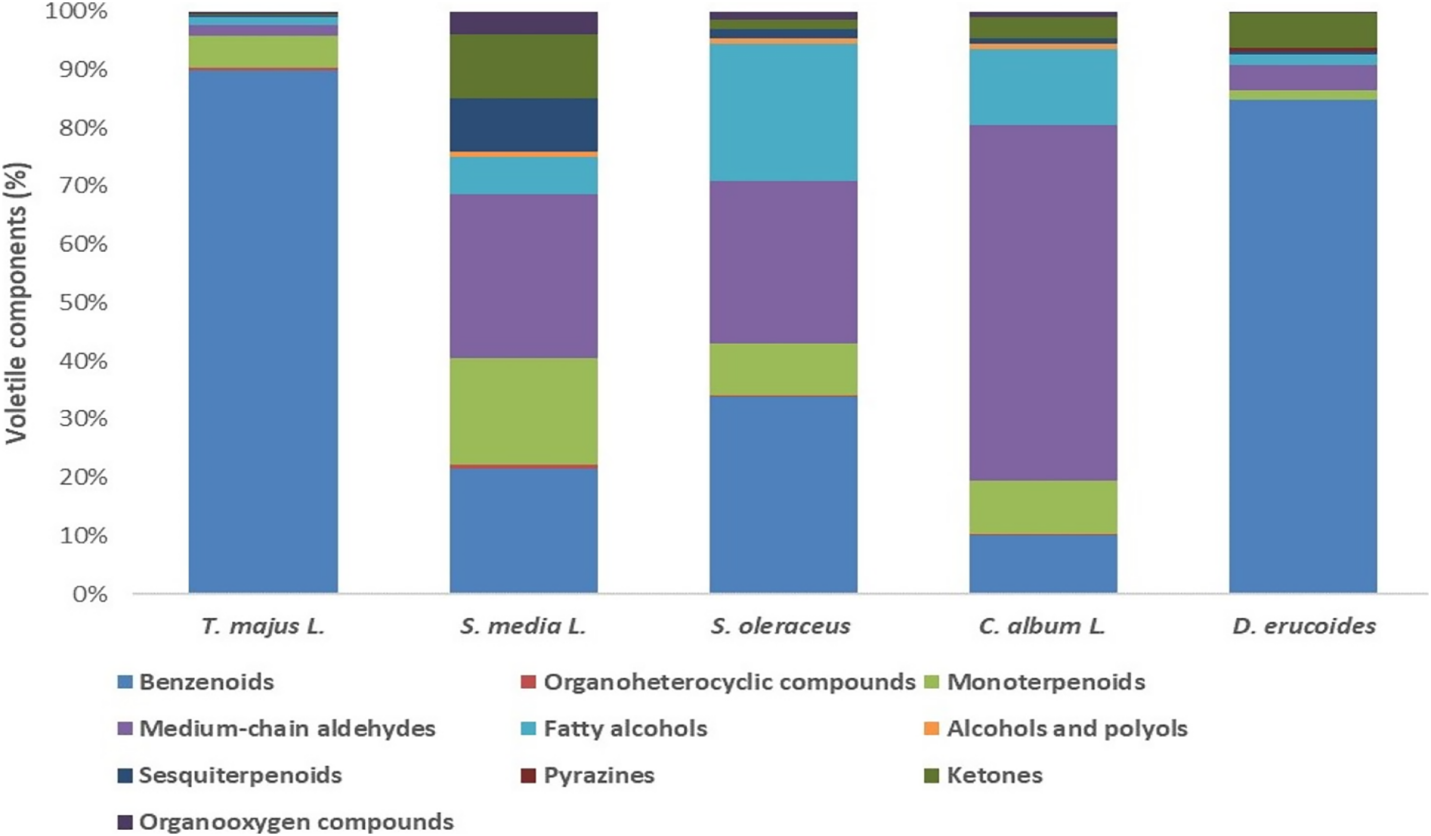
Relative (%) content volatile chemical family of the five green leaves of fresh undervalued plants.

Figure 2 presents the relative percentage of each group of chemical family for the component volatiles detected in the green leaves of fresh plants studied.

A total of 10 chemical families were isolated: benzenoids, monoterpenoids, fatty alcohols, sesquiterpenoids, ketones, organoheterocyclic compounds, medium-chain aldehydes, pyrazines, alcohols and organooxygen compounds. In all the leaves of the analyzed plants, the volatile compounds of the bezenoids chemical family were present and formed the majority in *T. majus* L. (89.9%) and *D. erucoides* L. (84.4%), followed by *S. oleraceus* L. (33.8%) and *S. media* L. (21.6%). For *C. album* L. the majority fraction was medium chain aldehydes with 60.9%, which was also found in representative portions in *S. oleraceus* L. and *S. media* L., with 27.8%. The chemical compounds of fatty alcohols stood out in *S. oleraceus* L. (23.7%), followed by *C. album* L. (13.0%).

Ketones and sesquiterpenes families of chemical compounds were accentuated in *S. media* L., with 10.9% and 9.2%, respectively. In addition, ketones were the second class of volatile components in *D. erucoides* L. (5.9%). A pyrazine presence attracted attention in the edible leaves *D. erucoides* L., which differentiated this species from the others studied. Briefly, the edible leaves of *S. media* L. turned out to be more heterogeneous, and those of *T. majus* L. the most homogeneous in terms of aromatic components present in the volatiles profile.

## Discussion

The five wild edible species examined in this work show a high diversity in their nutritional and chemical composition, bioactive components and aromatic fraction. This study has found that their quali and quantitative parameters could make an important contribution to balancing and rationalizing diets and result in healthy foods.

Some references can be found to the phytochemical studies of these plants, but the data available on their nutritional composition of leaves or volatile profiles are scare. The quality parameters found in this research show that all analyzed species can be considered a good complement in healthy diets.

The studied species did not differ significantly in their moisture and ash contents compared to fresh leaves of the same Spanish species from different Mediterranean areas, ranging from 79.8% (*T.majus* L.) to 87.6% (*S. oleraceus* L.) (Tardío et al., 2016). In *T. majus* L., both parameters were found only for its flowers, showing a wide variation depending on the origin of the species: 0.63% for Mediterranean (Navarro-González et al., 2015) and 5.8% for Polish (Jakubczyk et al., 2018). Water content was relevant in the food composition. In general, moisture content is an important factor that directly affects nutritional content, helps in the digestion and absorption of food and above all, is an index to the freshness of a green leaf plant (Zihad et al., 2019).

With respect to the nutritional compounds, the crude protein content prevailed in *C. album* L. and *D. erucoides* (L.) *DC*, raw fiber prevailed in *C. album* L. and *T. majus* L., and carbohydrates were dominant in *T*. *majus* L. and *S. media* L.

The nutrient profile studied by Tardío et al. (2016) in *C. album* L. was 2.74% (protein), 6.38% (fiber), 0.63% (lipid), and 5.89% (carbohydrate). In the same order in *S. oleraceus* L., the corresponding figures were 2.22%, 3.37%, 0.60% and 2.29%. Both samples came from different Mediterranean areas, although Spanish samples were predominant. For *D. erucoides* L. from Foggia Province (Italy), the protein value was 3.5%, significantly exceeding that established in this study (Disciglio et al., 2017). *S. media* studied in this research was compared with *S. vulgaris*, a Mediterranean wild edible species of the same family. For *S. vulgaris* the nutritional value corresponded to protein 2.47%, fiber 4.36%, lipids 0.67%, and carbohydrate 2.32% (Tardío et al., 2016). Except for the carbohydrate, other parameters significantly exceeded those found in this study.

The nutritional value for leaves of the Brazilian *T. majus* L. corresponded to protein 3.32%, lipids 1.52% and carbohydrates 8.33% (Silva et al., 2018). These parameters are significantly higher values than those obtained in this study. According to Pearson (1976), plant food that provides more than 12% of its caloric value from protein is considered a good source of protein. The studied plants are not an abundant resource in meeting the protein sources that are required by the local people. The lipid content is slightly lower than the reported values (0.63-0.70%) in the same species from other Mediterranean areas (Tardío et al., 2016). However, it is greater in the *T. majus* L. leaves than their flowers. The five species of this study corroborated appreciation that vegetables are considered a minor source of lipids. Dietary fiber is the essential vegetable macronutrient consumed by humans in a normal balanced diet. Two studied species, *S. oleraceus* L. and *T. majus* L., have the highest portion of crude fiber within macronutrients. If compared with the range indicated by Tardío et al. (2016) for *S. oleraceus* from other Mediterranean areas, the value obtained in this study is within this range. The crude fiber for *T. majus* L. flowers, with 5.51%, is similar in the leaves (Navarro-González et al., 2015). In contrast, *S. media* L. showed the lowest fiber content among all, although it was very close to the fiber content in *E. vesicaria*, with 1.6% (Tardío et al., 2016). Three of the wild species included in this study have a prevalence of carbohydrates: *S. media* L., *C. album* L. and *D. erucoides* L. The carbohydrate content found in these species was even higher than in the Mediterranean plants *S. vulgaris* (2.32%), *C. album* L. (5.89%) and *E. vesicaria* (2.1%), reported by Tardío et al. (2016). The carbohydrates fraction of leaves is mainly integrated by simple sugars (fructose, glucose y sucrose), fructose being the most abundant monosaccharide detected in leaves (Hounsome et al., 2008). The crude protein, fat, crude fiber, and carbohydrates content was considered close to the same species from other Mediterranean regions. These results may be due to similar edaphoclimatic conditions. All the species of the present study showed low caloric value, but with a high nutritional density, especially in minerals and fiber. Therefore, these plants can be recommended to low-caloric diets. The nutritional balance, characterized by high fiber and low fat content, indicates that all species studied can be a source of healthy foods (Jimoh, Adedapo & Afolayan, 2011).

For the five undervalued edible species of this study, the mean values of the minerals (macro and micro) that are important for human nutrition were reported. The nutritional power of the five undervalued species studied for their edible leaves lies in their great wealth in minerals. The variability found between the mineral levels is due to the different absorption capacity of each of them, since the environmental conditions were the same due to season and growth area. The most prominent element was potassium. *C. album* L. had a higher content than all. This value agrees with those reported by Tardío et al. (2016) for the same species from other Mediterranean areas. The WHO (2012) reports indicate that potassium content in the green vegetables most consumed per population is approximately 550.0 mg·100 ^g−1^ fw. All studied species exceed this level and *C. album* L. doubles it. The high potassium content makes plants recommendable in diets that require low sodium content. This is even better if the relationship is less than one. The Na/K ratio in the body is a factor to consider in high blood pressure prevention. The all-edible unevaluated species of this study meet this condition (Morrissey et al., 2020).

Magnesium and calcium are regarded as two outstanding minerals. In a comparison with Mediterranean wild greens studied by other authors (García-Herrera et al., 2014; Tardío et al., 2016; Jakubczyk et al., 2018), magnesium reached quite a higher content level in the species presented here. *C. album* L. and *D. erucoides* L. stood out above all. A remarkable contrast of the magnesium content in the leaves of *C. album* L. postulates it as a promising source of the contribution of this cation through intake, so much so that 100 g of fresh leaf of *C. album* L. of the present study would cover the recommended daily dose of this element (around 320 mg/day, FAO/WHO recommendation). In the plant, magnesium has a direct impact on the absorption of solar energy, which in turn is used in the synthesis of carbohydrates and sugars (Waterland et al., 2017). The concentrations of the rest of the macronutrients (Ca, P and Na) are not highlighted in reference to the daily recommended contributions, except for *C. album* L. Its leaves are a rich source of calcium, the content of which may cover between 25–30% of the daily intake recommended by the FAO/WHO (1,000–1,300 mg/day). Highlighting *C. album* L. as the species with the highest mineral concentration, the Chenopodeacea family is very competitive for nutrients and soil water (Akbar Hussain, Sadiq & Zia-Ul-Haq, 2018).

In addition, *C. album* L. showed iron content that approximates to the concentration of spinach with 2.7 mg·100 g^−1^ fw (Farrán, Zamora & Cervera, 2004), which is lower than that found in *C. album* L. (5.29 mg·100 g^−1^ fw) from other Mediterranean areas studied by Tardío et al. (2016). However, considering the nutritional importance of iron, whose deficiency is related to anemia, by including these iron-rich greens in the daily diet, one can easily meet a reasonable amount of the daily requirement of this mineral from one serving. Zinc was the second most abundant micromineral in both *C. album* L. and *S. media* L. It is an especially important mineral to help facilitate normal function of the immune system (Shankar & Prasad, 1998; Jakubczyk et al., 2018), and for the maintenance of growth, development, and skeletal function (Uwitonze et al., 2020). The WHO/FAO considerer the leafy vegetables only modest sources of zinc, having a concentration of <10 mg/kg. The range 0.5–0.8 mg·100 g^−1^ found in this study corroborates this consideration and allows us to suggest the analyzed plants as a potential supply source of this micronutrient. The copper content was relatively low and did not show significant differences between the analysed plants, except for *C. album* L. Its concentration in this study is similar to that found in the *C. album* leaf (0.19 mg·100 g^−1^) studied by Tardío et al. (2016). During the plant growth stages and development, in biological processes, up to 17 key minerals are involved, which are transferred to human nutrition (Waterland et al., 2017). The intake of these minerals, which exist as natural organic complexes, is taken advantage of by the body. The analyzed species provide Fe, Cu and especially Zn, and the intake of their fresh leaves may contribute to covering parts of the recommended daily allowances of these micronutrients.

The effect of nitrates in the body is adverse, and the most important recommendations are aimed at reducing or mitigating the concentration of nitrates that arrive through intake, some through the consumption of fresh green leaves (Raigón et al., 2016). The content of nitrate in the five analysed species showed a different level, which allows them to be classified as follows: *D. erucoides* L. has a very low content of nitrates; *C. album* L. is low in content, and the other species are medium in content (Moreno, Soto & González, 2015). However, these nitrate levels make species safe for daily intake with no apparent risk to health, since they would hardly exceed the consumption recommendations of 3.65 mg of nitrates per kg of body weight (Ekart et al., 2013). In addition, the concentration of nitrates in vegetables varies according to climatic conditions and agronomic crop management, as well as post-harvest storage conditions (Van Velzen et al., 2008).

The acidity of the leaves is an attribute that directly influences taste perception. Only *D. erucoides* L. shows a pH lower than six (pH = 5.72). This provides a slight acid nuance when chewing the fresh leaves, affecting the pleasant taste in the five species analyzed.

Increasingly, scientific importance has been attached to food health. The consumption of different biological sources of antioxidants is suggested to avoid degenerative diseases.

The species *T. majus* L. and *D. erucoides* L. present high levels of total antioxidants (without significant differences between them). In the study by Arellano et al. (2015), in *T. majus* L*.*, flowers were found to have a value of 3,928.23 μm TE/g fw for antioxidants with the same DPPH technique. The levels of antioxidants found in the fresh leaves in this study exceed the amount in the flowers. The study carried out by Disciglio et al. (2017) for the leaves of *D. tenuifolia*, a relative of *D. erucoides* L. by DPPH technique, have reported an amount of 1,373.8 mg TE/kg fw, which is lower than that found in this analysis for *D. erucoides* L. These comparisons allow us to affirm that the studied species enjoy a high antioxidant content, and therefore can serve as a valuable source, not yet appreciated, of these functional compounds.

The antioxidant activity in the food is closely related to the presence of components such as polyphenols. Their antioxidant activity depends on the chemical structures and their concentration in each food (Silva et al., 2018). Data obtained from studied plants showed the highest polyphenol contents, without wide variation between species. Italian samples *S. oleraceus* L. revelated lower phenolic contents (0.061 mg GAE/100 g) than Spanish samples in the study carried out by Morales et al. (2014), which also estimated that this variability could be due to different climatic conditions, as well as genetic differences. Silva et al. (2018) highlighted total phenolic content for the leaves of the Brazilian *T. majus* L. (167.84 mg GAE/100 g), concluding that it presented one of the high values. On the other hand, the same species in this study widely exceeded this value. Additionally, as strong antioxidants, polyphenols are able to scavenge free radicals, processes involved in reducing the risk of cardiovascular diseases (Kantadoung et al., 2018). All the studied species present a prominent position, besides presenting the highest average level observed of this bioactive compound.

The chlorophylls have functional properties, among which is antioxidant activity (Solymosi & Mysliwa-Kurdziel, 2017); they also include magnesium in their chemical structure and form a complex with another microelement such as copper, facilitating its absorption (Marquez & Sinnecker, 2007). The benefits provided by chlorophylls are provided with the intake of fresh plants.

The highest chlorophylls content (a and b) corresponded to *S. oleraceus* L. and consequently the same trend was observed for total chlorophyll. The second species with a high chlorophyll’s concentration was *C. album* L. In the rest of the species, the found values were lower. These photosynthetic pigments indicate the physiological plants state of fresh leaves as being their best sources. The age and anatomy of the leaves, as well as seasonal irradiance changes, influence the chlorophyll content that is related to nutrients’ status by estimation (Silla et al., 2010). In addition, the high chlorophylls content contributes to increased antioxidant activity in plants (Leite et al., 2018). The fresh leaves of all the wild species analyzed in this study are rich in chlorophylls and could be considered a beneficial contribution to healthy food.

The analysis of fresh leaves’ volatiles fraction showed great specificity of biosynthetic processes in nature. The benzenoids class prevailed in the species of this study, being a secondary metabolite produced under conditions of abiotic and biotic stress. Heat stress also potentiates the direct biosynthesis of benzenoids, and their release by leaves is greater than by flowers (Misztal et al., 2015). There are still few systematic studies of benzenoides production by plants. The second major class turned out to be medium chain aldehydes arising from the metabolism of corresponding fatty acids. These provide smells such as fresh and waxy, established in leaves and in fruits (Zanetti et al., 2021). The most easily found is in animal species and in the lipid fraction of certain plants, affecting the gut microbiota by exerting inhibitory effects on bacteria ingestion. Effects on general and gut-associated immune function in animals have been described, but there are few studies on this function in humans (Zentek et al., 2011). The pirazynes are generally considered a key family of taste and greater intensity for smell than exists in nature (Leidinger, 2020). This volatile family was found exclusively *in D. erucoides* L., which confers to this species a certain spicy flavor. Finally, other volatile compounds families such as monoterpenes, ketones and fatty alcohols were found in all species in very defined proportions, which contribute a particular and specific aroma for each. This view can be expounded by the genetic factor of each species, because the edaphoclimatic conditions were the same for all plants (autumn-winter season).

Thus, the results of the food parameters’ quality should be deepened to include better knowledge of these five undervalued edible species. Moreover, new studies focused on relating the chemical analysis of volatile aroma suggested in this work, as well as sensory attributes, may help to clarify the peculiar taste of each studied species.

The PCA makes it possible to relate parameters to each other, such as mineral content, where magnesium stands out, which in turn is related to the content of chlorophylls, carbohydrates and crude fiber. The high levels of these parameters correspond to the typification of the fresh edible leaves of *Chenopodium album* L.

## Conclusions

This research allows us to conclude that the five undervalued species inherent in the autumn-winter period of the Valencian coast are nutritious foods that provide nutrients and bioactive compounds necessary for the normal functioning of the body and to maintain good health.

It was found that the nutrient composition in all the selected species was different. Some plants contained a large amount of fiber, while others contained a greater amount of carbohydrates. Neither showed a good source of protein or lipids. *D. erucoides* (L.) DC was shown to be rich in magnesium, while in calcium content, *T. majus* L. and *S. oleraseus* L. stood out. *C. album* L. was found to have the highest level of these minerals, as well as the highest level of potassium among all other species. The amount of sodium in all was very low, so it can be postulated that these plants would be good in diets that require low sodium content. The species were shown to be a potential source of the microelement’s Fe and Cu, highlighting their content in *C. album* L. We need to underline that all species are a rich source of zinc, which is responsible for many metabolic functions in the human body. All species include various bioactive components such as antioxidants, polyphenols and chlorophylls. *S. oleraceus* L. and *T. majus* L. stand out for their high level of chlorophylls.

Also, to our knowledge, this is the first report to assess the volatiles profile that determines the aromatic characteristics of these plants.

Increasing the consumption of foods of local plant origin, among which are the undervalued species of edible leaf, helps to sustain a healthy diet that provides health benefits and rescues the quality of traditional products. Other important aspects provided by the undervalued species of edible leaf are the taste and smell of gastronomic preparations.

## Supplemental Information

10.7717/peerj.12488/supp-1 Supplemental Information 1 Anova results.

10.7717/peerj.12488/supp-2 Supplemental Information 2 CPA results 1.

10.7717/peerj.12488/supp-3 Supplemental Information 3 CPA results 2.

10.7717/peerj.12488/supp-4 Supplemental Information 4 Volatile results.

## Additional Information and Declarations

### Field Study Permissions

The following information was supplied relating to field study approvals (*i.e*., approving body and any reference numbers): Field experiments were approved by the Free Research of the Universitat Politècnica de València (ID Tesis 13161).

### Data Availability

The following information was supplied regarding data availability: The raw data is available in the Supplemental File.
