# Supplementary material for: Five undervalued edible species inherent to autumn-winter season: nutritional composition, bioactive constituents and volatiles profile
Source: PeerJ. 2021 Nov 23;9:e12488. doi: 10.7717/peerj.12488 (PMC8621719; doi:10.7717/peerj.12488)
Supplement: Supplemental Information 1 [file peerj-09-12488-s001.rtf]

One-Way ANOVA - Ash by Species

Analysis Summary

Dependent variable: Ash
Factor: Species

Number of observations: 15
Number of levels: 5


The StatAdvisor
---------------
   This procedure performs a one-way analysis of variance for Ash.  It
constructs various tests and graphs to compare the mean values of Ash
for the 5 different levels of Species.  The F-test in the ANOVA table
will test whether there are any significant differences amongst the
means.  If there are, the Multiple Range Tests will tell you which
means are significantly different from which others.  If you are
worried about the presence of outliers, choose the Kruskal-Wallis Test
which compares medians instead of means.  The various plots will help
you judge the practical significance of the results, as well as allow
you to look for possible violations of the assumptions underlying the
analysis of variance.  


Summary Statistics for Ash

Species             Count               Average             Variance            Standard deviation  
----------------------------------------------------------------------------------------------------
C. album            3                   3,96467             0,0593703           0,24366             
D.erucoides         3                   2,17667             0,0372493           0,193001            
S.media             3                   2,01067             0,22298             0,472208            
S.oleraceus         3                   2,11367             0,0546043           0,233676            
T.majus             3                   1,86767             0,00122633          0,035019            
----------------------------------------------------------------------------------------------------
Total               15                  2,42667             0,698944            0,836029            

Species             Minimum             Maximum             Range               Stnd. skewness      
----------------------------------------------------------------------------------------------------
C. album            3,821               4,246               0,425               1,22391             
D.erucoides         1,954               2,296               0,342               -1,21528            
S.media             1,567               2,507               0,94                0,35048             
S.oleraceus         1,846               2,277               0,431               -1,13749            
T.majus             1,845               1,908               0,063               1,19672             
----------------------------------------------------------------------------------------------------
Total               1,567               4,246               2,679               2,2064              

Species             Stnd. kurtosis      
----------------------------------------------------------------------------------------------------
C. album                                
D.erucoides                             
S.media                                 
S.oleraceus                             
T.majus                                 
----------------------------------------------------------------------------------------------------
Total               0,512744            


The StatAdvisor
---------------
   This table shows various statistics for Ash for each of the 5
levels of Species.  The one-way analysis of variance is primarily
intended to compare the means of the different levels, listed here
under the Average column.  Select Means Plot from the list of
Graphical Options to display the means graphically.  

WARNING: There is more than a 3 to 1 difference between the smallest
standard deviation and the largest.  This may cause problems since the
analysis of variance assumes that the standard deviations at all
levels are equal.  Select Variance Check from the list of Tabular
Options to run a formal statistical test for differences among the
sigmas.  You may want to consider transforming the values of Ash to
remove any dependence of the standard deviation on the mean.  


ANOVA Table for Ash by Species

                            Analysis of Variance
-----------------------------------------------------------------------------
Source             Sum of Squares     Df  Mean Square    F-Ratio      P-Value
-----------------------------------------------------------------------------
Between groups            9,03435      4      2,25859      30,08       0,0000
Within groups            0,750861     10    0,0750861
-----------------------------------------------------------------------------
Total (Corr.)             9,78521     14


The StatAdvisor
---------------
   The ANOVA table decomposes the variance of Ash into two components:
a between-group component and a within-group component.  The F-ratio,
which in this case equals 30,08, is a ratio of the between-group
estimate to the within-group estimate.  Since the P-value of the
F-test is less than 0,05, there is a statistically significant
difference between the mean Ash from one level of Species to another
at the 95,0% confidence level.  To determine which means are
significantly different from which others, select Multiple Range Tests
from the list of Tabular Options.


Table of Means for Ash by Species
with 95,0 percent LSD intervals
--------------------------------------------------------------------------------
                                       Stnd. error
Species          Count         Mean     (pooled s)    Lower limit    Upper limit
--------------------------------------------------------------------------------
C. album             3      3,96467       0,158205        3,71541        4,21392
D.erucoides          3      2,17667       0,158205        1,92741        2,42592
S.media              3      2,01067       0,158205        1,76141        2,25992
S.oleraceus          3      2,11367       0,158205        1,86441        2,36292
T.majus              3      1,86767       0,158205        1,61841        2,11692
--------------------------------------------------------------------------------
Total               15      2,42667


The StatAdvisor
---------------
   This table shows the mean Ash for each level of Species.  It also
shows the standard error of each mean, which is a measure of its
sampling variability.  The standard error is formed by dividing the
pooled standard deviation by the square root of the number of
observations at each level.  The table also displays an interval
around each mean.  The intervals currently displayed are based on
Fisher's least significant difference (LSD) procedure.  They are
constructed in such a way that if two means are the same, their
intervals will overlap 95,0% of the time.  You can display the
intervals graphically by selecting Means Plot from the list of
Graphical Options.  In the Multiple Range Tests, these intervals are
used to determine which means are significantly different from which
others.


Multiple Range Tests for Ash by Species

--------------------------------------------------------------------------------
Method: 95,0 percent LSD
Species        Count     Mean              Homogeneous Groups
--------------------------------------------------------------------------------
T.majus        3         1,86767           X 
S.media        3         2,01067           X 
S.oleraceus    3         2,11367           X 
D.erucoides    3         2,17667           X 
C. album       3         3,96467            X
--------------------------------------------------------------------------------
Contrast                                   Difference           +/-  Limits
--------------------------------------------------------------------------------
C. album - D.erucoides                    *1,788                0,498514          
C. album - S.media                        *1,954                0,498514          
C. album - S.oleraceus                    *1,851                0,498514          
C. album - T.majus                        *2,097                0,498514          
D.erucoides - S.media                      0,166                0,498514          
D.erucoides - S.oleraceus                  0,063                0,498514          
D.erucoides - T.majus                      0,309                0,498514          
S.media - S.oleraceus                      -0,103               0,498514          
S.media - T.majus                          0,143                0,498514          
S.oleraceus - T.majus                      0,246                0,498514          
--------------------------------------------------------------------------------
* denotes a statistically significant difference.


The StatAdvisor
---------------
   This table applies a multiple comparison procedure to determine
which means are significantly different from which others.  The bottom
half of the output shows the estimated difference between each pair of
means.  An asterisk has been placed next to 4 pairs, indicating that
these pairs show statistically significant differences at the 95,0%
confidence level.  At the top of the page, 2 homogenous groups are
identified using columns of X's.  Within each column, the levels
containing X's form a group of means within which there are no
statistically significant differences.  The method currently being
used to discriminate among the means is Fisher's least significant
difference (LSD) procedure.  With this method, there is a 5,0% risk of
calling each pair of means significantly different when the actual
difference equals 0.  


Variance Check

Cochran's C test: 0,593932   P-Value = 0,135945
Bartlett's test: 2,35397   P-Value = 0,128964
Hartley's test: 181,827
Levene's test: 0,788883   P-Value = 0,558143


The StatAdvisor
---------------
   The four statistics displayed in this table test the null
hypothesis that the standard deviations of Ash within each of the 5
levels of Species is the same.  Of particular interest are the three
P-values.  Since the smallest of the P-values is greater than or equal
to 0,05, there is not a statistically significant difference amongst
the standard deviations at the 95,0% confidence level.  


Kruskal-Wallis Test for Ash by Species

Species             Sample Size         Average Rank
------------------------------------------------------------
C. album            3                   14,0                
D.erucoides         3                   9,0                 
S.media             3                   6,66667             
S.oleraceus         3                   6,66667             
T.majus             3                   3,66667             
------------------------------------------------------------
Test statistic = 8,9   P-Value = 0,0636482


The StatAdvisor
---------------
   The Kruskal-Wallis test tests the null hypothesis that the medians
of Ash within each of the 5 levels of Species are the same.  The data
from all the levels is first combined and ranked from smallest to
largest.  The average rank is then computed for the data at each
level.  Since the P-value is greater than or equal to 0,05, there is
not a statistically significant difference amongst the medians at the
95,0% confidence level.  


One-Way ANOVA - Moisture by Species

Analysis Summary

Dependent variable: Moisture
Factor: Species

Number of observations: 15
Number of levels: 5


The StatAdvisor
---------------
   This procedure performs a one-way analysis of variance for
Moisture.  It constructs various tests and graphs to compare the mean
values of Moisture for the 5 different levels of Species.  The F-test
in the ANOVA table will test whether there are any significant
differences amongst the means.  If there are, the Multiple Range Tests
will tell you which means are significantly different from which
others.  If you are worried about the presence of outliers, choose the
Kruskal-Wallis Test which compares medians instead of means.  The
various plots will help you judge the practical significance of the
results, as well as allow you to look for possible violations of the
assumptions underlying the analysis of variance.  


Summary Statistics for Moisture

Species             Count               Average             Variance            Standard deviation  
----------------------------------------------------------------------------------------------------
C. album            3                   80,1917             0,823694            0,907576            
D.erucoides         3                   88,2703             0,547052            0,73963             
S.media             3                   91,637              4,3291              2,08065             
S.oleraceus         3                   89,2133             1,17427             1,08364             
T.majus             3                   89,5933             0,0598103           0,244562            
----------------------------------------------------------------------------------------------------
Total               15                  87,7811             17,7139             4,20879             

Species             Minimum             Maximum             Range               Stnd. skewness      
----------------------------------------------------------------------------------------------------
C. album            79,266              81,08               1,814               -0,13067            
D.erucoides         87,435              88,842              1,407               -0,990175           
S.media             89,448              93,589              4,141               -0,357747           
S.oleraceus         88,488              90,459              1,971               1,17562             
T.majus             89,352              89,841              0,489               0,0823475           
----------------------------------------------------------------------------------------------------
Total               79,266              93,589              14,323              -1,81706            

Species             Stnd. kurtosis      
----------------------------------------------------------------------------------------------------
C. album                                
D.erucoides                             
S.media                                 
S.oleraceus                             
T.majus                                 
----------------------------------------------------------------------------------------------------
Total               0,354073            


The StatAdvisor
---------------
   This table shows various statistics for Moisture for each of the 5
levels of Species.  The one-way analysis of variance is primarily
intended to compare the means of the different levels, listed here
under the Average column.  Select Means Plot from the list of
Graphical Options to display the means graphically.  

WARNING: There is more than a 3 to 1 difference between the smallest
standard deviation and the largest.  This may cause problems since the
analysis of variance assumes that the standard deviations at all
levels are equal.  Select Variance Check from the list of Tabular
Options to run a formal statistical test for differences among the
sigmas.  You may want to consider transforming the values of Moisture
to remove any dependence of the standard deviation on the mean.  


ANOVA Table for Moisture by Species

                            Analysis of Variance
-----------------------------------------------------------------------------
Source             Sum of Squares     Df  Mean Square    F-Ratio      P-Value
-----------------------------------------------------------------------------
Between groups            234,127      4      58,5317      42,21       0,0000
Within groups             13,8678     10      1,38678
-----------------------------------------------------------------------------
Total (Corr.)             247,995     14


The StatAdvisor
---------------
   The ANOVA table decomposes the variance of Moisture into two
components: a between-group component and a within-group component. 
The F-ratio, which in this case equals 42,2068, is a ratio of the
between-group estimate to the within-group estimate.  Since the
P-value of the F-test is less than 0,05, there is a statistically
significant difference between the mean Moisture from one level of
Species to another at the 95,0% confidence level.  To determine which
means are significantly different from which others, select Multiple
Range Tests from the list of Tabular Options.


Table of Means for Moisture by Species
with 95,0 percent LSD intervals
--------------------------------------------------------------------------------
                                       Stnd. error
Species          Count         Mean     (pooled s)    Lower limit    Upper limit
--------------------------------------------------------------------------------
C. album             3      80,1917       0,679898        79,1205        81,2629
D.erucoides          3      88,2703       0,679898        87,1991        89,3415
S.media              3       91,637       0,679898        90,5658        92,7082
S.oleraceus          3      89,2133       0,679898        88,1421        90,2845
T.majus              3      89,5933       0,679898        88,5221        90,6645
--------------------------------------------------------------------------------
Total               15      87,7811


The StatAdvisor
---------------
   This table shows the mean Moisture for each level of Species.  It
also shows the standard error of each mean, which is a measure of its
sampling variability.  The standard error is formed by dividing the
pooled standard deviation by the square root of the number of
observations at each level.  The table also displays an interval
around each mean.  The intervals currently displayed are based on
Fisher's least significant difference (LSD) procedure.  They are
constructed in such a way that if two means are the same, their
intervals will overlap 95,0% of the time.  You can display the
intervals graphically by selecting Means Plot from the list of
Graphical Options.  In the Multiple Range Tests, these intervals are
used to determine which means are significantly different from which
others.


Multiple Range Tests for Moisture by Species

--------------------------------------------------------------------------------
Method: 95,0 percent LSD
Species        Count     Mean              Homogeneous Groups
--------------------------------------------------------------------------------
C. album       3         80,1917           X  
D.erucoides    3         88,2703            X 
S.oleraceus    3         89,2133            X 
T.majus        3         89,5933            XX
S.media        3         91,637              X
--------------------------------------------------------------------------------
Contrast                                   Difference           +/-  Limits
--------------------------------------------------------------------------------
C. album - D.erucoides                    *-8,07867             2,14241           
C. album - S.media                        *-11,4453             2,14241           
C. album - S.oleraceus                    *-9,02167             2,14241           
C. album - T.majus                        *-9,40167             2,14241           
D.erucoides - S.media                     *-3,36667             2,14241           
D.erucoides - S.oleraceus                  -0,943               2,14241           
D.erucoides - T.majus                      -1,323               2,14241           
S.media - S.oleraceus                     *2,42367              2,14241           
S.media - T.majus                          2,04367              2,14241           
S.oleraceus - T.majus                      -0,38                2,14241           
--------------------------------------------------------------------------------
* denotes a statistically significant difference.


The StatAdvisor
---------------
   This table applies a multiple comparison procedure to determine
which means are significantly different from which others.  The bottom
half of the output shows the estimated difference between each pair of
means.  An asterisk has been placed next to 6 pairs, indicating that
these pairs show statistically significant differences at the 95,0%
confidence level.  At the top of the page, 3 homogenous groups are
identified using columns of X's.  Within each column, the levels
containing X's form a group of means within which there are no
statistically significant differences.  The method currently being
used to discriminate among the means is Fisher's least significant
difference (LSD) procedure.  With this method, there is a 5,0% risk of
calling each pair of means significantly different when the actual
difference equals 0.  


Variance Check

Cochran's C test: 0,624336   P-Value = 0,0995794
Bartlett's test: 2,06377   P-Value = 0,196343
Hartley's test: 72,3804
Levene's test: 0,97195   P-Value = 0,464507


The StatAdvisor
---------------
   The four statistics displayed in this table test the null
hypothesis that the standard deviations of Moisture within each of the
5 levels of Species is the same.  Of particular interest are the three
P-values.  Since the smallest of the P-values is greater than or equal
to 0,05, there is not a statistically significant difference amongst
the standard deviations at the 95,0% confidence level.  


Kruskal-Wallis Test for Moisture by Species

Species             Sample Size         Average Rank
------------------------------------------------------------
C. album            3                   2,0                 
D.erucoides         3                   6,0                 
S.media             3                   13,0                
S.oleraceus         3                   8,33333             
T.majus             3                   10,6667             
------------------------------------------------------------
Test statistic = 10,8333   P-Value = 0,0285024


The StatAdvisor
---------------
   The Kruskal-Wallis test tests the null hypothesis that the medians
of Moisture within each of the 5 levels of Species are the same.  The
data from all the levels is first combined and ranked from smallest to
largest.  The average rank is then computed for the data at each
level.  Since the P-value is less than 0,05, there is a statistically
significant difference amongst the medians at the 95,0% confidence
level.  To determine which medians are significantly different from
which others, select Box-and-Whisker Plot from the list of Graphical
Options and select the median notch option.


One-Way ANOVA - Crude proteins by Species

Analysis Summary

Dependent variable: Crude proteins
Factor: Species

Number of observations: 15
Number of levels: 5


The StatAdvisor
---------------
   This procedure performs a one-way analysis of variance for Crude
proteins.  It constructs various tests and graphs to compare the mean
values of Crude proteins for the 5 different levels of Species.  The
F-test in the ANOVA table will test whether there are any significant
differences amongst the means.  If there are, the Multiple Range Tests
will tell you which means are significantly different from which
others.  If you are worried about the presence of outliers, choose the
Kruskal-Wallis Test which compares medians instead of means.  The
various plots will help you judge the practical significance of the
results, as well as allow you to look for possible violations of the
assumptions underlying the analysis of variance.  


Summary Statistics for Crude proteins

Species             Count               Average             Variance            Standard deviation  
----------------------------------------------------------------------------------------------------
C. album            3                   2,24733             0,00209433          0,0457639           
D.erucoides         3                   2,24733             0,00209433          0,0457639           
S.media             3                   0,201333            0,00223033          0,0472264           
S.oleraceus         3                   1,76067             0,00680633          0,0825005           
T.majus             3                   1,82033             0,00992133          0,0996059           
----------------------------------------------------------------------------------------------------
Total               15                  1,6554              0,614742            0,784055            

Species             Minimum             Maximum             Range               Stnd. skewness      
----------------------------------------------------------------------------------------------------
C. album            2,199               2,29                0,091               -0,387964           
D.erucoides         2,199               2,29                0,091               -0,387964           
S.media             0,157               0,251               0,094               0,354762            
S.oleraceus         1,678               1,843               0,165               -0,0128562          
T.majus             1,709               1,901               0,192               -0,886805           
----------------------------------------------------------------------------------------------------
Total               0,157               2,29                2,133               -2,15021            

Species             Stnd. kurtosis      
----------------------------------------------------------------------------------------------------
C. album                                
D.erucoides                             
S.media                                 
S.oleraceus                             
T.majus                                 
----------------------------------------------------------------------------------------------------
Total               0,290953            


The StatAdvisor
---------------
   This table shows various statistics for Crude proteins for each of
the 5 levels of Species.  The one-way analysis of variance is
primarily intended to compare the means of the different levels,
listed here under the Average column.  Select Means Plot from the list
of Graphical Options to display the means graphically.  


ANOVA Table for Crude proteins by Species

                            Analysis of Variance
-----------------------------------------------------------------------------
Source             Sum of Squares     Df  Mean Square    F-Ratio      P-Value
-----------------------------------------------------------------------------
Between groups            8,56009      4      2,14002     462,27       0,0000
Within groups           0,0462933     10   0,00462933
-----------------------------------------------------------------------------
Total (Corr.)             8,60639     14


The StatAdvisor
---------------
   The ANOVA table decomposes the variance of Crude proteins into two
components: a between-group component and a within-group component. 
The F-ratio, which in this case equals 462,275, is a ratio of the
between-group estimate to the within-group estimate.  Since the
P-value of the F-test is less than 0,05, there is a statistically
significant difference between the mean Crude proteins from one level
of Species to another at the 95,0% confidence level.  To determine
which means are significantly different from which others, select
Multiple Range Tests from the list of Tabular Options.


Table of Means for Crude proteins by Species
with 95,0 percent LSD intervals
--------------------------------------------------------------------------------
                                       Stnd. error
Species          Count         Mean     (pooled s)    Lower limit    Upper limit
--------------------------------------------------------------------------------
C. album             3      2,24733      0,0392825        2,18544        2,30922
D.erucoides          3      2,24733      0,0392825        2,18544        2,30922
S.media              3     0,201333      0,0392825       0,139442       0,263224
S.oleraceus          3      1,76067      0,0392825        1,69878        1,82256
T.majus              3      1,82033      0,0392825        1,75844        1,88222
--------------------------------------------------------------------------------
Total               15       1,6554


The StatAdvisor
---------------
   This table shows the mean Crude proteins for each level of Species.
It also shows the standard error of each mean, which is a measure of
its sampling variability.  The standard error is formed by dividing
the pooled standard deviation by the square root of the number of
observations at each level.  The table also displays an interval
around each mean.  The intervals currently displayed are based on
Fisher's least significant difference (LSD) procedure.  They are
constructed in such a way that if two means are the same, their
intervals will overlap 95,0% of the time.  You can display the
intervals graphically by selecting Means Plot from the list of
Graphical Options.  In the Multiple Range Tests, these intervals are
used to determine which means are significantly different from which
others.


Multiple Range Tests for Crude proteins by Species

--------------------------------------------------------------------------------
Method: 95,0 percent LSD
Species        Count     Mean              Homogeneous Groups
--------------------------------------------------------------------------------
S.media        3         0,201333          X  
S.oleraceus    3         1,76067            X 
T.majus        3         1,82033            X 
D.erucoides    3         2,24733             X
C. album       3         2,24733             X
--------------------------------------------------------------------------------
Contrast                                   Difference           +/-  Limits
--------------------------------------------------------------------------------
C. album - D.erucoides                     0,0                  0,123782          
C. album - S.media                        *2,046                0,123782          
C. album - S.oleraceus                    *0,486667             0,123782          
C. album - T.majus                        *0,427                0,123782          
D.erucoides - S.media                     *2,046                0,123782          
D.erucoides - S.oleraceus                 *0,486667             0,123782          
D.erucoides - T.majus                     *0,427                0,123782          
S.media - S.oleraceus                     *-1,55933             0,123782          
S.media - T.majus                         *-1,619               0,123782          
S.oleraceus - T.majus                      -0,0596667           0,123782          
--------------------------------------------------------------------------------
* denotes a statistically significant difference.


The StatAdvisor
---------------
   This table applies a multiple comparison procedure to determine
which means are significantly different from which others.  The bottom
half of the output shows the estimated difference between each pair of
means.  An asterisk has been placed next to 8 pairs, indicating that
these pairs show statistically significant differences at the 95,0%
confidence level.  At the top of the page, 3 homogenous groups are
identified using columns of X's.  Within each column, the levels
containing X's form a group of means within which there are no
statistically significant differences.  The method currently being
used to discriminate among the means is Fisher's least significant
difference (LSD) procedure.  With this method, there is a 5,0% risk of
calling each pair of means significantly different when the actual
difference equals 0.  


Variance Check

Cochran's C test: 0,428629   P-Value = 0,532896
Bartlett's test: 1,26337   P-Value = 0,745291
Hartley's test: 4,73723
Levene's test: 0,398231   P-Value = 0,805668


The StatAdvisor
---------------
   The four statistics displayed in this table test the null
hypothesis that the standard deviations of Crude proteins within each
of the 5 levels of Species is the same.  Of particular interest are
the three P-values.  Since the smallest of the P-values is greater
than or equal to 0,05, there is not a statistically significant
difference amongst the standard deviations at the 95,0% confidence
level.  


Kruskal-Wallis Test for Crude proteins by Species

Species             Sample Size         Average Rank
------------------------------------------------------------
C. album            3                   12,5                
D.erucoides         3                   12,5                
S.media             3                   2,0                 
S.oleraceus         3                   5,66667             
T.majus             3                   7,33333             
------------------------------------------------------------
Test statistic = 12,4249   P-Value = 0,0144561


The StatAdvisor
---------------
   The Kruskal-Wallis test tests the null hypothesis that the medians
of Crude proteins within each of the 5 levels of Species are the same.
The data from all the levels is first combined and ranked from
smallest to largest.  The average rank is then computed for the data
at each level.  Since the P-value is less than 0,05, there is a
statistically significant difference amongst the medians at the 95,0%
confidence level.  To determine which medians are significantly
different from which others, select Box-and-Whisker Plot from the list
of Graphical Options and select the median notch option.


One-Way ANOVA - Fat by Species

Analysis Summary

Dependent variable: Fat
Factor: Species

Number of observations: 15
Number of levels: 5


The StatAdvisor
---------------
   This procedure performs a one-way analysis of variance for Fat.  It
constructs various tests and graphs to compare the mean values of Fat
for the 5 different levels of Species.  The F-test in the ANOVA table
will test whether there are any significant differences amongst the
means.  If there are, the Multiple Range Tests will tell you which
means are significantly different from which others.  If you are
worried about the presence of outliers, choose the Kruskal-Wallis Test
which compares medians instead of means.  The various plots will help
you judge the practical significance of the results, as well as allow
you to look for possible violations of the assumptions underlying the
analysis of variance.  


Summary Statistics for Fat

Species             Count               Average             Variance            Standard deviation  
----------------------------------------------------------------------------------------------------
C. album            3                   0,251667            0,00119233          0,0345302           
D.erucoides         3                   0,246333            0,00124133          0,0352326           
S.media             3                   0,385333            0,0139423           0,118078            
S.oleraceus         3                   0,347333            0,000346333         0,01861             
T.majus             3                   0,454333            0,0112523           0,106077            
----------------------------------------------------------------------------------------------------
Total               15                  0,337               0,0107919           0,103884            

Species             Minimum             Maximum             Range               Stnd. skewness      
----------------------------------------------------------------------------------------------------
C. album            0,212               0,275               0,063               -1,16836            
D.erucoides         0,209               0,279               0,07                -0,41407            
S.media             0,284               0,515               0,231               0,719569            
S.oleraceus         0,33                0,367               0,037               0,392686            
T.majus             0,346               0,558               0,212               -0,139715           
----------------------------------------------------------------------------------------------------
Total               0,209               0,558               0,349               1,47484             

Species             Stnd. kurtosis      
----------------------------------------------------------------------------------------------------
C. album                                
D.erucoides                             
S.media                                 
S.oleraceus                             
T.majus                                 
----------------------------------------------------------------------------------------------------
Total               0,179804            


The StatAdvisor
---------------
   This table shows various statistics for Fat for each of the 5
levels of Species.  The one-way analysis of variance is primarily
intended to compare the means of the different levels, listed here
under the Average column.  Select Means Plot from the list of
Graphical Options to display the means graphically.  

WARNING: There is more than a 3 to 1 difference between the smallest
standard deviation and the largest.  This may cause problems since the
analysis of variance assumes that the standard deviations at all
levels are equal.  Select Variance Check from the list of Tabular
Options to run a formal statistical test for differences among the
sigmas.  You may want to consider transforming the values of Fat to
remove any dependence of the standard deviation on the mean.  


ANOVA Table for Fat by Species

                            Analysis of Variance
-----------------------------------------------------------------------------
Source             Sum of Squares     Df  Mean Square    F-Ratio      P-Value
-----------------------------------------------------------------------------
Between groups          0,0951367      4    0,0237842       4,25       0,0289
Within groups           0,0559493     10   0,00559493
-----------------------------------------------------------------------------
Total (Corr.)            0,151086     14


The StatAdvisor
---------------
   The ANOVA table decomposes the variance of Fat into two components:
a between-group component and a within-group component.  The F-ratio,
which in this case equals 4,25102, is a ratio of the between-group
estimate to the within-group estimate.  Since the P-value of the
F-test is less than 0,05, there is a statistically significant
difference between the mean Fat from one level of Species to another
at the 95,0% confidence level.  To determine which means are
significantly different from which others, select Multiple Range Tests
from the list of Tabular Options.


Table of Means for Fat by Species
with 95,0 percent LSD intervals
--------------------------------------------------------------------------------
                                       Stnd. error
Species          Count         Mean     (pooled s)    Lower limit    Upper limit
--------------------------------------------------------------------------------
C. album             3     0,251667      0,0431854       0,183627       0,319707
D.erucoides          3     0,246333      0,0431854       0,178293       0,314373
S.media              3     0,385333      0,0431854       0,317293       0,453373
S.oleraceus          3     0,347333      0,0431854       0,279293       0,415373
T.majus              3     0,454333      0,0431854       0,386293       0,522373
--------------------------------------------------------------------------------
Total               15        0,337


The StatAdvisor
---------------
   This table shows the mean Fat for each level of Species.  It also
shows the standard error of each mean, which is a measure of its
sampling variability.  The standard error is formed by dividing the
pooled standard deviation by the square root of the number of
observations at each level.  The table also displays an interval
around each mean.  The intervals currently displayed are based on
Fisher's least significant difference (LSD) procedure.  They are
constructed in such a way that if two means are the same, their
intervals will overlap 95,0% of the time.  You can display the
intervals graphically by selecting Means Plot from the list of
Graphical Options.  In the Multiple Range Tests, these intervals are
used to determine which means are significantly different from which
others.


Multiple Range Tests for Fat by Species

--------------------------------------------------------------------------------
Method: 95,0 percent LSD
Species        Count     Mean              Homogeneous Groups
--------------------------------------------------------------------------------
D.erucoides    3         0,246333          X  
C. album       3         0,251667          XX 
S.oleraceus    3         0,347333          XXX
S.media        3         0,385333           XX
T.majus        3         0,454333            X
--------------------------------------------------------------------------------
Contrast                                   Difference           +/-  Limits
--------------------------------------------------------------------------------
C. album - D.erucoides                     0,00533333           0,13608           
C. album - S.media                         -0,133667            0,13608           
C. album - S.oleraceus                     -0,0956667           0,13608           
C. album - T.majus                        *-0,202667            0,13608           
D.erucoides - S.media                     *-0,139               0,13608           
D.erucoides - S.oleraceus                  -0,101               0,13608           
D.erucoides - T.majus                     *-0,208               0,13608           
S.media - S.oleraceus                      0,038                0,13608           
S.media - T.majus                          -0,069               0,13608           
S.oleraceus - T.majus                      -0,107               0,13608           
--------------------------------------------------------------------------------
* denotes a statistically significant difference.


The StatAdvisor
---------------
   This table applies a multiple comparison procedure to determine
which means are significantly different from which others.  The bottom
half of the output shows the estimated difference between each pair of
means.  An asterisk has been placed next to 3 pairs, indicating that
these pairs show statistically significant differences at the 95,0%
confidence level.  At the top of the page, 3 homogenous groups are
identified using columns of X's.  Within each column, the levels
containing X's form a group of means within which there are no
statistically significant differences.  The method currently being
used to discriminate among the means is Fisher's least significant
difference (LSD) procedure.  With this method, there is a 5,0% risk of
calling each pair of means significantly different when the actual
difference equals 0.  


Variance Check

Cochran's C test: 0,498391   P-Value = 0,316541
Bartlett's test: 2,32661   P-Value = 0,133958
Hartley's test: 40,257
Levene's test: 1,20327   P-Value = 0,367992


The StatAdvisor
---------------
   The four statistics displayed in this table test the null
hypothesis that the standard deviations of Fat within each of the 5
levels of Species is the same.  Of particular interest are the three
P-values.  Since the smallest of the P-values is greater than or equal
to 0,05, there is not a statistically significant difference amongst
the standard deviations at the 95,0% confidence level.  


Kruskal-Wallis Test for Fat by Species

Species             Sample Size         Average Rank
------------------------------------------------------------
C. album            3                   3,66667             
D.erucoides         3                   3,33333             
S.media             3                   10,6667             
S.oleraceus         3                   9,66667             
T.majus             3                   12,6667             
------------------------------------------------------------
Test statistic = 10,8333   P-Value = 0,0285024


The StatAdvisor
---------------
   The Kruskal-Wallis test tests the null hypothesis that the medians
of Fat within each of the 5 levels of Species are the same.  The data
from all the levels is first combined and ranked from smallest to
largest.  The average rank is then computed for the data at each
level.  Since the P-value is less than 0,05, there is a statistically
significant difference amongst the medians at the 95,0% confidence
level.  To determine which medians are significantly different from
which others, select Box-and-Whisker Plot from the list of Graphical
Options and select the median notch option.


One-Way ANOVA - Crude fiber by Species

Analysis Summary

Dependent variable: Crude fiber
Factor: Species

Number of observations: 15
Number of levels: 5


The StatAdvisor
---------------
   This procedure performs a one-way analysis of variance for Crude
fiber.  It constructs various tests and graphs to compare the mean
values of Crude fiber for the 5 different levels of Species.  The
F-test in the ANOVA table will test whether there are any significant
differences amongst the means.  If there are, the Multiple Range Tests
will tell you which means are significantly different from which
others.  If you are worried about the presence of outliers, choose the
Kruskal-Wallis Test which compares medians instead of means.  The
various plots will help you judge the practical significance of the
results, as well as allow you to look for possible violations of the
assumptions underlying the analysis of variance.  


Summary Statistics for Crude fiber

Species             Count               Average             Variance            Standard deviation  
----------------------------------------------------------------------------------------------------
C. album            3                   5,40367             0,195806            0,4425              
D.erucoides         3                   2,92933             0,11908             0,34508             
S.media             3                   1,21733             0,173285            0,416276            
S.oleraceus         3                   3,66233             0,717894            0,847286            
T.majus             3                   5,08233             0,3798              0,616279            
----------------------------------------------------------------------------------------------------
Total               15                  3,659               2,70453             1,64455             

Species             Minimum             Maximum             Range               Stnd. skewness      
----------------------------------------------------------------------------------------------------
C. album            4,898               5,72                0,822               -1,11223            
D.erucoides         2,543               3,207               0,664               -0,902651           
S.media             0,94                1,696               0,756               1,17898             
S.oleraceus         2,684               4,158               1,474               -1,22442            
T.majus             4,39                5,571               1,181               -0,936726           
----------------------------------------------------------------------------------------------------
Total               0,94                5,72                4,78                -0,555509           

Species             Stnd. kurtosis      
----------------------------------------------------------------------------------------------------
C. album                                
D.erucoides                             
S.media                                 
S.oleraceus                             
T.majus                                 
----------------------------------------------------------------------------------------------------
Total               -0,888128           


The StatAdvisor
---------------
   This table shows various statistics for Crude fiber for each of the
5 levels of Species.  The one-way analysis of variance is primarily
intended to compare the means of the different levels, listed here
under the Average column.  Select Means Plot from the list of
Graphical Options to display the means graphically.  


ANOVA Table for Crude fiber by Species

                            Analysis of Variance
-----------------------------------------------------------------------------
Source             Sum of Squares     Df  Mean Square    F-Ratio      P-Value
-----------------------------------------------------------------------------
Between groups            34,6917      4      8,67293      27,34       0,0000
Within groups             3,17173     10     0,317173
-----------------------------------------------------------------------------
Total (Corr.)             37,8634     14


The StatAdvisor
---------------
   The ANOVA table decomposes the variance of Crude fiber into two
components: a between-group component and a within-group component. 
The F-ratio, which in this case equals 27,3444, is a ratio of the
between-group estimate to the within-group estimate.  Since the
P-value of the F-test is less than 0,05, there is a statistically
significant difference between the mean Crude fiber from one level of
Species to another at the 95,0% confidence level.  To determine which
means are significantly different from which others, select Multiple
Range Tests from the list of Tabular Options.


Table of Means for Crude fiber by Species
with 95,0 percent LSD intervals
--------------------------------------------------------------------------------
                                       Stnd. error
Species          Count         Mean     (pooled s)    Lower limit    Upper limit
--------------------------------------------------------------------------------
C. album             3      5,40367       0,325153        4,89138        5,91596
D.erucoides          3      2,92933       0,325153        2,41704        3,44162
S.media              3      1,21733       0,325153       0,705043        1,72962
S.oleraceus          3      3,66233       0,325153        3,15004        4,17462
T.majus              3      5,08233       0,325153        4,57004        5,59462
--------------------------------------------------------------------------------
Total               15        3,659


The StatAdvisor
---------------
   This table shows the mean Crude fiber for each level of Species. 
It also shows the standard error of each mean, which is a measure of
its sampling variability.  The standard error is formed by dividing
the pooled standard deviation by the square root of the number of
observations at each level.  The table also displays an interval
around each mean.  The intervals currently displayed are based on
Fisher's least significant difference (LSD) procedure.  They are
constructed in such a way that if two means are the same, their
intervals will overlap 95,0% of the time.  You can display the
intervals graphically by selecting Means Plot from the list of
Graphical Options.  In the Multiple Range Tests, these intervals are
used to determine which means are significantly different from which
others.


Multiple Range Tests for Crude fiber by Species

--------------------------------------------------------------------------------
Method: 95,0 percent LSD
Species        Count     Mean              Homogeneous Groups
--------------------------------------------------------------------------------
S.media        3         1,21733           X  
D.erucoides    3         2,92933            X 
S.oleraceus    3         3,66233            X 
T.majus        3         5,08233             X
C. album       3         5,40367             X
--------------------------------------------------------------------------------
Contrast                                   Difference           +/-  Limits
--------------------------------------------------------------------------------
C. album - D.erucoides                    *2,47433              1,02458           
C. album - S.media                        *4,18633              1,02458           
C. album - S.oleraceus                    *1,74133              1,02458           
C. album - T.majus                         0,321333             1,02458           
D.erucoides - S.media                     *1,712                1,02458           
D.erucoides - S.oleraceus                  -0,733               1,02458           
D.erucoides - T.majus                     *-2,153               1,02458           
S.media - S.oleraceus                     *-2,445               1,02458           
S.media - T.majus                         *-3,865               1,02458           
S.oleraceus - T.majus                     *-1,42                1,02458           
--------------------------------------------------------------------------------
* denotes a statistically significant difference.


The StatAdvisor
---------------
   This table applies a multiple comparison procedure to determine
which means are significantly different from which others.  The bottom
half of the output shows the estimated difference between each pair of
means.  An asterisk has been placed next to 8 pairs, indicating that
these pairs show statistically significant differences at the 95,0%
confidence level.  At the top of the page, 3 homogenous groups are
identified using columns of X's.  Within each column, the levels
containing X's form a group of means within which there are no
statistically significant differences.  The method currently being
used to discriminate among the means is Fisher's least significant
difference (LSD) procedure.  With this method, there is a 5,0% risk of
calling each pair of means significantly different when the actual
difference equals 0.  


Variance Check

Cochran's C test: 0,452683   P-Value = 0,44867
Bartlett's test: 1,23848   P-Value = 0,77571
Hartley's test: 6,02866
Levene's test: 0,152668   P-Value = 0,957462


The StatAdvisor
---------------
   The four statistics displayed in this table test the null
hypothesis that the standard deviations of Crude fiber within each of
the 5 levels of Species is the same.  Of particular interest are the
three P-values.  Since the smallest of the P-values is greater than or
equal to 0,05, there is not a statistically significant difference
amongst the standard deviations at the 95,0% confidence level.  


Kruskal-Wallis Test for Crude fiber by Species

Species             Sample Size         Average Rank
------------------------------------------------------------
C. album            3                   13,3333             
D.erucoides         3                   5,66667             
S.media             3                   2,0                 
S.oleraceus         3                   7,33333             
T.majus             3                   11,6667             
------------------------------------------------------------
Test statistic = 12,5667   P-Value = 0,0135992


The StatAdvisor
---------------
   The Kruskal-Wallis test tests the null hypothesis that the medians
of Crude fiber within each of the 5 levels of Species are the same. 
The data from all the levels is first combined and ranked from
smallest to largest.  The average rank is then computed for the data
at each level.  Since the P-value is less than 0,05, there is a
statistically significant difference amongst the medians at the 95,0%
confidence level.  To determine which medians are significantly
different from which others, select Box-and-Whisker Plot from the list
of Graphical Options and select the median notch option.


One-Way ANOVA - Carbohyrates by Species

Analysis Summary

Dependent variable: Carbohyrates
Factor: Species

Number of observations: 15
Number of levels: 5


The StatAdvisor
---------------
   This procedure performs a one-way analysis of variance for
Carbohyrates.  It constructs various tests and graphs to compare the
mean values of Carbohyrates for the 5 different levels of Species. 
The F-test in the ANOVA table will test whether there are any
significant differences amongst the means.  If there are, the Multiple
Range Tests will tell you which means are significantly different from
which others.  If you are worried about the presence of outliers,
choose the Kruskal-Wallis Test which compares medians instead of
means.  The various plots will help you judge the practical
significance of the results, as well as allow you to look for possible
violations of the assumptions underlying the analysis of variance.  


Summary Statistics for Carbohyrates

Species             Count               Average             Variance            Standard deviation  
----------------------------------------------------------------------------------------------------
C. album            3                   7,94033             0,132561            0,36409             
D.erucoides         3                   4,13067             0,406408            0,637502            
S.media             3                   4,54867             1,66729             1,29124             
S.oleraceus         3                   2,90267             0,0239843           0,154869            
T.majus             3                   1,18233             0,11663             0,341512            
----------------------------------------------------------------------------------------------------
Total               15                  4,14093             5,6685              2,38086             

Species             Minimum             Maximum             Range               Stnd. skewness      
----------------------------------------------------------------------------------------------------
C. album            7,673               8,355               0,682               1,07678             
D.erucoides         3,494               4,769               1,275               0,00831883          
S.media             3,233               5,814               2,581               -0,123847           
S.oleraceus         2,802               3,081               0,279               1,19443             
T.majus             0,801               1,46                0,659               -0,876896           
----------------------------------------------------------------------------------------------------
Total               0,801               8,355               7,554               0,829924            

Species             Stnd. kurtosis      
----------------------------------------------------------------------------------------------------
C. album                                
D.erucoides                             
S.media                                 
S.oleraceus                             
T.majus                                 
----------------------------------------------------------------------------------------------------
Total               -0,525445           


The StatAdvisor
---------------
   This table shows various statistics for Carbohyrates for each of
the 5 levels of Species.  The one-way analysis of variance is
primarily intended to compare the means of the different levels,
listed here under the Average column.  Select Means Plot from the list
of Graphical Options to display the means graphically.  

WARNING: There is more than a 3 to 1 difference between the smallest
standard deviation and the largest.  This may cause problems since the
analysis of variance assumes that the standard deviations at all
levels are equal.  Select Variance Check from the list of Tabular
Options to run a formal statistical test for differences among the
sigmas.  You may want to consider transforming the values of
Carbohyrates to remove any dependence of the standard deviation on the
mean.  


Table of Means for Carbohyrates by Species
with 95,0 percent LSD intervals
--------------------------------------------------------------------------------
                                       Stnd. error
Species          Count         Mean     (pooled s)    Lower limit    Upper limit
--------------------------------------------------------------------------------
C. album             3      7,94033       0,395548        7,31713        8,56353
D.erucoides          3      4,13067       0,395548        3,50747        4,75387
S.media              3      4,54867       0,395548        3,92547        5,17187
S.oleraceus          3      2,90267       0,395548        2,27947        3,52587
T.majus              3      1,18233       0,395548       0,559133        1,80553
--------------------------------------------------------------------------------
Total               15      4,14093


The StatAdvisor
---------------
   This table shows the mean Carbohyrates for each level of Species. 
It also shows the standard error of each mean, which is a measure of
its sampling variability.  The standard error is formed by dividing
the pooled standard deviation by the square root of the number of
observations at each level.  The table also displays an interval
around each mean.  The intervals currently displayed are based on
Fisher's least significant difference (LSD) procedure.  They are
constructed in such a way that if two means are the same, their
intervals will overlap 95,0% of the time.  You can display the
intervals graphically by selecting Means Plot from the list of
Graphical Options.  In the Multiple Range Tests, these intervals are
used to determine which means are significantly different from which
others.


Multiple Range Tests for Carbohyrates by Species

--------------------------------------------------------------------------------
Method: 95,0 percent LSD
Species        Count     Mean              Homogeneous Groups
--------------------------------------------------------------------------------
T.majus        3         1,18233           X   
S.oleraceus    3         2,90267            X  
D.erucoides    3         4,13067            XX 
S.media        3         4,54867             X 
C. album       3         7,94033              X
--------------------------------------------------------------------------------
Contrast                                   Difference           +/-  Limits
--------------------------------------------------------------------------------
C. album - D.erucoides                    *3,80967              1,2464            
C. album - S.media                        *3,39167              1,2464            
C. album - S.oleraceus                    *5,03767              1,2464            
C. album - T.majus                        *6,758                1,2464            
D.erucoides - S.media                      -0,418               1,2464            
D.erucoides - S.oleraceus                  1,228                1,2464            
D.erucoides - T.majus                     *2,94833              1,2464            
S.media - S.oleraceus                     *1,646                1,2464            
S.media - T.majus                         *3,36633              1,2464            
S.oleraceus - T.majus                     *1,72033              1,2464            
--------------------------------------------------------------------------------
* denotes a statistically significant difference.


The StatAdvisor
---------------
   This table applies a multiple comparison procedure to determine
which means are significantly different from which others.  The bottom
half of the output shows the estimated difference between each pair of
means.  An asterisk has been placed next to 8 pairs, indicating that
these pairs show statistically significant differences at the 95,0%
confidence level.  At the top of the page, 4 homogenous groups are
identified using columns of X's.  Within each column, the levels
containing X's form a group of means within which there are no
statistically significant differences.  The method currently being
used to discriminate among the means is Fisher's least significant
difference (LSD) procedure.  With this method, there is a 5,0% risk of
calling each pair of means significantly different when the actual
difference equals 0.  


Variance Check

Cochran's C test: 0,71043   P-Value = 0,0351547
Bartlett's test: 2,46317   P-Value = 0,111178
Hartley's test: 69,5158
Levene's test: 1,57496   P-Value = 0,254869


The StatAdvisor
---------------
   The four statistics displayed in this table test the null
hypothesis that the standard deviations of Carbohyrates within each of
the 5 levels of Species is the same.  Of particular interest are the
three P-values.  Since the smallest of the P-values is less than 0,05,
there is a statistically significant difference amongst the standard
deviations at the 95,0% confidence level.  This violates one of the
important assumptions underlying the analysis of variance and will
invalidate most of the standard statistical tests.  


Kruskal-Wallis Test for Carbohyrates by Species

Species             Sample Size         Average Rank
------------------------------------------------------------
C. album            3                   14,0                
D.erucoides         3                   9,33333             
S.media             3                   9,66667             
S.oleraceus         3                   5,0                 
T.majus             3                   2,0                 
------------------------------------------------------------
Test statistic = 12,8333   P-Value = 0,0121195


The StatAdvisor
---------------
   The Kruskal-Wallis test tests the null hypothesis that the medians
of Carbohyrates within each of the 5 levels of Species are the same. 
The data from all the levels is first combined and ranked from
smallest to largest.  The average rank is then computed for the data
at each level.  Since the P-value is less than 0,05, there is a
statistically significant difference amongst the medians at the 95,0%
confidence level.  To determine which medians are significantly
different from which others, select Box-and-Whisker Plot from the list
of Graphical Options and select the median notch option.


One-Way ANOVA - Calcium by Species

Analysis Summary

Dependent variable: Calcium
Factor: Species

Number of observations: 15
Number of levels: 5


The StatAdvisor
---------------
   This procedure performs a one-way analysis of variance for Calcium.
It constructs various tests and graphs to compare the mean values of
Calcium for the 5 different levels of Species.  The F-test in the
ANOVA table will test whether there are any significant differences
amongst the means.  If there are, the Multiple Range Tests will tell
you which means are significantly different from which others.  If you
are worried about the presence of outliers, choose the Kruskal-Wallis
Test which compares medians instead of means.  The various plots will
help you judge the practical significance of the results, as well as
allow you to look for possible violations of the assumptions
underlying the analysis of variance.  


Summary Statistics for Calcium

Species             Count               Average             Variance            Standard deviation  
----------------------------------------------------------------------------------------------------
C. album            3                   313,205             2550,41             50,5016             
D.erucoides         3                   59,9947             741,34              27,2276             
S.media             3                   71,5357             462,513             21,5061             
S.oleraceus         3                   119,226             72,2128             8,49781             
T.majus             3                   119,633             164,463             12,8243             
----------------------------------------------------------------------------------------------------
Total               15                  136,719             9544,55             97,6962             

Species             Minimum             Maximum             Range               Stnd. skewness      
----------------------------------------------------------------------------------------------------
C. album            263,161             364,152             100,991             0,0569196           
D.erucoides         38,587              90,639              52,052              0,955227            
S.media             47,71               89,512              41,802              -0,801428           
S.oleraceus         109,745             126,157             16,412              -0,868678           
T.majus             104,851             127,787             22,936              -1,20526            
----------------------------------------------------------------------------------------------------
Total               38,587              364,152             325,565             2,29795             

Species             Stnd. kurtosis      
----------------------------------------------------------------------------------------------------
C. album                                
D.erucoides                             
S.media                                 
S.oleraceus                             
T.majus                                 
----------------------------------------------------------------------------------------------------
Total               0,944625            


The StatAdvisor
---------------
   This table shows various statistics for Calcium for each of the 5
levels of Species.  The one-way analysis of variance is primarily
intended to compare the means of the different levels, listed here
under the Average column.  Select Means Plot from the list of
Graphical Options to display the means graphically.  

WARNING: There is more than a 3 to 1 difference between the smallest
standard deviation and the largest.  This may cause problems since the
analysis of variance assumes that the standard deviations at all
levels are equal.  Select Variance Check from the list of Tabular
Options to run a formal statistical test for differences among the
sigmas.  You may want to consider transforming the values of Calcium
to remove any dependence of the standard deviation on the mean.  


ANOVA Table for Calcium by Species

                            Analysis of Variance
-----------------------------------------------------------------------------
Source             Sum of Squares     Df  Mean Square    F-Ratio      P-Value
-----------------------------------------------------------------------------
Between groups           125642,0      4      31410,5      39,35       0,0000
Within groups             7981,87     10      798,187
-----------------------------------------------------------------------------
Total (Corr.)            133624,0     14


The StatAdvisor
---------------
   The ANOVA table decomposes the variance of Calcium into two
components: a between-group component and a within-group component. 
The F-ratio, which in this case equals 39,3523, is a ratio of the
between-group estimate to the within-group estimate.  Since the
P-value of the F-test is less than 0,05, there is a statistically
significant difference between the mean Calcium from one level of
Species to another at the 95,0% confidence level.  To determine which
means are significantly different from which others, select Multiple
Range Tests from the list of Tabular Options.


Table of Means for Calcium by Species
with 95,0 percent LSD intervals
--------------------------------------------------------------------------------
                                       Stnd. error
Species          Count         Mean     (pooled s)    Lower limit    Upper limit
--------------------------------------------------------------------------------
C. album             3      313,205        16,3114        287,505        338,904
D.erucoides          3      59,9947        16,3114        34,2954        85,6939
S.media              3      71,5357        16,3114        45,8364        97,2349
S.oleraceus          3      119,226        16,3114        93,5264        144,925
T.majus              3      119,633        16,3114        93,9338        145,332
--------------------------------------------------------------------------------
Total               15      136,719


The StatAdvisor
---------------
   This table shows the mean Calcium for each level of Species.  It
also shows the standard error of each mean, which is a measure of its
sampling variability.  The standard error is formed by dividing the
pooled standard deviation by the square root of the number of
observations at each level.  The table also displays an interval
around each mean.  The intervals currently displayed are based on
Fisher's least significant difference (LSD) procedure.  They are
constructed in such a way that if two means are the same, their
intervals will overlap 95,0% of the time.  You can display the
intervals graphically by selecting Means Plot from the list of
Graphical Options.  In the Multiple Range Tests, these intervals are
used to determine which means are significantly different from which
others.


Multiple Range Tests for Calcium by Species

--------------------------------------------------------------------------------
Method: 95,0 percent LSD
Species        Count     Mean              Homogeneous Groups
--------------------------------------------------------------------------------
D.erucoides    3         59,9947           X  
S.media        3         71,5357           XX 
S.oleraceus    3         119,226            X 
T.majus        3         119,633            X 
C. album       3         313,205             X
--------------------------------------------------------------------------------
Contrast                                   Difference           +/-  Limits
--------------------------------------------------------------------------------
C. album - D.erucoides                    *253,21               51,3984           
C. album - S.media                        *241,669              51,3984           
C. album - S.oleraceus                    *193,979              51,3984           
C. album - T.majus                        *193,572              51,3984           
D.erucoides - S.media                      -11,541              51,3984           
D.erucoides - S.oleraceus                 *-59,231              51,3984           
D.erucoides - T.majus                     *-59,6383             51,3984           
S.media - S.oleraceus                      -47,69               51,3984           
S.media - T.majus                          -48,0973             51,3984           
S.oleraceus - T.majus                      -0,407333            51,3984           
--------------------------------------------------------------------------------
* denotes a statistically significant difference.


The StatAdvisor
---------------
   This table applies a multiple comparison procedure to determine
which means are significantly different from which others.  The bottom
half of the output shows the estimated difference between each pair of
means.  An asterisk has been placed next to 6 pairs, indicating that
these pairs show statistically significant differences at the 95,0%
confidence level.  At the top of the page, 3 homogenous groups are
identified using columns of X's.  Within each column, the levels
containing X's form a group of means within which there are no
statistically significant differences.  The method currently being
used to discriminate among the means is Fisher's least significant
difference (LSD) procedure.  With this method, there is a 5,0% risk of
calling each pair of means significantly different when the actual
difference equals 0.  


Variance Check

Cochran's C test: 0,63905   P-Value = 0,0848707
Bartlett's test: 1,98984   P-Value = 0,219931
Hartley's test: 35,318
Levene's test: 1,11705   P-Value = 0,4013


The StatAdvisor
---------------
   The four statistics displayed in this table test the null
hypothesis that the standard deviations of Calcium within each of the
5 levels of Species is the same.  Of particular interest are the three
P-values.  Since the smallest of the P-values is greater than or equal
to 0,05, there is not a statistically significant difference amongst
the standard deviations at the 95,0% confidence level.  


Kruskal-Wallis Test for Calcium by Species

Species             Sample Size         Average Rank
------------------------------------------------------------
C. album            3                   14,0                
D.erucoides         3                   3,33333             
S.media             3                   3,66667             
S.oleraceus         3                   9,0                 
T.majus             3                   10,0                
------------------------------------------------------------
Test statistic = 12,2333   P-Value = 0,0156979


The StatAdvisor
---------------
   The Kruskal-Wallis test tests the null hypothesis that the medians
of Calcium within each of the 5 levels of Species are the same.  The
data from all the levels is first combined and ranked from smallest to
largest.  The average rank is then computed for the data at each
level.  Since the P-value is less than 0,05, there is a statistically
significant difference amongst the medians at the 95,0% confidence
level.  To determine which medians are significantly different from
which others, select Box-and-Whisker Plot from the list of Graphical
Options and select the median notch option.


One-Way ANOVA - Magnesium by Species

Analysis Summary

Dependent variable: Magnesium
Factor: Species

Number of observations: 15
Number of levels: 5


The StatAdvisor
---------------
   This procedure performs a one-way analysis of variance for
Magnesium.  It constructs various tests and graphs to compare the mean
values of Magnesium for the 5 different levels of Species.  The F-test
in the ANOVA table will test whether there are any significant
differences amongst the means.  If there are, the Multiple Range Tests
will tell you which means are significantly different from which
others.  If you are worried about the presence of outliers, choose the
Kruskal-Wallis Test which compares medians instead of means.  The
various plots will help you judge the practical significance of the
results, as well as allow you to look for possible violations of the
assumptions underlying the analysis of variance.  


Summary Statistics for Magnesium

Species             Count               Average             Variance            Standard deviation  
----------------------------------------------------------------------------------------------------
C. album            3                   480,587             11525,1             107,355             
D.erucoides         3                   114,074             692,489             26,3152             
S.media             3                   82,588              402,162             20,054              
S.oleraceus         3                   78,7007             79,0215             8,88941             
T.majus             3                   67,4143             81,308              9,01709             
----------------------------------------------------------------------------------------------------
Total               15                  164,673             28815,1             169,75              

Species             Minimum             Maximum             Range               Stnd. skewness      
----------------------------------------------------------------------------------------------------
C. album            367,865             581,618             213,753             -0,342409           
D.erucoides         86,696              139,179             52,483              -0,272717           
S.media             63,428              103,43              40,002              0,265007            
S.oleraceus         68,745              85,843              17,098              -0,906173           
T.majus             61,19               77,755              16,565              1,14987             
----------------------------------------------------------------------------------------------------
Total               61,19               581,618             520,428             2,89912             

Species             Stnd. kurtosis      
----------------------------------------------------------------------------------------------------
C. album                                
D.erucoides                             
S.media                                 
S.oleraceus                             
T.majus                                 
----------------------------------------------------------------------------------------------------
Total               1,63484             


The StatAdvisor
---------------
   This table shows various statistics for Magnesium for each of the 5
levels of Species.  The one-way analysis of variance is primarily
intended to compare the means of the different levels, listed here
under the Average column.  Select Means Plot from the list of
Graphical Options to display the means graphically.  

WARNING: There is more than a 3 to 1 difference between the smallest
standard deviation and the largest.  This may cause problems since the
analysis of variance assumes that the standard deviations at all
levels are equal.  Select Variance Check from the list of Tabular
Options to run a formal statistical test for differences among the
sigmas.  You may want to consider transforming the values of Magnesium
to remove any dependence of the standard deviation on the mean.  


ANOVA Table for Magnesium by Species

                            Analysis of Variance
-----------------------------------------------------------------------------
Source             Sum of Squares     Df  Mean Square    F-Ratio      P-Value
-----------------------------------------------------------------------------
Between groups           377851,0      4      94462,8      36,96       0,0000
Within groups             25560,2     10      2556,02
-----------------------------------------------------------------------------
Total (Corr.)            403411,0     14


The StatAdvisor
---------------
   The ANOVA table decomposes the variance of Magnesium into two
components: a between-group component and a within-group component. 
The F-ratio, which in this case equals 36,9571, is a ratio of the
between-group estimate to the within-group estimate.  Since the
P-value of the F-test is less than 0,05, there is a statistically
significant difference between the mean Magnesium from one level of
Species to another at the 95,0% confidence level.  To determine which
means are significantly different from which others, select Multiple
Range Tests from the list of Tabular Options.


Table of Means for Magnesium by Species
with 95,0 percent LSD intervals
--------------------------------------------------------------------------------
                                       Stnd. error
Species          Count         Mean     (pooled s)    Lower limit    Upper limit
--------------------------------------------------------------------------------
C. album             3      480,587        29,1891        434,598        526,576
D.erucoides          3      114,074        29,1891        68,0852        160,062
S.media              3       82,588        29,1891        36,5995        128,577
S.oleraceus          3      78,7007        29,1891        32,7122        124,689
T.majus              3      67,4143        29,1891        21,4258        113,403
--------------------------------------------------------------------------------
Total               15      164,673


The StatAdvisor
---------------
   This table shows the mean Magnesium for each level of Species.  It
also shows the standard error of each mean, which is a measure of its
sampling variability.  The standard error is formed by dividing the
pooled standard deviation by the square root of the number of
observations at each level.  The table also displays an interval
around each mean.  The intervals currently displayed are based on
Fisher's least significant difference (LSD) procedure.  They are
constructed in such a way that if two means are the same, their
intervals will overlap 95,0% of the time.  You can display the
intervals graphically by selecting Means Plot from the list of
Graphical Options.  In the Multiple Range Tests, these intervals are
used to determine which means are significantly different from which
others.


Multiple Range Tests for Magnesium by Species

--------------------------------------------------------------------------------
Method: 95,0 percent LSD
Species        Count     Mean              Homogeneous Groups
--------------------------------------------------------------------------------
T.majus        3         67,4143           X 
S.oleraceus    3         78,7007           X 
S.media        3         82,588            X 
D.erucoides    3         114,074           X 
C. album       3         480,587            X
--------------------------------------------------------------------------------
Contrast                                   Difference           +/-  Limits
--------------------------------------------------------------------------------
C. album - D.erucoides                    *366,513              91,977            
C. album - S.media                        *397,999              91,977            
C. album - S.oleraceus                    *401,886              91,977            
C. album - T.majus                        *413,173              91,977            
D.erucoides - S.media                      31,4857              91,977            
D.erucoides - S.oleraceus                  35,373               91,977            
D.erucoides - T.majus                      46,6593              91,977            
S.media - S.oleraceus                      3,88733              91,977            
S.media - T.majus                          15,1737              91,977            
S.oleraceus - T.majus                      11,2863              91,977            
--------------------------------------------------------------------------------
* denotes a statistically significant difference.


The StatAdvisor
---------------
   This table applies a multiple comparison procedure to determine
which means are significantly different from which others.  The bottom
half of the output shows the estimated difference between each pair of
means.  An asterisk has been placed next to 4 pairs, indicating that
these pairs show statistically significant differences at the 95,0%
confidence level.  At the top of the page, 2 homogenous groups are
identified using columns of X's.  Within each column, the levels
containing X's form a group of means within which there are no
statistically significant differences.  The method currently being
used to discriminate among the means is Fisher's least significant
difference (LSD) procedure.  With this method, there is a 5,0% risk of
calling each pair of means significantly different when the actual
difference equals 0.  


Variance Check

Cochran's C test: 0,901802   P-Value = 0,000464926
Bartlett's test: 5,55515   P-Value = 0,0064265
Hartley's test: 145,848
Levene's test: 2,4919   P-Value = 0,110137


The StatAdvisor
---------------
   The four statistics displayed in this table test the null
hypothesis that the standard deviations of Magnesium within each of
the 5 levels of Species is the same.  Of particular interest are the
three P-values.  Since the smallest of the P-values is less than 0,05,
there is a statistically significant difference amongst the standard
deviations at the 95,0% confidence level.  This violates one of the
important assumptions underlying the analysis of variance and will
invalidate most of the standard statistical tests.  


Kruskal-Wallis Test for Magnesium by Species

Species             Sample Size         Average Rank
------------------------------------------------------------
C. album            3                   14,0                
D.erucoides         3                   10,6667             
S.media             3                   6,33333             
S.oleraceus         3                   6,33333             
T.majus             3                   2,66667             
------------------------------------------------------------
Test statistic = 11,5667   P-Value = 0,0208821


The StatAdvisor
---------------
   The Kruskal-Wallis test tests the null hypothesis that the medians
of Magnesium within each of the 5 levels of Species are the same.  The
data from all the levels is first combined and ranked from smallest to
largest.  The average rank is then computed for the data at each
level.  Since the P-value is less than 0,05, there is a statistically
significant difference amongst the medians at the 95,0% confidence
level.  To determine which medians are significantly different from
which others, select Box-and-Whisker Plot from the list of Graphical
Options and select the median notch option.


One-Way ANOVA - Potassium by Species

Analysis Summary

Dependent variable: Potassium
Factor: Species

Number of observations: 15
Number of levels: 5


The StatAdvisor
---------------
   This procedure performs a one-way analysis of variance for
Potassium.  It constructs various tests and graphs to compare the mean
values of Potassium for the 5 different levels of Species.  The F-test
in the ANOVA table will test whether there are any significant
differences amongst the means.  If there are, the Multiple Range Tests
will tell you which means are significantly different from which
others.  If you are worried about the presence of outliers, choose the
Kruskal-Wallis Test which compares medians instead of means.  The
various plots will help you judge the practical significance of the
results, as well as allow you to look for possible violations of the
assumptions underlying the analysis of variance.  


Summary Statistics for Potassium

Species             Count               Average             Variance            Standard deviation  
----------------------------------------------------------------------------------------------------
C. album            3                   1250,63             3559,06             59,6579             
D.erucoides         3                   157,687             134,725             11,6071             
S.media             3                   710,112             9945,65             99,7279             
S.oleraceus         3                   714,847             13438,7             115,925             
T.majus             3                   574,696             3756,49             61,2902             
----------------------------------------------------------------------------------------------------
Total               15                  681,595             135469,0            368,061             

Species             Minimum             Maximum             Range               Stnd. skewness      
----------------------------------------------------------------------------------------------------
C. album            1188,17             1307,02             118,85              -0,320956           
D.erucoides         145,376             168,431             23,055              -0,421749           
S.media             604,3               802,369             198,069             -0,424524           
S.oleraceus         583,805             804,024             220,219             -0,999274           
T.majus             533,206             645,094             111,888             1,16694             
----------------------------------------------------------------------------------------------------
Total               145,376             1307,02             1161,64             0,339517            

Species             Stnd. kurtosis      
----------------------------------------------------------------------------------------------------
C. album                                
D.erucoides                             
S.media                                 
S.oleraceus                             
T.majus                                 
----------------------------------------------------------------------------------------------------
Total               -0,34709            


The StatAdvisor
---------------
   This table shows various statistics for Potassium for each of the 5
levels of Species.  The one-way analysis of variance is primarily
intended to compare the means of the different levels, listed here
under the Average column.  Select Means Plot from the list of
Graphical Options to display the means graphically.  

WARNING: There is more than a 3 to 1 difference between the smallest
standard deviation and the largest.  This may cause problems since the
analysis of variance assumes that the standard deviations at all
levels are equal.  Select Variance Check from the list of Tabular
Options to run a formal statistical test for differences among the
sigmas.  You may want to consider transforming the values of Potassium
to remove any dependence of the standard deviation on the mean.  


One-Way ANOVA - Potassium by Species

Analysis Summary

Dependent variable: Potassium
Factor: Species

Number of observations: 15
Number of levels: 5


The StatAdvisor
---------------
   This procedure performs a one-way analysis of variance for
Potassium.  It constructs various tests and graphs to compare the mean
values of Potassium for the 5 different levels of Species.  The F-test
in the ANOVA table will test whether there are any significant
differences amongst the means.  If there are, the Multiple Range Tests
will tell you which means are significantly different from which
others.  If you are worried about the presence of outliers, choose the
Kruskal-Wallis Test which compares medians instead of means.  The
various plots will help you judge the practical significance of the
results, as well as allow you to look for possible violations of the
assumptions underlying the analysis of variance.  


Summary Statistics for Potassium

Species             Count               Average             Variance            Standard deviation  
----------------------------------------------------------------------------------------------------
C. album            3                   1250,63             3559,06             59,6579             
D.erucoides         3                   157,687             134,725             11,6071             
S.media             3                   710,112             9945,65             99,7279             
S.oleraceus         3                   714,847             13438,7             115,925             
T.majus             3                   574,696             3756,49             61,2902             
----------------------------------------------------------------------------------------------------
Total               15                  681,595             135469,0            368,061             

Species             Minimum             Maximum             Range               Stnd. skewness      
----------------------------------------------------------------------------------------------------
C. album            1188,17             1307,02             118,85              -0,320956           
D.erucoides         145,376             168,431             23,055              -0,421749           
S.media             604,3               802,369             198,069             -0,424524           
S.oleraceus         583,805             804,024             220,219             -0,999274           
T.majus             533,206             645,094             111,888             1,16694             
----------------------------------------------------------------------------------------------------
Total               145,376             1307,02             1161,64             0,339517            

Species             Stnd. kurtosis      
----------------------------------------------------------------------------------------------------
C. album                                
D.erucoides                             
S.media                                 
S.oleraceus                             
T.majus                                 
----------------------------------------------------------------------------------------------------
Total               -0,34709            


The StatAdvisor
---------------
   This table shows various statistics for Potassium for each of the 5
levels of Species.  The one-way analysis of variance is primarily
intended to compare the means of the different levels, listed here
under the Average column.  Select Means Plot from the list of
Graphical Options to display the means graphically.  

WARNING: There is more than a 3 to 1 difference between the smallest
standard deviation and the largest.  This may cause problems since the
analysis of variance assumes that the standard deviations at all
levels are equal.  Select Variance Check from the list of Tabular
Options to run a formal statistical test for differences among the
sigmas.  You may want to consider transforming the values of Potassium
to remove any dependence of the standard deviation on the mean.  


ANOVA Table for Potassium by Species

                            Analysis of Variance
-----------------------------------------------------------------------------
Source             Sum of Squares     Df  Mean Square    F-Ratio      P-Value
-----------------------------------------------------------------------------
Between groups           1,8349E6      4     458724,0      74,38       0,0000
Within groups             61669,1     10      6166,91
-----------------------------------------------------------------------------
Total (Corr.)           1,89656E6     14


The StatAdvisor
---------------
   The ANOVA table decomposes the variance of Potassium into two
components: a between-group component and a within-group component. 
The F-ratio, which in this case equals 74,3846, is a ratio of the
between-group estimate to the within-group estimate.  Since the
P-value of the F-test is less than 0,05, there is a statistically
significant difference between the mean Potassium from one level of
Species to another at the 95,0% confidence level.  To determine which
means are significantly different from which others, select Multiple
Range Tests from the list of Tabular Options.


Table of Means for Potassium by Species
with 95,0 percent LSD intervals
--------------------------------------------------------------------------------
                                       Stnd. error
Species          Count         Mean     (pooled s)    Lower limit    Upper limit
--------------------------------------------------------------------------------
C. album             3      1250,63        45,3391         1179,2        1322,07
D.erucoides          3      157,687        45,3391        86,2536         229,12
S.media              3      710,112        45,3391        638,679        781,546
S.oleraceus          3      714,847        45,3391        643,414        786,281
T.majus              3      574,696        45,3391        503,263        646,129
--------------------------------------------------------------------------------
Total               15      681,595


The StatAdvisor
---------------
   This table shows the mean Potassium for each level of Species.  It
also shows the standard error of each mean, which is a measure of its
sampling variability.  The standard error is formed by dividing the
pooled standard deviation by the square root of the number of
observations at each level.  The table also displays an interval
around each mean.  The intervals currently displayed are based on
Fisher's least significant difference (LSD) procedure.  They are
constructed in such a way that if two means are the same, their
intervals will overlap 95,0% of the time.  You can display the
intervals graphically by selecting Means Plot from the list of
Graphical Options.  In the Multiple Range Tests, these intervals are
used to determine which means are significantly different from which
others.


Multiple Range Tests for Potassium by Species

--------------------------------------------------------------------------------
Method: 95,0 percent LSD
Species        Count     Mean              Homogeneous Groups
--------------------------------------------------------------------------------
D.erucoides    3         157,687           X  
T.majus        3         574,696            X 
S.media        3         710,112            X 
S.oleraceus    3         714,847            X 
C. album       3         1250,63             X
--------------------------------------------------------------------------------
Contrast                                   Difference           +/-  Limits
--------------------------------------------------------------------------------
C. album - D.erucoides                    *1092,95              142,867           
C. album - S.media                        *540,522              142,867           
C. album - S.oleraceus                    *535,787              142,867           
C. album - T.majus                        *675,938              142,867           
D.erucoides - S.media                     *-552,425             142,867           
D.erucoides - S.oleraceus                 *-557,16              142,867           
D.erucoides - T.majus                     *-417,009             142,867           
S.media - S.oleraceus                      -4,735               142,867           
S.media - T.majus                          135,416              142,867           
S.oleraceus - T.majus                      140,151              142,867           
--------------------------------------------------------------------------------
* denotes a statistically significant difference.


The StatAdvisor
---------------
   This table applies a multiple comparison procedure to determine
which means are significantly different from which others.  The bottom
half of the output shows the estimated difference between each pair of
means.  An asterisk has been placed next to 7 pairs, indicating that
these pairs show statistically significant differences at the 95,0%
confidence level.  At the top of the page, 3 homogenous groups are
identified using columns of X's.  Within each column, the levels
containing X's form a group of means within which there are no
statistically significant differences.  The method currently being
used to discriminate among the means is Fisher's least significant
difference (LSD) procedure.  With this method, there is a 5,0% risk of
calling each pair of means significantly different when the actual
difference equals 0.  


Variance Check

Cochran's C test: 0,435831   P-Value = 0,506533
Bartlett's test: 2,05948   P-Value = 0,197627
Hartley's test: 99,7489
Levene's test: 0,64368   P-Value = 0,643679


The StatAdvisor
---------------
   The four statistics displayed in this table test the null
hypothesis that the standard deviations of Potassium within each of
the 5 levels of Species is the same.  Of particular interest are the
three P-values.  Since the smallest of the P-values is greater than or
equal to 0,05, there is not a statistically significant difference
amongst the standard deviations at the 95,0% confidence level.  


Kruskal-Wallis Test for Potassium by Species

Species             Sample Size         Average Rank
------------------------------------------------------------
C. album            3                   14,0                
D.erucoides         3                   2,0                 
S.media             3                   9,0                 
S.oleraceus         3                   9,33333             
T.majus             3                   5,66667             
------------------------------------------------------------
Test statistic = 12,0333   P-Value = 0,0171051


The StatAdvisor
---------------
   The Kruskal-Wallis test tests the null hypothesis that the medians
of Potassium within each of the 5 levels of Species are the same.  The
data from all the levels is first combined and ranked from smallest to
largest.  The average rank is then computed for the data at each
level.  Since the P-value is less than 0,05, there is a statistically
significant difference amongst the medians at the 95,0% confidence
level.  To determine which medians are significantly different from
which others, select Box-and-Whisker Plot from the list of Graphical
Options and select the median notch option.


One-Way ANOVA - Phosphorus by Species

Analysis Summary

Dependent variable: Phosphorus
Factor: Species

Number of observations: 15
Number of levels: 5


The StatAdvisor
---------------
   This procedure performs a one-way analysis of variance for
Phosphorus.  It constructs various tests and graphs to compare the
mean values of Phosphorus for the 5 different levels of Species.  The
F-test in the ANOVA table will test whether there are any significant
differences amongst the means.  If there are, the Multiple Range Tests
will tell you which means are significantly different from which
others.  If you are worried about the presence of outliers, choose the
Kruskal-Wallis Test which compares medians instead of means.  The
various plots will help you judge the practical significance of the
results, as well as allow you to look for possible violations of the
assumptions underlying the analysis of variance.  


Summary Statistics for Phosphorus

Species             Count               Average             Variance            Standard deviation  
----------------------------------------------------------------------------------------------------
C. album            3                   81,8067             38,5168             6,20619             
D.erucoides         3                   47,656              46,5461             6,82247             
S.media             3                   44,686              162,698             12,7553             
S.oleraceus         3                   50,1057             37,2891             6,10648             
T.majus             3                   49,3293             9,49076             3,08071             
----------------------------------------------------------------------------------------------------
Total               15                  54,7167             242,353             15,5677             

Species             Minimum             Maximum             Range               Stnd. skewness      
----------------------------------------------------------------------------------------------------
C. album            74,931              86,994              12,063              -0,801565           
D.erucoides         42,515              55,396              12,881              1,03626             
S.media             32,83               58,182              25,352              0,402356            
S.oleraceus         43,061              53,89               10,829              -1,21461            
T.majus             45,78               51,31               5,53                -1,20015            
----------------------------------------------------------------------------------------------------
Total               32,83               86,994              54,164              1,67777             

Species             Stnd. kurtosis      
----------------------------------------------------------------------------------------------------
C. album                                
D.erucoides                             
S.media                                 
S.oleraceus                             
T.majus                                 
----------------------------------------------------------------------------------------------------
Total               0,337048            


The StatAdvisor
---------------
   This table shows various statistics for Phosphorus for each of the
5 levels of Species.  The one-way analysis of variance is primarily
intended to compare the means of the different levels, listed here
under the Average column.  Select Means Plot from the list of
Graphical Options to display the means graphically.  

WARNING: There is more than a 3 to 1 difference between the smallest
standard deviation and the largest.  This may cause problems since the
analysis of variance assumes that the standard deviations at all
levels are equal.  Select Variance Check from the list of Tabular
Options to run a formal statistical test for differences among the
sigmas.  You may want to consider transforming the values of
Phosphorus to remove any dependence of the standard deviation on the
mean.  


ANOVA Table for Phosphorus by Species

                            Analysis of Variance
-----------------------------------------------------------------------------
Source             Sum of Squares     Df  Mean Square    F-Ratio      P-Value
-----------------------------------------------------------------------------
Between groups            2803,86      4      700,965      11,90       0,0008
Within groups             589,082     10      58,9082
-----------------------------------------------------------------------------
Total (Corr.)             3392,94     14


The StatAdvisor
---------------
   The ANOVA table decomposes the variance of Phosphorus into two
components: a between-group component and a within-group component. 
The F-ratio, which in this case equals 11,8993, is a ratio of the
between-group estimate to the within-group estimate.  Since the
P-value of the F-test is less than 0,05, there is a statistically
significant difference between the mean Phosphorus from one level of
Species to another at the 95,0% confidence level.  To determine which
means are significantly different from which others, select Multiple
Range Tests from the list of Tabular Options.


Table of Means for Phosphorus by Species
with 95,0 percent LSD intervals
--------------------------------------------------------------------------------
                                       Stnd. error
Species          Count         Mean     (pooled s)    Lower limit    Upper limit
--------------------------------------------------------------------------------
C. album             3      81,8067        4,43126        74,8251        88,7883
D.erucoides          3       47,656        4,43126        40,6744        54,6376
S.media              3       44,686        4,43126        37,7044        51,6676
S.oleraceus          3      50,1057        4,43126        43,1241        57,0873
T.majus              3      49,3293        4,43126        42,3477        56,3109
--------------------------------------------------------------------------------
Total               15      54,7167


The StatAdvisor
---------------
   This table shows the mean Phosphorus for each level of Species.  It
also shows the standard error of each mean, which is a measure of its
sampling variability.  The standard error is formed by dividing the
pooled standard deviation by the square root of the number of
observations at each level.  The table also displays an interval
around each mean.  The intervals currently displayed are based on
Fisher's least significant difference (LSD) procedure.  They are
constructed in such a way that if two means are the same, their
intervals will overlap 95,0% of the time.  You can display the
intervals graphically by selecting Means Plot from the list of
Graphical Options.  In the Multiple Range Tests, these intervals are
used to determine which means are significantly different from which
others.


Multiple Range Tests for Phosphorus by Species

--------------------------------------------------------------------------------
Method: 95,0 percent LSD
Species        Count     Mean              Homogeneous Groups
--------------------------------------------------------------------------------
S.media        3         44,686            X 
D.erucoides    3         47,656            X 
T.majus        3         49,3293           X 
S.oleraceus    3         50,1057           X 
C. album       3         81,8067            X
--------------------------------------------------------------------------------
Contrast                                   Difference           +/-  Limits
--------------------------------------------------------------------------------
C. album - D.erucoides                    *34,1507              13,9632           
C. album - S.media                        *37,1207              13,9632           
C. album - S.oleraceus                    *31,701               13,9632           
C. album - T.majus                        *32,4773              13,9632           
D.erucoides - S.media                      2,97                 13,9632           
D.erucoides - S.oleraceus                  -2,44967             13,9632           
D.erucoides - T.majus                      -1,67333             13,9632           
S.media - S.oleraceus                      -5,41967             13,9632           
S.media - T.majus                          -4,64333             13,9632           
S.oleraceus - T.majus                      0,776333             13,9632           
--------------------------------------------------------------------------------
* denotes a statistically significant difference.


The StatAdvisor
---------------
   This table applies a multiple comparison procedure to determine
which means are significantly different from which others.  The bottom
half of the output shows the estimated difference between each pair of
means.  An asterisk has been placed next to 4 pairs, indicating that
these pairs show statistically significant differences at the 95,0%
confidence level.  At the top of the page, 2 homogenous groups are
identified using columns of X's.  Within each column, the levels
containing X's form a group of means within which there are no
statistically significant differences.  The method currently being
used to discriminate among the means is Fisher's least significant
difference (LSD) procedure.  With this method, there is a 5,0% risk of
calling each pair of means significantly different when the actual
difference equals 0.  


Variance Check

Cochran's C test: 0,552379   P-Value = 0,200731
Bartlett's test: 1,47033   P-Value = 0,522929
Hartley's test: 17,1428
Levene's test: 0,597037   P-Value = 0,673107


The StatAdvisor
---------------
   The four statistics displayed in this table test the null
hypothesis that the standard deviations of Phosphorus within each of
the 5 levels of Species is the same.  Of particular interest are the
three P-values.  Since the smallest of the P-values is greater than or
equal to 0,05, there is not a statistically significant difference
amongst the standard deviations at the 95,0% confidence level.  


Kruskal-Wallis Test for Phosphorus by Species

Species             Sample Size         Average Rank
------------------------------------------------------------
C. album            3                   14,0                
D.erucoides         3                   6,0                 
S.media             3                   5,33333             
S.oleraceus         3                   7,66667             
T.majus             3                   7,0                 
------------------------------------------------------------
Test statistic = 7,23333   P-Value = 0,12406


The StatAdvisor
---------------
   The Kruskal-Wallis test tests the null hypothesis that the medians
of Phosphorus within each of the 5 levels of Species are the same. 
The data from all the levels is first combined and ranked from
smallest to largest.  The average rank is then computed for the data
at each level.  Since the P-value is greater than or equal to 0,05,
there is not a statistically significant difference amongst the
medians at the 95,0% confidence level.  


One-Way ANOVA - Sodium by Species

Analysis Summary

Dependent variable: Sodium
Factor: Species

Number of observations: 15
Number of levels: 5


The StatAdvisor
---------------
   This procedure performs a one-way analysis of variance for Sodium. 
It constructs various tests and graphs to compare the mean values of
Sodium for the 5 different levels of Species.  The F-test in the ANOVA
table will test whether there are any significant differences amongst
the means.  If there are, the Multiple Range Tests will tell you which
means are significantly different from which others.  If you are
worried about the presence of outliers, choose the Kruskal-Wallis Test
which compares medians instead of means.  The various plots will help
you judge the practical significance of the results, as well as allow
you to look for possible violations of the assumptions underlying the
analysis of variance.  


Summary Statistics for Sodium

Species             Count               Average             Variance            Standard deviation  
----------------------------------------------------------------------------------------------------
C. album            3                   7,50833             0,630012            0,793733            
D.erucoides         3                   14,8177             1,22375             1,10623             
S.media             3                   24,8443             80,5378             8,97428             
S.oleraceus         3                   39,3993             67,111              8,19213             
T.majus             3                   16,0687             0,0795323           0,282015            
----------------------------------------------------------------------------------------------------
Total               15                  20,5277             149,247             12,2167             

Species             Minimum             Maximum             Range               Stnd. skewness      
----------------------------------------------------------------------------------------------------
C. album            6,657               8,228               1,571               -0,513311           
D.erucoides         13,635              15,827              2,192               -0,486338           
S.media             14,55               31,02               16,47               -1,15275            
S.oleraceus         30,466              46,56               16,094              -0,656298           
T.majus             15,797              16,36               0,563               0,22082             
----------------------------------------------------------------------------------------------------
Total               6,657               46,56               39,903              1,53123             

Species             Stnd. kurtosis      
----------------------------------------------------------------------------------------------------
C. album                                
D.erucoides                             
S.media                                 
S.oleraceus                             
T.majus                                 
----------------------------------------------------------------------------------------------------
Total               -0,00988439         


The StatAdvisor
---------------
   This table shows various statistics for Sodium for each of the 5
levels of Species.  The one-way analysis of variance is primarily
intended to compare the means of the different levels, listed here
under the Average column.  Select Means Plot from the list of
Graphical Options to display the means graphically.  

WARNING: There is more than a 3 to 1 difference between the smallest
standard deviation and the largest.  This may cause problems since the
analysis of variance assumes that the standard deviations at all
levels are equal.  Select Variance Check from the list of Tabular
Options to run a formal statistical test for differences among the
sigmas.  You may want to consider transforming the values of Sodium to
remove any dependence of the standard deviation on the mean.  


ANOVA Table for Sodium by Species

                            Analysis of Variance
-----------------------------------------------------------------------------
Source             Sum of Squares     Df  Mean Square    F-Ratio      P-Value
-----------------------------------------------------------------------------
Between groups            1790,29      4      447,572      14,96       0,0003
Within groups             299,164     10      29,9164
-----------------------------------------------------------------------------
Total (Corr.)             2089,45     14


The StatAdvisor
---------------
   The ANOVA table decomposes the variance of Sodium into two
components: a between-group component and a within-group component. 
The F-ratio, which in this case equals 14,9608, is a ratio of the
between-group estimate to the within-group estimate.  Since the
P-value of the F-test is less than 0,05, there is a statistically
significant difference between the mean Sodium from one level of
Species to another at the 95,0% confidence level.  To determine which
means are significantly different from which others, select Multiple
Range Tests from the list of Tabular Options.


Table of Means for Sodium by Species
with 95,0 percent LSD intervals
--------------------------------------------------------------------------------
                                       Stnd. error
Species          Count         Mean     (pooled s)    Lower limit    Upper limit
--------------------------------------------------------------------------------
C. album             3      7,50833        3,15787          2,533        12,4837
D.erucoides          3      14,8177        3,15787        9,84233         19,793
S.media              3      24,8443        3,15787         19,869        29,8197
S.oleraceus          3      39,3993        3,15787         34,424        44,3747
T.majus              3      16,0687        3,15787        11,0933         21,044
--------------------------------------------------------------------------------
Total               15      20,5277


The StatAdvisor
---------------
   This table shows the mean Sodium for each level of Species.  It
also shows the standard error of each mean, which is a measure of its
sampling variability.  The standard error is formed by dividing the
pooled standard deviation by the square root of the number of
observations at each level.  The table also displays an interval
around each mean.  The intervals currently displayed are based on
Fisher's least significant difference (LSD) procedure.  They are
constructed in such a way that if two means are the same, their
intervals will overlap 95,0% of the time.  You can display the
intervals graphically by selecting Means Plot from the list of
Graphical Options.  In the Multiple Range Tests, these intervals are
used to determine which means are significantly different from which
others.


Multiple Range Tests for Sodium by Species

--------------------------------------------------------------------------------
Method: 95,0 percent LSD
Species        Count     Mean              Homogeneous Groups
--------------------------------------------------------------------------------
C. album       3         7,50833           X  
D.erucoides    3         14,8177           X  
T.majus        3         16,0687           XX 
S.media        3         24,8443            X 
S.oleraceus    3         39,3993             X
--------------------------------------------------------------------------------
Contrast                                   Difference           +/-  Limits
--------------------------------------------------------------------------------
C. album - D.erucoides                     -7,30933             9,95067           
C. album - S.media                        *-17,336              9,95067           
C. album - S.oleraceus                    *-31,891              9,95067           
C. album - T.majus                         -8,56033             9,95067           
D.erucoides - S.media                     *-10,0267             9,95067           
D.erucoides - S.oleraceus                 *-24,5817             9,95067           
D.erucoides - T.majus                      -1,251               9,95067           
S.media - S.oleraceus                     *-14,555              9,95067           
S.media - T.majus                          8,77567              9,95067           
S.oleraceus - T.majus                     *23,3307              9,95067           
--------------------------------------------------------------------------------
* denotes a statistically significant difference.


The StatAdvisor
---------------
   This table applies a multiple comparison procedure to determine
which means are significantly different from which others.  The bottom
half of the output shows the estimated difference between each pair of
means.  An asterisk has been placed next to 6 pairs, indicating that
these pairs show statistically significant differences at the 95,0%
confidence level.  At the top of the page, 3 homogenous groups are
identified using columns of X's.  Within each column, the levels
containing X's form a group of means within which there are no
statistically significant differences.  The method currently being
used to discriminate among the means is Fisher's least significant
difference (LSD) procedure.  With this method, there is a 5,0% risk of
calling each pair of means significantly different when the actual
difference equals 0.  


Variance Check

Cochran's C test: 0,538419   P-Value = 0,226967
Bartlett's test: 9,37203   P-Value = 0,000921585
Hartley's test: 1012,64
Levene's test: 1,22777   P-Value = 0,359065


The StatAdvisor
---------------
   The four statistics displayed in this table test the null
hypothesis that the standard deviations of Sodium within each of the 5
levels of Species is the same.  Of particular interest are the three
P-values.  Since the smallest of the P-values is less than 0,05, there
is a statistically significant difference amongst the standard
deviations at the 95,0% confidence level.  This violates one of the
important assumptions underlying the analysis of variance and will
invalidate most of the standard statistical tests.  


Kruskal-Wallis Test for Sodium by Species

Species             Sample Size         Average Rank
------------------------------------------------------------
C. album            3                   2,0                 
D.erucoides         3                   6,0                 
S.media             3                   9,66667             
S.oleraceus         3                   13,6667             
T.majus             3                   8,66667             
------------------------------------------------------------
Test statistic = 11,3   P-Value = 0,0233915


The StatAdvisor
---------------
   The Kruskal-Wallis test tests the null hypothesis that the medians
of Sodium within each of the 5 levels of Species are the same.  The
data from all the levels is first combined and ranked from smallest to
largest.  The average rank is then computed for the data at each
level.  Since the P-value is less than 0,05, there is a statistically
significant difference amongst the medians at the 95,0% confidence
level.  To determine which medians are significantly different from
which others, select Box-and-Whisker Plot from the list of Graphical
Options and select the median notch option.


One-Way ANOVA - Iron by Species

Analysis Summary

Dependent variable: Iron
Factor: Species

Number of observations: 15
Number of levels: 5


The StatAdvisor
---------------
   This procedure performs a one-way analysis of variance for Iron. 
It constructs various tests and graphs to compare the mean values of
Iron for the 5 different levels of Species.  The F-test in the ANOVA
table will test whether there are any significant differences amongst
the means.  If there are, the Multiple Range Tests will tell you which
means are significantly different from which others.  If you are
worried about the presence of outliers, choose the Kruskal-Wallis Test
which compares medians instead of means.  The various plots will help
you judge the practical significance of the results, as well as allow
you to look for possible violations of the assumptions underlying the
analysis of variance.  


Summary Statistics for Iron

Species             Count               Average             Variance            Standard deviation  
----------------------------------------------------------------------------------------------------
C. album            3                   1,97133             0,0493223           0,222086            
D.erucoides         3                   1,16933             0,0198803           0,140998            
S.media             3                   1,29567             0,190629            0,436611            
S.oleraceus         3                   1,541               0,092241            0,303712            
T.majus             3                   0,575667            0,000954333         0,0308923           
----------------------------------------------------------------------------------------------------
Total               15                  1,3106              0,275424            0,524808            

Species             Minimum             Maximum             Range               Stnd. skewness      
----------------------------------------------------------------------------------------------------
C. album            1,763               2,205               0,442               0,358245            
D.erucoides         1,039               1,319               0,28                0,428104            
S.media             0,873               1,745               0,872               0,193619            
S.oleraceus         1,205               1,796               0,591               -0,788272           
T.majus             0,54                0,594               0,054               -1,2233             
----------------------------------------------------------------------------------------------------
Total               0,54                2,205               1,665               -0,0119031          

Species             Stnd. kurtosis      
----------------------------------------------------------------------------------------------------
C. album                                
D.erucoides                             
S.media                                 
S.oleraceus                             
T.majus                                 
----------------------------------------------------------------------------------------------------
Total               -0,838541           


The StatAdvisor
---------------
   This table shows various statistics for Iron for each of the 5
levels of Species.  The one-way analysis of variance is primarily
intended to compare the means of the different levels, listed here
under the Average column.  Select Means Plot from the list of
Graphical Options to display the means graphically.  

WARNING: There is more than a 3 to 1 difference between the smallest
standard deviation and the largest.  This may cause problems since the
analysis of variance assumes that the standard deviations at all
levels are equal.  Select Variance Check from the list of Tabular
Options to run a formal statistical test for differences among the
sigmas.  You may want to consider transforming the values of Iron to
remove any dependence of the standard deviation on the mean.  


ANOVA Table for Iron by Species

                            Analysis of Variance
-----------------------------------------------------------------------------
Source             Sum of Squares     Df  Mean Square    F-Ratio      P-Value
-----------------------------------------------------------------------------
Between groups            3,14988      4     0,787469      11,15       0,0010
Within groups            0,706055     10    0,0706055
-----------------------------------------------------------------------------
Total (Corr.)             3,85593     14


The StatAdvisor
---------------
   The ANOVA table decomposes the variance of Iron into two
components: a between-group component and a within-group component. 
The F-ratio, which in this case equals 11,1531, is a ratio of the
between-group estimate to the within-group estimate.  Since the
P-value of the F-test is less than 0,05, there is a statistically
significant difference between the mean Iron from one level of Species
to another at the 95,0% confidence level.  To determine which means
are significantly different from which others, select Multiple Range
Tests from the list of Tabular Options.


Table of Means for Iron by Species
with 95,0 percent LSD intervals
--------------------------------------------------------------------------------
                                       Stnd. error
Species          Count         Mean     (pooled s)    Lower limit    Upper limit
--------------------------------------------------------------------------------
C. album             3      1,97133       0,153412        1,72963        2,21304
D.erucoides          3      1,16933       0,153412       0,927628        1,41104
S.media              3      1,29567       0,153412        1,05396        1,53737
S.oleraceus          3        1,541       0,153412        1,29929        1,78271
T.majus              3     0,575667       0,153412       0,333961       0,817372
--------------------------------------------------------------------------------
Total               15       1,3106


The StatAdvisor
---------------
   This table shows the mean Iron for each level of Species.  It also
shows the standard error of each mean, which is a measure of its
sampling variability.  The standard error is formed by dividing the
pooled standard deviation by the square root of the number of
observations at each level.  The table also displays an interval
around each mean.  The intervals currently displayed are based on
Fisher's least significant difference (LSD) procedure.  They are
constructed in such a way that if two means are the same, their
intervals will overlap 95,0% of the time.  You can display the
intervals graphically by selecting Means Plot from the list of
Graphical Options.  In the Multiple Range Tests, these intervals are
used to determine which means are significantly different from which
others.


Multiple Range Tests for Iron by Species

--------------------------------------------------------------------------------
Method: 95,0 percent LSD
Species        Count     Mean              Homogeneous Groups
--------------------------------------------------------------------------------
T.majus        3         0,575667          X  
D.erucoides    3         1,16933            X 
S.media        3         1,29567            X 
S.oleraceus    3         1,541              XX
C. album       3         1,97133             X
--------------------------------------------------------------------------------
Contrast                                   Difference           +/-  Limits
--------------------------------------------------------------------------------
C. album - D.erucoides                    *0,802                0,483411          
C. album - S.media                        *0,675667             0,483411          
C. album - S.oleraceus                     0,430333             0,483411          
C. album - T.majus                        *1,39567              0,483411          
D.erucoides - S.media                      -0,126333            0,483411          
D.erucoides - S.oleraceus                  -0,371667            0,483411          
D.erucoides - T.majus                     *0,593667             0,483411          
S.media - S.oleraceus                      -0,245333            0,483411          
S.media - T.majus                         *0,72                 0,483411          
S.oleraceus - T.majus                     *0,965333             0,483411          
--------------------------------------------------------------------------------
* denotes a statistically significant difference.


The StatAdvisor
---------------
   This table applies a multiple comparison procedure to determine
which means are significantly different from which others.  The bottom
half of the output shows the estimated difference between each pair of
means.  An asterisk has been placed next to 6 pairs, indicating that
these pairs show statistically significant differences at the 95,0%
confidence level.  At the top of the page, 3 homogenous groups are
identified using columns of X's.  Within each column, the levels
containing X's form a group of means within which there are no
statistically significant differences.  The method currently being
used to discriminate among the means is Fisher's least significant
difference (LSD) procedure.  With this method, there is a 5,0% risk of
calling each pair of means significantly different when the actual
difference equals 0.  


Variance Check

Cochran's C test: 0,539985   P-Value = 0,223903
Bartlett's test: 2,54438   P-Value = 0,0998826
Hartley's test: 199,751
Levene's test: 1,18562   P-Value = 0,374565


The StatAdvisor
---------------
   The four statistics displayed in this table test the null
hypothesis that the standard deviations of Iron within each of the 5
levels of Species is the same.  Of particular interest are the three
P-values.  Since the smallest of the P-values is greater than or equal
to 0,05, there is not a statistically significant difference amongst
the standard deviations at the 95,0% confidence level.  


Kruskal-Wallis Test for Iron by Species

Species             Sample Size         Average Rank
------------------------------------------------------------
C. album            3                   13,6667             
D.erucoides         3                   6,66667             
S.media             3                   7,66667             
S.oleraceus         3                   10,0                
T.majus             3                   2,0                 
------------------------------------------------------------
Test statistic = 11,1   P-Value = 0,0254628


The StatAdvisor
---------------
   The Kruskal-Wallis test tests the null hypothesis that the medians
of Iron within each of the 5 levels of Species are the same.  The data
from all the levels is first combined and ranked from smallest to
largest.  The average rank is then computed for the data at each
level.  Since the P-value is less than 0,05, there is a statistically
significant difference amongst the medians at the 95,0% confidence
level.  To determine which medians are significantly different from
which others, select Box-and-Whisker Plot from the list of Graphical
Options and select the median notch option.


One-Way ANOVA - Copper by Species

Analysis Summary

Dependent variable: Copper
Factor: Species

Number of observations: 15
Number of levels: 5


The StatAdvisor
---------------
   This procedure performs a one-way analysis of variance for Copper. 
It constructs various tests and graphs to compare the mean values of
Copper for the 5 different levels of Species.  The F-test in the ANOVA
table will test whether there are any significant differences amongst
the means.  If there are, the Multiple Range Tests will tell you which
means are significantly different from which others.  If you are
worried about the presence of outliers, choose the Kruskal-Wallis Test
which compares medians instead of means.  The various plots will help
you judge the practical significance of the results, as well as allow
you to look for possible violations of the assumptions underlying the
analysis of variance.  


Summary Statistics for Copper

Species             Count               Average             Variance            Standard deviation  
----------------------------------------------------------------------------------------------------
C. album            3                   0,191               0,008427            0,0917987           
D.erucoides         3                   0,113               0,002884            0,0537029           
S.media             3                   0,076               0,000237            0,0153948           
S.oleraceus         3                   0,132333            0,00113633          0,0337095           
T.majus             3                   0,0823333           0,00243333          0,0493288           
----------------------------------------------------------------------------------------------------
Total               15                  0,118933            0,00400064          0,0632506           

Species             Minimum             Maximum             Range               Stnd. skewness      
----------------------------------------------------------------------------------------------------
C. album            0,138               0,297               0,159               1,22474             
D.erucoides         0,081               0,175               0,094               1,22283             
S.media             0,059               0,089               0,03                -0,770952           
S.oleraceus         0,101               0,168               0,067               0,402282            
T.majus             0,049               0,139               0,09                1,16836             
----------------------------------------------------------------------------------------------------
Total               0,049               0,297               0,248               2,5739              

Species             Stnd. kurtosis      
----------------------------------------------------------------------------------------------------
C. album                                
D.erucoides                             
S.media                                 
S.oleraceus                             
T.majus                                 
----------------------------------------------------------------------------------------------------
Total               2,8724              


The StatAdvisor
---------------
   This table shows various statistics for Copper for each of the 5
levels of Species.  The one-way analysis of variance is primarily
intended to compare the means of the different levels, listed here
under the Average column.  Select Means Plot from the list of
Graphical Options to display the means graphically.  

WARNING: There is more than a 3 to 1 difference between the smallest
standard deviation and the largest.  This may cause problems since the
analysis of variance assumes that the standard deviations at all
levels are equal.  Select Variance Check from the list of Tabular
Options to run a formal statistical test for differences among the
sigmas.  You may want to consider transforming the values of Copper to
remove any dependence of the standard deviation on the mean.  


ANOVA Table for Copper by Species

                            Analysis of Variance
-----------------------------------------------------------------------------
Source             Sum of Squares     Df  Mean Square    F-Ratio      P-Value
-----------------------------------------------------------------------------
Between groups          0,0257736      4    0,0064434       2,13       0,1513
Within groups           0,0302353     10   0,00302353
-----------------------------------------------------------------------------
Total (Corr.)           0,0560089     14


The StatAdvisor
---------------
   The ANOVA table decomposes the variance of Copper into two
components: a between-group component and a within-group component. 
The F-ratio, which in this case equals 2,13108, is a ratio of the
between-group estimate to the within-group estimate.  Since the
P-value of the F-test is greater than or equal to 0,05, there is not a
statistically significant difference between the mean Copper from one
level of Species to another at the 95,0% confidence level.


Table of Means for Copper by Species
with 95,0 percent LSD intervals
--------------------------------------------------------------------------------
                                       Stnd. error
Species          Count         Mean     (pooled s)    Lower limit    Upper limit
--------------------------------------------------------------------------------
C. album             3        0,191      0,0317466       0,140982       0,241018
D.erucoides          3        0,113      0,0317466      0,0629822       0,163018
S.media              3        0,076      0,0317466      0,0259822       0,126018
S.oleraceus          3     0,132333      0,0317466      0,0823155       0,182351
T.majus              3    0,0823333      0,0317466      0,0323155       0,132351
--------------------------------------------------------------------------------
Total               15     0,118933


The StatAdvisor
---------------
   This table shows the mean Copper for each level of Species.  It
also shows the standard error of each mean, which is a measure of its
sampling variability.  The standard error is formed by dividing the
pooled standard deviation by the square root of the number of
observations at each level.  The table also displays an interval
around each mean.  The intervals currently displayed are based on
Fisher's least significant difference (LSD) procedure.  They are
constructed in such a way that if two means are the same, their
intervals will overlap 95,0% of the time.  You can display the
intervals graphically by selecting Means Plot from the list of
Graphical Options.  In the Multiple Range Tests, these intervals are
used to determine which means are significantly different from which
others.


Multiple Range Tests for Copper by Species

--------------------------------------------------------------------------------
Method: 95,0 percent LSD
Species        Count     Mean              Homogeneous Groups
--------------------------------------------------------------------------------
S.media        3         0,076             X 
T.majus        3         0,0823333         X 
D.erucoides    3         0,113             XX
S.oleraceus    3         0,132333          XX
C. album       3         0,191              X
--------------------------------------------------------------------------------
Contrast                                   Difference           +/-  Limits
--------------------------------------------------------------------------------
C. album - D.erucoides                     0,078                0,100036          
C. album - S.media                        *0,115                0,100036          
C. album - S.oleraceus                     0,0586667            0,100036          
C. album - T.majus                        *0,108667             0,100036          
D.erucoides - S.media                      0,037                0,100036          
D.erucoides - S.oleraceus                  -0,0193333           0,100036          
D.erucoides - T.majus                      0,0306667            0,100036          
S.media - S.oleraceus                      -0,0563333           0,100036          
S.media - T.majus                          -0,00633333          0,100036          
S.oleraceus - T.majus                      0,05                 0,100036          
--------------------------------------------------------------------------------
* denotes a statistically significant difference.


The StatAdvisor
---------------
   This table applies a multiple comparison procedure to determine
which means are significantly different from which others.  The bottom
half of the output shows the estimated difference between each pair of
means.  An asterisk has been placed next to 2 pairs, indicating that
these pairs show statistically significant differences at the 95,0%
confidence level.  At the top of the page, 2 homogenous groups are
identified using columns of X's.  Within each column, the levels
containing X's form a group of means within which there are no
statistically significant differences.  The method currently being
used to discriminate among the means is Fisher's least significant
difference (LSD) procedure.  With this method, there is a 5,0% risk of
calling each pair of means significantly different when the actual
difference equals 0.  


Variance Check

Cochran's C test: 0,557427   P-Value = 0,191826
Bartlett's test: 1,73816   P-Value = 0,33006
Hartley's test: 35,557
Levene's test: 0,271929   P-Value = 0,889453


The StatAdvisor
---------------
   The four statistics displayed in this table test the null
hypothesis that the standard deviations of Copper within each of the 5
levels of Species is the same.  Of particular interest are the three
P-values.  Since the smallest of the P-values is greater than or equal
to 0,05, there is not a statistically significant difference amongst
the standard deviations at the 95,0% confidence level.  


Kruskal-Wallis Test for Copper by Species

Species             Sample Size         Average Rank
------------------------------------------------------------
C. album            3                   12,0                
D.erucoides         3                   8,33333             
S.media             3                   4,5                 
S.oleraceus         3                   10,0                
T.majus             3                   5,16667             
------------------------------------------------------------
Test statistic = 6,08005   P-Value = 0,193249


The StatAdvisor
---------------
   The Kruskal-Wallis test tests the null hypothesis that the medians
of Copper within each of the 5 levels of Species are the same.  The
data from all the levels is first combined and ranked from smallest to
largest.  The average rank is then computed for the data at each
level.  Since the P-value is greater than or equal to 0,05, there is
not a statistically significant difference amongst the medians at the
95,0% confidence level.  


One-Way ANOVA - Zinc by Species

Analysis Summary

Dependent variable: Zinc
Factor: Species

Number of observations: 15
Number of levels: 5


The StatAdvisor
---------------
   This procedure performs a one-way analysis of variance for Zinc. 
It constructs various tests and graphs to compare the mean values of
Zinc for the 5 different levels of Species.  The F-test in the ANOVA
table will test whether there are any significant differences amongst
the means.  If there are, the Multiple Range Tests will tell you which
means are significantly different from which others.  If you are
worried about the presence of outliers, choose the Kruskal-Wallis Test
which compares medians instead of means.  The various plots will help
you judge the practical significance of the results, as well as allow
you to look for possible violations of the assumptions underlying the
analysis of variance.  


Summary Statistics for Zinc

Species             Count               Average             Variance            Standard deviation  
----------------------------------------------------------------------------------------------------
C. album            3                   0,789               0,017143            0,130931            
D.erucoides         3                   0,468               0,006175            0,0785812           
S.media             3                   0,673333            0,0474203           0,217762            
S.oleraceus         3                   0,764               0,009697            0,0984733           
T.majus             3                   0,726333            0,00844233          0,0918822           
----------------------------------------------------------------------------------------------------
Total               15                  0,684133            0,0268368           0,16382             

Species             Minimum             Maximum             Range               Stnd. skewness      
----------------------------------------------------------------------------------------------------
C. album            0,65                0,91                0,26                -0,42918            
D.erucoides         0,383               0,538               0,155               -0,585262           
S.media             0,462               0,897               0,435               0,179639            
S.oleraceus         0,652               0,837               0,185               -1,06254            
T.majus             0,63                0,813               0,183               -0,331062           
----------------------------------------------------------------------------------------------------
Total               0,383               0,91                0,527               -0,569552           

Species             Stnd. kurtosis      
----------------------------------------------------------------------------------------------------
C. album                                
D.erucoides                             
S.media                                 
S.oleraceus                             
T.majus                                 
----------------------------------------------------------------------------------------------------
Total               -0,727273           


The StatAdvisor
---------------
   This table shows various statistics for Zinc for each of the 5
levels of Species.  The one-way analysis of variance is primarily
intended to compare the means of the different levels, listed here
under the Average column.  Select Means Plot from the list of
Graphical Options to display the means graphically.  


ANOVA Table for Zinc by Species

                            Analysis of Variance
-----------------------------------------------------------------------------
Source             Sum of Squares     Df  Mean Square    F-Ratio      P-Value
-----------------------------------------------------------------------------
Between groups            0,19796      4    0,0494901       2,78       0,0861
Within groups            0,177755     10    0,0177755
-----------------------------------------------------------------------------
Total (Corr.)            0,375716     14


The StatAdvisor
---------------
   The ANOVA table decomposes the variance of Zinc into two
components: a between-group component and a within-group component. 
The F-ratio, which in this case equals 2,78417, is a ratio of the
between-group estimate to the within-group estimate.  Since the
P-value of the F-test is greater than or equal to 0,05, there is not a
statistically significant difference between the mean Zinc from one
level of Species to another at the 95,0% confidence level.


Table of Means for Zinc by Species
with 95,0 percent LSD intervals
--------------------------------------------------------------------------------
                                       Stnd. error
Species          Count         Mean     (pooled s)    Lower limit    Upper limit
--------------------------------------------------------------------------------
C. album             3        0,789      0,0769752       0,667723       0,910277
D.erucoides          3        0,468      0,0769752       0,346723       0,589277
S.media              3     0,673333      0,0769752       0,552056        0,79461
S.oleraceus          3        0,764      0,0769752       0,642723       0,885277
T.majus              3     0,726333      0,0769752       0,605056        0,84761
--------------------------------------------------------------------------------
Total               15     0,684133


The StatAdvisor
---------------
   This table shows the mean Zinc for each level of Species.  It also
shows the standard error of each mean, which is a measure of its
sampling variability.  The standard error is formed by dividing the
pooled standard deviation by the square root of the number of
observations at each level.  The table also displays an interval
around each mean.  The intervals currently displayed are based on
Fisher's least significant difference (LSD) procedure.  They are
constructed in such a way that if two means are the same, their
intervals will overlap 95,0% of the time.  You can display the
intervals graphically by selecting Means Plot from the list of
Graphical Options.  In the Multiple Range Tests, these intervals are
used to determine which means are significantly different from which
others.


Multiple Range Tests for Zinc by Species

--------------------------------------------------------------------------------
Method: 95,0 percent LSD
Species        Count     Mean              Homogeneous Groups
--------------------------------------------------------------------------------
D.erucoides    3         0,468             X 
S.media        3         0,673333          XX
T.majus        3         0,726333           X
S.oleraceus    3         0,764              X
C. album       3         0,789              X
--------------------------------------------------------------------------------
Contrast                                   Difference           +/-  Limits
--------------------------------------------------------------------------------
C. album - D.erucoides                    *0,321                0,242554          
C. album - S.media                         0,115667             0,242554          
C. album - S.oleraceus                     0,025                0,242554          
C. album - T.majus                         0,0626667            0,242554          
D.erucoides - S.media                      -0,205333            0,242554          
D.erucoides - S.oleraceus                 *-0,296               0,242554          
D.erucoides - T.majus                     *-0,258333            0,242554          
S.media - S.oleraceus                      -0,0906667           0,242554          
S.media - T.majus                          -0,053               0,242554          
S.oleraceus - T.majus                      0,0376667            0,242554          
--------------------------------------------------------------------------------
* denotes a statistically significant difference.


The StatAdvisor
---------------
   This table applies a multiple comparison procedure to determine
which means are significantly different from which others.  The bottom
half of the output shows the estimated difference between each pair of
means.  An asterisk has been placed next to 3 pairs, indicating that
these pairs show statistically significant differences at the 95,0%
confidence level.  At the top of the page, 2 homogenous groups are
identified using columns of X's.  Within each column, the levels
containing X's form a group of means within which there are no
statistically significant differences.  The method currently being
used to discriminate among the means is Fisher's least significant
difference (LSD) procedure.  With this method, there is a 5,0% risk of
calling each pair of means significantly different when the actual
difference equals 0.  


Variance Check

Cochran's C test: 0,533546   P-Value = 0,236704
Bartlett's test: 1,33988   P-Value = 0,655744
Hartley's test: 7,67941
Levene's test: 0,630762   P-Value = 0,651742


The StatAdvisor
---------------
   The four statistics displayed in this table test the null
hypothesis that the standard deviations of Zinc within each of the 5
levels of Species is the same.  Of particular interest are the three
P-values.  Since the smallest of the P-values is greater than or equal
to 0,05, there is not a statistically significant difference amongst
the standard deviations at the 95,0% confidence level.  


Kruskal-Wallis Test for Zinc by Species

Species             Sample Size         Average Rank
------------------------------------------------------------
C. album            3                   10,6667             
D.erucoides         3                   2,66667             
S.media             3                   8,0                 
S.oleraceus         3                   10,0                
T.majus             3                   8,66667             
------------------------------------------------------------
Test statistic = 6,0   P-Value = 0,199148


The StatAdvisor
---------------
   The Kruskal-Wallis test tests the null hypothesis that the medians
of Zinc within each of the 5 levels of Species are the same.  The data
from all the levels is first combined and ranked from smallest to
largest.  The average rank is then computed for the data at each
level.  Since the P-value is greater than or equal to 0,05, there is
not a statistically significant difference amongst the medians at the
95,0% confidence level.  


One-Way ANOVA - Nitrates _mg NO3_·kg_1fw_ by Species

Analysis Summary

Dependent variable: Nitrates _mg NO3_·kg_1fw_
Factor: Species

Number of observations: 15
Number of levels: 5


The StatAdvisor
---------------
   This procedure performs a one-way analysis of variance for Nitrates
_mg NO3_·kg_1fw_.  It constructs various tests and graphs to compare
the mean values of Nitrates _mg NO3_·kg_1fw_ for the 5 different
levels of Species.  The F-test in the ANOVA table will test whether
there are any significant differences amongst the means.  If there
are, the Multiple Range Tests will tell you which means are
significantly different from which others.  If you are worried about
the presence of outliers, choose the Kruskal-Wallis Test which
compares medians instead of means.  The various plots will help you
judge the practical significance of the results, as well as allow you
to look for possible violations of the assumptions underlying the
analysis of variance.  


Summary Statistics for Nitrates _mg NO3_·kg_1fw_

Species             Count               Average             Variance            Standard deviation  
----------------------------------------------------------------------------------------------------
C. album            3                   31,6093             39,6052             6,29327             
D.erucoides         3                   17,9573             8,20506             2,86445             
S.media             3                   75,6183             36,0594             6,00495             
S.oleraceus         3                   92,8187             105,371             10,265              
T.majus             3                   56,326              27,1138             5,20709             
----------------------------------------------------------------------------------------------------
Total               15                  54,8659             840,119             28,9848             

Species             Minimum             Maximum             Range               Stnd. skewness      
----------------------------------------------------------------------------------------------------
C. album            25,865              38,336              12,471              0,484582            
D.erucoides         14,657              19,797              5,14                -1,20067            
S.media             70,239              82,097              11,858              0,563005            
S.oleraceus         83,64               103,903             20,263              0,570364            
T.majus             50,676              60,932              10,256              -0,612328           
----------------------------------------------------------------------------------------------------
Total               14,657              103,903             89,246              0,130217            

Species             Stnd. kurtosis      
----------------------------------------------------------------------------------------------------
C. album                                
D.erucoides                             
S.media                                 
S.oleraceus                             
T.majus                                 
----------------------------------------------------------------------------------------------------
Total               -1,04177            


The StatAdvisor
---------------
   This table shows various statistics for Nitrates _mg NO3_·kg_1fw_
for each of the 5 levels of Species.  The one-way analysis of variance
is primarily intended to compare the means of the different levels,
listed here under the Average column.  Select Means Plot from the list
of Graphical Options to display the means graphically.  

WARNING: There is more than a 3 to 1 difference between the smallest
standard deviation and the largest.  This may cause problems since the
analysis of variance assumes that the standard deviations at all
levels are equal.  Select Variance Check from the list of Tabular
Options to run a formal statistical test for differences among the
sigmas.  You may want to consider transforming the values of Nitrates
_mg NO3_·kg_1fw_ to remove any dependence of the standard deviation on
the mean.  


ANOVA Table for Nitrates _mg NO3_·kg_1fw_ by Species

                            Analysis of Variance
-----------------------------------------------------------------------------
Source             Sum of Squares     Df  Mean Square    F-Ratio      P-Value
-----------------------------------------------------------------------------
Between groups            11329,0      4      2832,24      65,45       0,0000
Within groups             432,709     10      43,2709
-----------------------------------------------------------------------------
Total (Corr.)             11761,7     14


The StatAdvisor
---------------
   The ANOVA table decomposes the variance of Nitrates _mg
NO3_·kg_1fw_ into two components: a between-group component and a
within-group component.  The F-ratio, which in this case equals
65,4537, is a ratio of the between-group estimate to the within-group
estimate.  Since the P-value of the F-test is less than 0,05, there is
a statistically significant difference between the mean Nitrates _mg
NO3_·kg_1fw_ from one level of Species to another at the 95,0%
confidence level.  To determine which means are significantly
different from which others, select Multiple Range Tests from the list
of Tabular Options.


Table of Means for Nitrates _mg NO3_·kg_1fw_ by Species
with 95,0 percent LSD intervals
--------------------------------------------------------------------------------
                                       Stnd. error
Species          Count         Mean     (pooled s)    Lower limit    Upper limit
--------------------------------------------------------------------------------
C. album             3      31,6093        3,79785        25,6257         37,593
D.erucoides          3      17,9573        3,79785        11,9737         23,941
S.media              3      75,6183        3,79785        69,6347         81,602
S.oleraceus          3      92,8187        3,79785         86,835        98,8023
T.majus              3       56,326        3,79785        50,3424        62,3096
--------------------------------------------------------------------------------
Total               15      54,8659


The StatAdvisor
---------------
   This table shows the mean Nitrates _mg NO3_·kg_1fw_ for each level
of Species.  It also shows the standard error of each mean, which is a
measure of its sampling variability.  The standard error is formed by
dividing the pooled standard deviation by the square root of the
number of observations at each level.  The table also displays an
interval around each mean.  The intervals currently displayed are
based on Fisher's least significant difference (LSD) procedure.  They
are constructed in such a way that if two means are the same, their
intervals will overlap 95,0% of the time.  You can display the
intervals graphically by selecting Means Plot from the list of
Graphical Options.  In the Multiple Range Tests, these intervals are
used to determine which means are significantly different from which
others.


Multiple Range Tests for Nitrates _mg NO3_·kg_1fw_ by Species

--------------------------------------------------------------------------------
Method: 95,0 percent LSD
Species        Count     Mean              Homogeneous Groups
--------------------------------------------------------------------------------
D.erucoides    3         17,9573           X    
C. album       3         31,6093            X   
T.majus        3         56,326              X  
S.media        3         75,6183              X 
S.oleraceus    3         92,8187               X
--------------------------------------------------------------------------------
Contrast                                   Difference           +/-  Limits
--------------------------------------------------------------------------------
C. album - D.erucoides                    *13,652               11,9673           
C. album - S.media                        *-44,009              11,9673           
C. album - S.oleraceus                    *-61,2093             11,9673           
C. album - T.majus                        *-24,7167             11,9673           
D.erucoides - S.media                     *-57,661              11,9673           
D.erucoides - S.oleraceus                 *-74,8613             11,9673           
D.erucoides - T.majus                     *-38,3687             11,9673           
S.media - S.oleraceus                     *-17,2003             11,9673           
S.media - T.majus                         *19,2923              11,9673           
S.oleraceus - T.majus                     *36,4927              11,9673           
--------------------------------------------------------------------------------
* denotes a statistically significant difference.


The StatAdvisor
---------------
   This table applies a multiple comparison procedure to determine
which means are significantly different from which others.  The bottom
half of the output shows the estimated difference between each pair of
means.  An asterisk has been placed next to 10 pairs, indicating that
these pairs show statistically significant differences at the 95,0%
confidence level.  At the top of the page, 5 homogenous groups are
identified using columns of X's.  Within each column, the levels
containing X's form a group of means within which there are no
statistically significant differences.  The method currently being
used to discriminate among the means is Fisher's least significant
difference (LSD) procedure.  With this method, there is a 5,0% risk of
calling each pair of means significantly different when the actual
difference equals 0.  


Variance Check

Cochran's C test: 0,487029   P-Value = 0,346211
Bartlett's test: 1,35283   P-Value = 0,641359
Hartley's test: 12,8422
Levene's test: 0,5481   P-Value = 0,704852


The StatAdvisor
---------------
   The four statistics displayed in this table test the null
hypothesis that the standard deviations of Nitrates _mg NO3_·kg_1fw_
within each of the 5 levels of Species is the same.  Of particular
interest are the three P-values.  Since the smallest of the P-values
is greater than or equal to 0,05, there is not a statistically
significant difference amongst the standard deviations at the 95,0%
confidence level.  


Kruskal-Wallis Test for Nitrates _mg NO3_·kg_1fw_ by Species

Species             Sample Size         Average Rank
------------------------------------------------------------
C. album            3                   5,0                 
D.erucoides         3                   2,0                 
S.media             3                   11,0                
S.oleraceus         3                   14,0                
T.majus             3                   8,0                 
------------------------------------------------------------
Test statistic = 13,5   P-Value = 0,00907432


The StatAdvisor
---------------
   The Kruskal-Wallis test tests the null hypothesis that the medians
of Nitrates _mg NO3_·kg_1fw_ within each of the 5 levels of Species
are the same.  The data from all the levels is first combined and
ranked from smallest to largest.  The average rank is then computed
for the data at each level.  Since the P-value is less than 0,05,
there is a statistically significant difference amongst the medians at
the 95,0% confidence level.  To determine which medians are
significantly different from which others, select Box-and-Whisker Plot
from the list of Graphical Options and select the median notch option.


One-Way ANOVA - pH by Species

Analysis Summary

Dependent variable: pH
Factor: Species

Number of observations: 15
Number of levels: 5


The StatAdvisor
---------------
   This procedure performs a one-way analysis of variance for pH.  It
constructs various tests and graphs to compare the mean values of pH
for the 5 different levels of Species.  The F-test in the ANOVA table
will test whether there are any significant differences amongst the
means.  If there are, the Multiple Range Tests will tell you which
means are significantly different from which others.  If you are
worried about the presence of outliers, choose the Kruskal-Wallis Test
which compares medians instead of means.  The various plots will help
you judge the practical significance of the results, as well as allow
you to look for possible violations of the assumptions underlying the
analysis of variance.  


Summary Statistics for pH

Species             Count               Average             Variance            Standard deviation  
----------------------------------------------------------------------------------------------------
C. album            3                   6,54                0,0217              0,147309            
D.erucoides         3                   5,72333             0,00243333          0,0493288           
S.media             3                   6,01667             0,0142333           0,119304            
S.oleraceus         3                   6,20667             0,00413333          0,064291            
T.majus             3                   6,00333             0,0386333           0,196554            
----------------------------------------------------------------------------------------------------
Total               15                  6,098               0,0894029           0,299003            

Species             Minimum             Maximum             Range               Stnd. skewness      
----------------------------------------------------------------------------------------------------
C. album            6,45                6,71                0,26                1,2184              
D.erucoides         5,69                5,78                0,09                1,16836             
S.media             5,92                6,15                0,23                0,885573            
S.oleraceus         6,16                6,28                0,12                1,09276             
T.majus             5,78                6,15                0,37                -1,05232            
----------------------------------------------------------------------------------------------------
Total               5,69                6,71                1,02                0,608417            

Species             Stnd. kurtosis      
----------------------------------------------------------------------------------------------------
C. album                                
D.erucoides                             
S.media                                 
S.oleraceus                             
T.majus                                 
----------------------------------------------------------------------------------------------------
Total               -0,279594           


The StatAdvisor
---------------
   This table shows various statistics for pH for each of the 5 levels
of Species.  The one-way analysis of variance is primarily intended to
compare the means of the different levels, listed here under the
Average column.  Select Means Plot from the list of Graphical Options
to display the means graphically.  

WARNING: There is more than a 3 to 1 difference between the smallest
standard deviation and the largest.  This may cause problems since the
analysis of variance assumes that the standard deviations at all
levels are equal.  Select Variance Check from the list of Tabular
Options to run a formal statistical test for differences among the
sigmas.  You may want to consider transforming the values of pH to
remove any dependence of the standard deviation on the mean.  


ANOVA Table for pH by Species

                            Analysis of Variance
-----------------------------------------------------------------------------
Source             Sum of Squares     Df  Mean Square    F-Ratio      P-Value
-----------------------------------------------------------------------------
Between groups            1,08937      4     0,272343      16,78       0,0002
Within groups            0,162267     10    0,0162267
-----------------------------------------------------------------------------
Total (Corr.)             1,25164     14


The StatAdvisor
---------------
   The ANOVA table decomposes the variance of pH into two components:
a between-group component and a within-group component.  The F-ratio,
which in this case equals 16,7837, is a ratio of the between-group
estimate to the within-group estimate.  Since the P-value of the
F-test is less than 0,05, there is a statistically significant
difference between the mean pH from one level of Species to another at
the 95,0% confidence level.  To determine which means are
significantly different from which others, select Multiple Range Tests
from the list of Tabular Options.


Table of Means for pH by Species
with 95,0 percent LSD intervals
--------------------------------------------------------------------------------
                                       Stnd. error
Species          Count         Mean     (pooled s)    Lower limit    Upper limit
--------------------------------------------------------------------------------
C. album             3         6,54      0,0735451        6,42413        6,65587
D.erucoides          3      5,72333      0,0735451        5,60746        5,83921
S.media              3      6,01667      0,0735451        5,90079        6,13254
S.oleraceus          3      6,20667      0,0735451        6,09079        6,32254
T.majus              3      6,00333      0,0735451        5,88746        6,11921
--------------------------------------------------------------------------------
Total               15        6,098


The StatAdvisor
---------------
   This table shows the mean pH for each level of Species.  It also
shows the standard error of each mean, which is a measure of its
sampling variability.  The standard error is formed by dividing the
pooled standard deviation by the square root of the number of
observations at each level.  The table also displays an interval
around each mean.  The intervals currently displayed are based on
Fisher's least significant difference (LSD) procedure.  They are
constructed in such a way that if two means are the same, their
intervals will overlap 95,0% of the time.  You can display the
intervals graphically by selecting Means Plot from the list of
Graphical Options.  In the Multiple Range Tests, these intervals are
used to determine which means are significantly different from which
others.


Multiple Range Tests for pH by Species

--------------------------------------------------------------------------------
Method: 95,0 percent LSD
Species        Count     Mean              Homogeneous Groups
--------------------------------------------------------------------------------
D.erucoides    3         5,72333           X  
T.majus        3         6,00333            X 
S.media        3         6,01667            X 
S.oleraceus    3         6,20667            X 
C. album       3         6,54                X
--------------------------------------------------------------------------------
Contrast                                   Difference           +/-  Limits
--------------------------------------------------------------------------------
C. album - D.erucoides                    *0,816667             0,231746          
C. album - S.media                        *0,523333             0,231746          
C. album - S.oleraceus                    *0,333333             0,231746          
C. album - T.majus                        *0,536667             0,231746          
D.erucoides - S.media                     *-0,293333            0,231746          
D.erucoides - S.oleraceus                 *-0,483333            0,231746          
D.erucoides - T.majus                     *-0,28                0,231746          
S.media - S.oleraceus                      -0,19                0,231746          
S.media - T.majus                          0,0133333            0,231746          
S.oleraceus - T.majus                      0,203333             0,231746          
--------------------------------------------------------------------------------
* denotes a statistically significant difference.


The StatAdvisor
---------------
   This table applies a multiple comparison procedure to determine
which means are significantly different from which others.  The bottom
half of the output shows the estimated difference between each pair of
means.  An asterisk has been placed next to 7 pairs, indicating that
these pairs show statistically significant differences at the 95,0%
confidence level.  At the top of the page, 3 homogenous groups are
identified using columns of X's.  Within each column, the levels
containing X's form a group of means within which there are no
statistically significant differences.  The method currently being
used to discriminate among the means is Fisher's least significant
difference (LSD) procedure.  With this method, there is a 5,0% risk of
calling each pair of means significantly different when the actual
difference equals 0.  


Variance Check

Cochran's C test: 0,476171   P-Value = 0,376468
Bartlett's test: 1,56453   P-Value = 0,443797
Hartley's test: 15,8767
Levene's test: 0,373533   P-Value = 0,822415


The StatAdvisor
---------------
   The four statistics displayed in this table test the null
hypothesis that the standard deviations of pH within each of the 5
levels of Species is the same.  Of particular interest are the three
P-values.  Since the smallest of the P-values is greater than or equal
to 0,05, there is not a statistically significant difference amongst
the standard deviations at the 95,0% confidence level.  


Kruskal-Wallis Test for pH by Species

Species             Sample Size         Average Rank
------------------------------------------------------------
C. album            3                   14,0                
D.erucoides         3                   2,16667             
S.media             3                   6,5                 
S.oleraceus         3                   11,0                
T.majus             3                   6,33333             
------------------------------------------------------------
Test statistic = 12,6535   P-Value = 0,0130989


The StatAdvisor
---------------
   The Kruskal-Wallis test tests the null hypothesis that the medians
of pH within each of the 5 levels of Species are the same.  The data
from all the levels is first combined and ranked from smallest to
largest.  The average rank is then computed for the data at each
level.  Since the P-value is less than 0,05, there is a statistically
significant difference amongst the medians at the 95,0% confidence
level.  To determine which medians are significantly different from
which others, select Box-and-Whisker Plot from the list of Graphical
Options and select the median notch option.


One-Way ANOVA - Total acidity _% cítric acid_ by Species

Analysis Summary

Dependent variable: Total acidity _% cítric acid_
Factor: Species

Number of observations: 15
Number of levels: 5


The StatAdvisor
---------------
   This procedure performs a one-way analysis of variance for Total
acidity _% cítric acid_.  It constructs various tests and graphs to
compare the mean values of Total acidity _% cítric acid_ for the 5
different levels of Species.  The F-test in the ANOVA table will test
whether there are any significant differences amongst the means.  If
there are, the Multiple Range Tests will tell you which means are
significantly different from which others.  If you are worried about
the presence of outliers, choose the Kruskal-Wallis Test which
compares medians instead of means.  The various plots will help you
judge the practical significance of the results, as well as allow you
to look for possible violations of the assumptions underlying the
analysis of variance.  


Summary Statistics for Total acidity _% cítric acid_

Species             Count               Average             Variance            Standard deviation  
----------------------------------------------------------------------------------------------------
C. album            3                   0,123033            0,0000549733        0,0074144           
D.erucoides         3                   0,2888              0,00212569          0,0461052           
S.media             3                   0,1531              0,00018796          0,0137099           
S.oleraceus         3                   0,124233            0,000450893         0,0212342           
T.majus             3                   0,1677              0,00031564          0,0177663           
----------------------------------------------------------------------------------------------------
Total               15                  0,171373            0,00445403          0,0667385           

Species             Minimum             Maximum             Range               Stnd. skewness      
----------------------------------------------------------------------------------------------------
C. album            0,1159              0,1307              0,0148              0,227702            
D.erucoides         0,2431              0,3353              0,0922              0,0551959           
S.media             0,1391              0,1665              0,0274              -0,13899            
S.oleraceus         0,1055              0,1473              0,0418              0,622313            
T.majus             0,1487              0,1839              0,0352              -0,489031           
----------------------------------------------------------------------------------------------------
Total               0,1055              0,3353              0,2298              2,42014             

Species             Stnd. kurtosis      
----------------------------------------------------------------------------------------------------
C. album                                
D.erucoides                             
S.media                                 
S.oleraceus                             
T.majus                                 
----------------------------------------------------------------------------------------------------
Total               1,30802             


The StatAdvisor
---------------
   This table shows various statistics for Total acidity _% cítric
acid_ for each of the 5 levels of Species.  The one-way analysis of
variance is primarily intended to compare the means of the different
levels, listed here under the Average column.  Select Means Plot from
the list of Graphical Options to display the means graphically.  

WARNING: There is more than a 3 to 1 difference between the smallest
standard deviation and the largest.  This may cause problems since the
analysis of variance assumes that the standard deviations at all
levels are equal.  Select Variance Check from the list of Tabular
Options to run a formal statistical test for differences among the
sigmas.  You may want to consider transforming the values of Total
acidity _% cítric acid_ to remove any dependence of the standard
deviation on the mean.  


One-Way ANOVA - Total acidity _% cítric acid_ by Species
ANOVA Table for Total acidity _% cítric acid_ by Species

                            Analysis of Variance
-----------------------------------------------------------------------------
Source             Sum of Squares     Df  Mean Square    F-Ratio      P-Value
-----------------------------------------------------------------------------
Between groups          0,0560861      4    0,0140215      22,36       0,0001
Within groups          0,00627031     10  0,000627031
-----------------------------------------------------------------------------
Total (Corr.)           0,0623564     14


The StatAdvisor
---------------
   The ANOVA table decomposes the variance of Total acidity _% cítric
acid_ into two components: a between-group component and a
within-group component.  The F-ratio, which in this case equals
22,3618, is a ratio of the between-group estimate to the within-group
estimate.  Since the P-value of the F-test is less than 0,05, there is
a statistically significant difference between the mean Total acidity
_% cítric acid_ from one level of Species to another at the 95,0%
confidence level.  To determine which means are significantly
different from which others, select Multiple Range Tests from the list
of Tabular Options.


Table of Means for Total acidity _% cítric acid_ by Species
with 95,0 percent LSD intervals
--------------------------------------------------------------------------------
                                       Stnd. error
Species          Count         Mean     (pooled s)    Lower limit    Upper limit
--------------------------------------------------------------------------------
C. album             3     0,123033      0,0144572       0,100256       0,145811
D.erucoides          3       0,2888      0,0144572       0,266022       0,311578
S.media              3       0,1531      0,0144572       0,130322       0,175878
S.oleraceus          3     0,124233      0,0144572       0,101456       0,147011
T.majus              3       0,1677      0,0144572       0,144922       0,190478
--------------------------------------------------------------------------------
Total               15     0,171373


The StatAdvisor
---------------
   This table shows the mean Total acidity _% cítric acid_ for each
level of Species.  It also shows the standard error of each mean,
which is a measure of its sampling variability.  The standard error is
formed by dividing the pooled standard deviation by the square root of
the number of observations at each level.  The table also displays an
interval around each mean.  The intervals currently displayed are
based on Fisher's least significant difference (LSD) procedure.  They
are constructed in such a way that if two means are the same, their
intervals will overlap 95,0% of the time.  You can display the
intervals graphically by selecting Means Plot from the list of
Graphical Options.  In the Multiple Range Tests, these intervals are
used to determine which means are significantly different from which
others.


Multiple Range Tests for Total acidity _% cítric acid_ by Species

--------------------------------------------------------------------------------
Method: 95,0 percent LSD
Species        Count     Mean              Homogeneous Groups
--------------------------------------------------------------------------------
C. album       3         0,123033          X 
S.oleraceus    3         0,124233          X 
S.media        3         0,1531            X 
T.majus        3         0,1677            X 
D.erucoides    3         0,2888             X
--------------------------------------------------------------------------------
Contrast                                   Difference           +/-  Limits
--------------------------------------------------------------------------------
C. album - D.erucoides                    *-0,165767            0,0455557         
C. album - S.media                         -0,0300667           0,0455557         
C. album - S.oleraceus                     -0,0012              0,0455557         
C. album - T.majus                         -0,0446667           0,0455557         
D.erucoides - S.media                     *0,1357               0,0455557         
D.erucoides - S.oleraceus                 *0,164567             0,0455557         
D.erucoides - T.majus                     *0,1211               0,0455557         
S.media - S.oleraceus                      0,0288667            0,0455557         
S.media - T.majus                          -0,0146              0,0455557         
S.oleraceus - T.majus                      -0,0434667           0,0455557         
--------------------------------------------------------------------------------
* denotes a statistically significant difference.


The StatAdvisor
---------------
   This table applies a multiple comparison procedure to determine
which means are significantly different from which others.  The bottom
half of the output shows the estimated difference between each pair of
means.  An asterisk has been placed next to 4 pairs, indicating that
these pairs show statistically significant differences at the 95,0%
confidence level.  At the top of the page, 2 homogenous groups are
identified using columns of X's.  Within each column, the levels
containing X's form a group of means within which there are no
statistically significant differences.  The method currently being
used to discriminate among the means is Fisher's least significant
difference (LSD) procedure.  With this method, there is a 5,0% risk of
calling each pair of means significantly different when the actual
difference equals 0.  


Variance Check

Cochran's C test: 0,678017   P-Value = 0,0537404
Bartlett's test: 1,98743   P-Value = 0,220755
Hartley's test: 38,6677
Levene's test: 1,33189   P-Value = 0,323633


The StatAdvisor
---------------
   The four statistics displayed in this table test the null
hypothesis that the standard deviations of Total acidity _% cítric
acid_ within each of the 5 levels of Species is the same.  Of
particular interest are the three P-values.  Since the smallest of the
P-values is greater than or equal to 0,05, there is not a
statistically significant difference amongst the standard deviations
at the 95,0% confidence level.  


Kruskal-Wallis Test for Total acidity _% cítric acid_ by Species

Species             Sample Size         Average Rank
------------------------------------------------------------
C. album            3                   3,66667             
D.erucoides         3                   14,0                
S.media             3                   8,33333             
S.oleraceus         3                   3,66667             
T.majus             3                   10,3333             
------------------------------------------------------------
Test statistic = 11,8667   P-Value = 0,0183708


The StatAdvisor
---------------
   The Kruskal-Wallis test tests the null hypothesis that the medians
of Total acidity _% cítric acid_ within each of the 5 levels of
Species are the same.  The data from all the levels is first combined
and ranked from smallest to largest.  The average rank is then
computed for the data at each level.  Since the P-value is less than
0,05, there is a statistically significant difference amongst the
medians at the 95,0% confidence level.  To determine which medians are
significantly different from which others, select Box-and-Whisker Plot
from the list of Graphical Options and select the median notch option.


One-Way ANOVA - TAO _µmol TE·100g_1 fw_ by Species

Analysis Summary

Dependent variable: TAO _µmol TE·100g_1 fw_
Factor: Species

Number of observations: 15
Number of levels: 5


The StatAdvisor
---------------
   This procedure performs a one-way analysis of variance for TAO
_µmol TE·100g_1 fw_.  It constructs various tests and graphs to
compare the mean values of TAO _µmol TE·100g_1 fw_ for the 5 different
levels of Species.  The F-test in the ANOVA table will test whether
there are any significant differences amongst the means.  If there
are, the Multiple Range Tests will tell you which means are
significantly different from which others.  If you are worried about
the presence of outliers, choose the Kruskal-Wallis Test which
compares medians instead of means.  The various plots will help you
judge the practical significance of the results, as well as allow you
to look for possible violations of the assumptions underlying the
analysis of variance.  


Summary Statistics for TAO _µmol TE·100g_1 fw_

Species             Count               Average             Variance            Standard deviation  
----------------------------------------------------------------------------------------------------
C. album            3                   1669,91             37804,0             194,432             
D.erucoides         3                   4227,35             5555,82             74,5373             
S.media             3                   1604,31             57536,4             239,867             
S.oleraceus         3                   1537,12             35057,1             187,235             
T.majus             3                   4874,55             1,75032E6           1323,0              
----------------------------------------------------------------------------------------------------
Total               15                  2782,65             2,54973E6           1596,79             

Species             Minimum             Maximum             Range               Stnd. skewness      
----------------------------------------------------------------------------------------------------
C. album            1507,66             1885,42             377,761             0,806328            
D.erucoides         4155,09             4303,97             148,883             0,18544             
S.media             1348,95             1824,89             475,936             -0,451754           
S.oleraceus         1420,23             1753,08             332,85              1,21226             
T.majus             3429,69             6026,65             2596,96             -0,669636           
----------------------------------------------------------------------------------------------------
Total               1348,95             6026,65             4677,7              1,32228             

Species             Stnd. kurtosis      
----------------------------------------------------------------------------------------------------
C. album                                
D.erucoides                             
S.media                                 
S.oleraceus                             
T.majus                                 
----------------------------------------------------------------------------------------------------
Total               -0,624985           


The StatAdvisor
---------------
   This table shows various statistics for TAO _µmol TE·100g_1 fw_ for
each of the 5 levels of Species.  The one-way analysis of variance is
primarily intended to compare the means of the different levels,
listed here under the Average column.  Select Means Plot from the list
of Graphical Options to display the means graphically.  

WARNING: There is more than a 3 to 1 difference between the smallest
standard deviation and the largest.  This may cause problems since the
analysis of variance assumes that the standard deviations at all
levels are equal.  Select Variance Check from the list of Tabular
Options to run a formal statistical test for differences among the
sigmas.  You may want to consider transforming the values of TAO _µmol
TE·100g_1 fw_ to remove any dependence of the standard deviation on
the mean.  


ANOVA Table for TAO _µmol TE·100g_1 fw_ by Species

                            Analysis of Variance
-----------------------------------------------------------------------------
Source             Sum of Squares     Df  Mean Square    F-Ratio      P-Value
-----------------------------------------------------------------------------
Between groups          3,19236E7      4    7,98091E6      21,16       0,0001
Within groups           3,77256E6     10     377256,0
-----------------------------------------------------------------------------
Total (Corr.)           3,56962E7     14


The StatAdvisor
---------------
   The ANOVA table decomposes the variance of TAO _µmol TE·100g_1 fw_
into two components: a between-group component and a within-group
component.  The F-ratio, which in this case equals 21,1552, is a ratio
of the between-group estimate to the within-group estimate.  Since the
P-value of the F-test is less than 0,05, there is a statistically
significant difference between the mean TAO _µmol TE·100g_1 fw_ from
one level of Species to another at the 95,0% confidence level.  To
determine which means are significantly different from which others,
select Multiple Range Tests from the list of Tabular Options.


Table of Means for TAO _µmol TE·100g_1 fw_ by Species
with 95,0 percent LSD intervals
--------------------------------------------------------------------------------
                                       Stnd. error
Species          Count         Mean     (pooled s)    Lower limit    Upper limit
--------------------------------------------------------------------------------
C. album             3      1669,91        354,615         1111,2        2228,61
D.erucoides          3      4227,35        354,615        3668,64        4786,06
S.media              3      1604,31        354,615         1045,6        2163,02
S.oleraceus          3      1537,12        354,615        978,415        2095,83
T.majus              3      4874,55        354,615        4315,84        5433,26
--------------------------------------------------------------------------------
Total               15      2782,65


The StatAdvisor
---------------
   This table shows the mean TAO _µmol TE·100g_1 fw_ for each level of
Species.  It also shows the standard error of each mean, which is a
measure of its sampling variability.  The standard error is formed by
dividing the pooled standard deviation by the square root of the
number of observations at each level.  The table also displays an
interval around each mean.  The intervals currently displayed are
based on Fisher's least significant difference (LSD) procedure.  They
are constructed in such a way that if two means are the same, their
intervals will overlap 95,0% of the time.  You can display the
intervals graphically by selecting Means Plot from the list of
Graphical Options.  In the Multiple Range Tests, these intervals are
used to determine which means are significantly different from which
others.


Multiple Range Tests for TAO _µmol TE·100g_1 fw_ by Species

--------------------------------------------------------------------------------
Method: 95,0 percent LSD
Species        Count     Mean              Homogeneous Groups
--------------------------------------------------------------------------------
S.oleraceus    3         1537,12           X 
S.media        3         1604,31           X 
C. album       3         1669,91           X 
D.erucoides    3         4227,35            X
T.majus        3         4874,55            X
--------------------------------------------------------------------------------
Contrast                                   Difference           +/-  Limits
--------------------------------------------------------------------------------
C. album - D.erucoides                    *-2557,44             1117,42           
C. album - S.media                         65,5931              1117,42           
C. album - S.oleraceus                     132,781              1117,42           
C. album - T.majus                        *-3204,64             1117,42           
D.erucoides - S.media                     *2623,04              1117,42           
D.erucoides - S.oleraceus                 *2690,23              1117,42           
D.erucoides - T.majus                      -647,2               1117,42           
S.media - S.oleraceus                      67,1882              1117,42           
S.media - T.majus                         *-3270,24             1117,42           
S.oleraceus - T.majus                     *-3337,43             1117,42           
--------------------------------------------------------------------------------
* denotes a statistically significant difference.


The StatAdvisor
---------------
   This table applies a multiple comparison procedure to determine
which means are significantly different from which others.  The bottom
half of the output shows the estimated difference between each pair of
means.  An asterisk has been placed next to 6 pairs, indicating that
these pairs show statistically significant differences at the 95,0%
confidence level.  At the top of the page, 2 homogenous groups are
identified using columns of X's.  Within each column, the levels
containing X's form a group of means within which there are no
statistically significant differences.  The method currently being
used to discriminate among the means is Fisher's least significant
difference (LSD) procedure.  With this method, there is a 5,0% risk of
calling each pair of means significantly different when the actual
difference equals 0.  


Variance Check

Cochran's C test: 0,927925   P-Value = 0,000134929
Bartlett's test: 6,34762   P-Value = 0,00393843
Hartley's test: 315,044
Levene's test: 2,09037   P-Value = 0,15701


The StatAdvisor
---------------
   The four statistics displayed in this table test the null
hypothesis that the standard deviations of TAO _µmol TE·100g_1 fw_
within each of the 5 levels of Species is the same.  Of particular
interest are the three P-values.  Since the smallest of the P-values
is less than 0,05, there is a statistically significant difference
amongst the standard deviations at the 95,0% confidence level.  This
violates one of the important assumptions underlying the analysis of
variance and will invalidate most of the standard statistical tests.  


Kruskal-Wallis Test for TAO _µmol TE·100g_1 fw_ by Species

Species             Sample Size         Average Rank
------------------------------------------------------------
C. album            3                   6,0                 
D.erucoides         3                   12,0                
S.media             3                   5,0                 
S.oleraceus         3                   4,0                 
T.majus             3                   13,0                
------------------------------------------------------------
Test statistic = 10,5   P-Value = 0,032797


The StatAdvisor
---------------
   The Kruskal-Wallis test tests the null hypothesis that the medians
of TAO _µmol TE·100g_1 fw_ within each of the 5 levels of Species are
the same.  The data from all the levels is first combined and ranked
from smallest to largest.  The average rank is then computed for the
data at each level.  Since the P-value is less than 0,05, there is a
statistically significant difference amongst the medians at the 95,0%
confidence level.  To determine which medians are significantly
different from which others, select Box-and-Whisker Plot from the list
of Graphical Options and select the median notch option.


One-Way ANOVA - TPP _mg GAE·100g_1 fw_ by Species

Analysis Summary

Dependent variable: TPP _mg GAE·100g_1 fw_
Factor: Species

Number of observations: 15
Number of levels: 5


The StatAdvisor
---------------
   This procedure performs a one-way analysis of variance for TPP _mg
GAE·100g_1 fw_.  It constructs various tests and graphs to compare the
mean values of TPP _mg GAE·100g_1 fw_ for the 5 different levels of
Species.  The F-test in the ANOVA table will test whether there are
any significant differences amongst the means.  If there are, the
Multiple Range Tests will tell you which means are significantly
different from which others.  If you are worried about the presence of
outliers, choose the Kruskal-Wallis Test which compares medians
instead of means.  The various plots will help you judge the practical
significance of the results, as well as allow you to look for possible
violations of the assumptions underlying the analysis of variance.  


One-Way ANOVA - TPP _mg GAE·100g_1 fw_ by Species

Analysis Summary

Dependent variable: TPP _mg GAE·100g_1 fw_
Factor: Species

Number of observations: 15
Number of levels: 5


The StatAdvisor
---------------
   This procedure performs a one-way analysis of variance for TPP _mg
GAE·100g_1 fw_.  It constructs various tests and graphs to compare the
mean values of TPP _mg GAE·100g_1 fw_ for the 5 different levels of
Species.  The F-test in the ANOVA table will test whether there are
any significant differences amongst the means.  If there are, the
Multiple Range Tests will tell you which means are significantly
different from which others.  If you are worried about the presence of
outliers, choose the Kruskal-Wallis Test which compares medians
instead of means.  The various plots will help you judge the practical
significance of the results, as well as allow you to look for possible
violations of the assumptions underlying the analysis of variance.  


Summary Statistics for TPP _mg GAE·100g_1 fw_

Species             Count               Average             Variance            Standard deviation  
----------------------------------------------------------------------------------------------------
C. album            3                   398,785             27468,1             165,735             
D.erucoides         3                   208,562             1000,77             31,6349             
S.media             3                   398,785             27468,1             165,735             
S.oleraceus         3                   237,612             2453,6              49,5339             
T.majus             3                   378,074             13901,8             117,906             
----------------------------------------------------------------------------------------------------
Total               15                  324,364             17805,6             133,438             

Species             Minimum             Maximum             Range               Stnd. skewness      
----------------------------------------------------------------------------------------------------
C. album            295,67              589,962             294,292             1,21339             
D.erucoides         188,731             245,044             56,3135             1,21085             
S.media             295,67              589,962             294,292             1,21339             
S.oleraceus         180,62              270,302             89,6818             -1,18534            
T.majus             309,042             514,215             205,173             1,22438             
----------------------------------------------------------------------------------------------------
Total               180,62              589,962             409,342             1,95076             

Species             Stnd. kurtosis      
----------------------------------------------------------------------------------------------------
C. album                                
D.erucoides                             
S.media                                 
S.oleraceus                             
T.majus                                 
----------------------------------------------------------------------------------------------------
Total               0,415194            


The StatAdvisor
---------------
   This table shows various statistics for TPP _mg GAE·100g_1 fw_ for
each of the 5 levels of Species.  The one-way analysis of variance is
primarily intended to compare the means of the different levels,
listed here under the Average column.  Select Means Plot from the list
of Graphical Options to display the means graphically.  

WARNING: There is more than a 3 to 1 difference between the smallest
standard deviation and the largest.  This may cause problems since the
analysis of variance assumes that the standard deviations at all
levels are equal.  Select Variance Check from the list of Tabular
Options to run a formal statistical test for differences among the
sigmas.  You may want to consider transforming the values of TPP _mg
GAE·100g_1 fw_ to remove any dependence of the standard deviation on
the mean.  


One-Way ANOVA - TPP _mg GAE·100g_1 fw_ by Species

Analysis Summary

Dependent variable: TPP _mg GAE·100g_1 fw_
Factor: Species

Number of observations: 15
Number of levels: 5


The StatAdvisor
---------------
   This procedure performs a one-way analysis of variance for TPP _mg
GAE·100g_1 fw_.  It constructs various tests and graphs to compare the
mean values of TPP _mg GAE·100g_1 fw_ for the 5 different levels of
Species.  The F-test in the ANOVA table will test whether there are
any significant differences amongst the means.  If there are, the
Multiple Range Tests will tell you which means are significantly
different from which others.  If you are worried about the presence of
outliers, choose the Kruskal-Wallis Test which compares medians
instead of means.  The various plots will help you judge the practical
significance of the results, as well as allow you to look for possible
violations of the assumptions underlying the analysis of variance.  


Summary Statistics for TPP _mg GAE·100g_1 fw_

Species             Count               Average             Variance            Standard deviation  
----------------------------------------------------------------------------------------------------
C. album            3                   398,785             27468,1             165,735             
D.erucoides         3                   208,562             1000,77             31,6349             
S.media             3                   398,785             27468,1             165,735             
S.oleraceus         3                   237,612             2453,6              49,5339             
T.majus             3                   378,074             13901,8             117,906             
----------------------------------------------------------------------------------------------------
Total               15                  324,364             17805,6             133,438             

Species             Minimum             Maximum             Range               Stnd. skewness      
----------------------------------------------------------------------------------------------------
C. album            295,67              589,962             294,292             1,21339             
D.erucoides         188,731             245,044             56,3135             1,21085             
S.media             295,67              589,962             294,292             1,21339             
S.oleraceus         180,62              270,302             89,6818             -1,18534            
T.majus             309,042             514,215             205,173             1,22438             
----------------------------------------------------------------------------------------------------
Total               180,62              589,962             409,342             1,95076             

Species             Stnd. kurtosis      
----------------------------------------------------------------------------------------------------
C. album                                
D.erucoides                             
S.media                                 
S.oleraceus                             
T.majus                                 
----------------------------------------------------------------------------------------------------
Total               0,415194            


The StatAdvisor
---------------
   This table shows various statistics for TPP _mg GAE·100g_1 fw_ for
each of the 5 levels of Species.  The one-way analysis of variance is
primarily intended to compare the means of the different levels,
listed here under the Average column.  Select Means Plot from the list
of Graphical Options to display the means graphically.  

WARNING: There is more than a 3 to 1 difference between the smallest
standard deviation and the largest.  This may cause problems since the
analysis of variance assumes that the standard deviations at all
levels are equal.  Select Variance Check from the list of Tabular
Options to run a formal statistical test for differences among the
sigmas.  You may want to consider transforming the values of TPP _mg
GAE·100g_1 fw_ to remove any dependence of the standard deviation on
the mean.  


ANOVA Table for TPP _mg GAE·100g_1 fw_ by Species

                            Analysis of Variance
-----------------------------------------------------------------------------
Source             Sum of Squares     Df  Mean Square    F-Ratio      P-Value
-----------------------------------------------------------------------------
Between groups           104694,0      4      26173,5       1,81       0,2035
Within groups            144585,0     10      14458,5
-----------------------------------------------------------------------------
Total (Corr.)            249279,0     14


The StatAdvisor
---------------
   The ANOVA table decomposes the variance of TPP _mg GAE·100g_1 fw_
into two components: a between-group component and a within-group
component.  The F-ratio, which in this case equals 1,81026, is a ratio
of the between-group estimate to the within-group estimate.  Since the
P-value of the F-test is greater than or equal to 0,05, there is not a
statistically significant difference between the mean TPP _mg
GAE·100g_1 fw_ from one level of Species to another at the 95,0%
confidence level.


Table of Means for TPP _mg GAE·100g_1 fw_ by Species
with 95,0 percent LSD intervals
--------------------------------------------------------------------------------
                                       Stnd. error
Species          Count         Mean     (pooled s)    Lower limit    Upper limit
--------------------------------------------------------------------------------
C. album             3      398,785        69,4225        289,408        508,163
D.erucoides          3      208,562        69,4225        99,1839        317,939
S.media              3      398,785        69,4225        289,408        508,163
S.oleraceus          3      237,612        69,4225        128,234        346,989
T.majus              3      378,074        69,4225        268,696        487,452
--------------------------------------------------------------------------------
Total               15      324,364


The StatAdvisor
---------------
   This table shows the mean TPP _mg GAE·100g_1 fw_ for each level of
Species.  It also shows the standard error of each mean, which is a
measure of its sampling variability.  The standard error is formed by
dividing the pooled standard deviation by the square root of the
number of observations at each level.  The table also displays an
interval around each mean.  The intervals currently displayed are
based on Fisher's least significant difference (LSD) procedure.  They
are constructed in such a way that if two means are the same, their
intervals will overlap 95,0% of the time.  You can display the
intervals graphically by selecting Means Plot from the list of
Graphical Options.  In the Multiple Range Tests, these intervals are
used to determine which means are significantly different from which
others.


Multiple Range Tests for TPP _mg GAE·100g_1 fw_ by Species

--------------------------------------------------------------------------------
Method: 95,0 percent LSD
Species        Count     Mean              Homogeneous Groups
--------------------------------------------------------------------------------
D.erucoides    3         208,562           X
S.oleraceus    3         237,612           X
T.majus        3         378,074           X
S.media        3         398,785           X
C. album       3         398,785           X
--------------------------------------------------------------------------------
Contrast                                   Difference           +/-  Limits
--------------------------------------------------------------------------------
C. album - D.erucoides                     190,224              218,755           
C. album - S.media                         0,0                  218,755           
C. album - S.oleraceus                     161,174              218,755           
C. album - T.majus                         20,7116              218,755           
D.erucoides - S.media                      -190,224             218,755           
D.erucoides - S.oleraceus                  -29,05               218,755           
D.erucoides - T.majus                      -169,512             218,755           
S.media - S.oleraceus                      161,174              218,755           
S.media - T.majus                          20,7116              218,755           
S.oleraceus - T.majus                      -140,462             218,755           
--------------------------------------------------------------------------------
* denotes a statistically significant difference.


The StatAdvisor
---------------
   This table applies a multiple comparison procedure to determine
which means are significantly different from which others.  The bottom
half of the output shows the estimated difference between each pair of
means.  There are no statistically significant differences between any
pair of means at the 95,0% confidence level.  At the top of the page,
one homogenous group is identified by a column of X's.  Within each
column, the levels containing X's form a group of means within which
there are no statistically significant differences.  The method
currently being used to discriminate among the means is Fisher's least
significant difference (LSD) procedure.  With this method, there is a
5,0% risk of calling each pair of means significantly different when
the actual difference equals 0.  


Variance Check

Cochran's C test: 0,379959   P-Value = 0,739014
Bartlett's test: 1,89648   P-Value = 0,254771
Hartley's test: 27,447
Levene's test: 0,315686   P-Value = 0,861125


The StatAdvisor
---------------
   The four statistics displayed in this table test the null
hypothesis that the standard deviations of TPP _mg GAE·100g_1 fw_
within each of the 5 levels of Species is the same.  Of particular
interest are the three P-values.  Since the smallest of the P-values
is greater than or equal to 0,05, there is not a statistically
significant difference amongst the standard deviations at the 95,0%
confidence level.  


Kruskal-Wallis Test for TPP _mg GAE·100g_1 fw_ by Species

Species             Sample Size         Average Rank
------------------------------------------------------------
C. album            3                   10,8333             
D.erucoides         3                   3,0                 
S.media             3                   10,8333             
S.oleraceus         3                   4,0                 
T.majus             3                   11,3333             
------------------------------------------------------------
Test statistic = 10,2801   P-Value = 0,0359651


The StatAdvisor
---------------
   The Kruskal-Wallis test tests the null hypothesis that the medians
of TPP _mg GAE·100g_1 fw_ within each of the 5 levels of Species are
the same.  The data from all the levels is first combined and ranked
from smallest to largest.  The average rank is then computed for the
data at each level.  Since the P-value is less than 0,05, there is a
statistically significant difference amongst the medians at the 95,0%
confidence level.  To determine which medians are significantly
different from which others, select Box-and-Whisker Plot from the list
of Graphical Options and select the median notch option.


One-Way ANOVA - Chl a _mg·g_1wf_ by Species

Analysis Summary

Dependent variable: Chl a _mg·g_1wf_
Factor: Species

Number of observations: 15
Number of levels: 5


The StatAdvisor
---------------
   This procedure performs a one-way analysis of variance for Chl a
_mg·g_1wf_.  It constructs various tests and graphs to compare the
mean values of Chl a _mg·g_1wf_ for the 5 different levels of Species.
The F-test in the ANOVA table will test whether there are any
significant differences amongst the means.  If there are, the Multiple
Range Tests will tell you which means are significantly different from
which others.  If you are worried about the presence of outliers,
choose the Kruskal-Wallis Test which compares medians instead of
means.  The various plots will help you judge the practical
significance of the results, as well as allow you to look for possible
violations of the assumptions underlying the analysis of variance.  


Summary Statistics for Chl a _mg·g_1wf_

Species             Count               Average             Variance            Standard deviation  
----------------------------------------------------------------------------------------------------
C. album            3                   1,617               0,184053            0,429014            
D.erucoides         3                   0,921667            0,0382663           0,195618            
S.media             3                   1,068               0,000457            0,0213776           
S.oleraceus         3                   2,261               0,021108            0,145286            
T.majus             3                   0,927333            0,159286            0,399107            
----------------------------------------------------------------------------------------------------
Total               15                  1,359               0,345263            0,587591            

Species             Minimum             Maximum             Range               Stnd. skewness      
----------------------------------------------------------------------------------------------------
C. album            1,186               2,044               0,858               -0,0296653          
D.erucoides         0,696               1,043               0,347               -1,21435            
S.media             1,044               1,085               0,041               -0,930211           
S.oleraceus         2,095               2,365               0,27                -1,11061            
T.majus             0,595               1,37                0,775               0,812432            
----------------------------------------------------------------------------------------------------
Total               0,595               2,365               1,77                1,03228             

Species             Stnd. kurtosis      
----------------------------------------------------------------------------------------------------
C. album                                
D.erucoides                             
S.media                                 
S.oleraceus                             
T.majus                                 
----------------------------------------------------------------------------------------------------
Total               -0,741379           


The StatAdvisor
---------------
   This table shows various statistics for Chl a _mg·g_1wf_ for each
of the 5 levels of Species.  The one-way analysis of variance is
primarily intended to compare the means of the different levels,
listed here under the Average column.  Select Means Plot from the list
of Graphical Options to display the means graphically.  

WARNING: There is more than a 3 to 1 difference between the smallest
standard deviation and the largest.  This may cause problems since the
analysis of variance assumes that the standard deviations at all
levels are equal.  Select Variance Check from the list of Tabular
Options to run a formal statistical test for differences among the
sigmas.  You may want to consider transforming the values of Chl a
_mg·g_1wf_ to remove any dependence of the standard deviation on the
mean.  


ANOVA Table for Chl a _mg·g_1wf_ by Species

                            Analysis of Variance
-----------------------------------------------------------------------------
Source             Sum of Squares     Df  Mean Square    F-Ratio      P-Value
-----------------------------------------------------------------------------
Between groups            4,02734      4      1,00683      12,49       0,0007
Within groups            0,806341     10    0,0806341
-----------------------------------------------------------------------------
Total (Corr.)             4,83368     14


The StatAdvisor
---------------
   The ANOVA table decomposes the variance of Chl a _mg·g_1wf_ into
two components: a between-group component and a within-group
component.  The F-ratio, which in this case equals 12,4865, is a ratio
of the between-group estimate to the within-group estimate.  Since the
P-value of the F-test is less than 0,05, there is a statistically
significant difference between the mean Chl a _mg·g_1wf_ from one
level of Species to another at the 95,0% confidence level.  To
determine which means are significantly different from which others,
select Multiple Range Tests from the list of Tabular Options.


Table of Means for Chl a _mg·g_1wf_ by Species
with 95,0 percent LSD intervals
--------------------------------------------------------------------------------
                                       Stnd. error
Species          Count         Mean     (pooled s)    Lower limit    Upper limit
--------------------------------------------------------------------------------
C. album             3        1,617       0,163945         1,3587         1,8753
D.erucoides          3     0,921667       0,163945       0,663365        1,17997
S.media              3        1,068       0,163945       0,809698         1,3263
S.oleraceus          3        2,261       0,163945         2,0027         2,5193
T.majus              3     0,927333       0,163945       0,669032        1,18563
--------------------------------------------------------------------------------
Total               15        1,359


The StatAdvisor
---------------
   This table shows the mean Chl a _mg·g_1wf_ for each level of
Species.  It also shows the standard error of each mean, which is a
measure of its sampling variability.  The standard error is formed by
dividing the pooled standard deviation by the square root of the
number of observations at each level.  The table also displays an
interval around each mean.  The intervals currently displayed are
based on Fisher's least significant difference (LSD) procedure.  They
are constructed in such a way that if two means are the same, their
intervals will overlap 95,0% of the time.  You can display the
intervals graphically by selecting Means Plot from the list of
Graphical Options.  In the Multiple Range Tests, these intervals are
used to determine which means are significantly different from which
others.


Multiple Range Tests for Chl a _mg·g_1wf_ by Species

--------------------------------------------------------------------------------
Method: 95,0 percent LSD
Species        Count     Mean              Homogeneous Groups
--------------------------------------------------------------------------------
D.erucoides    3         0,921667          X  
T.majus        3         0,927333          X  
S.media        3         1,068             X  
C. album       3         1,617              X 
S.oleraceus    3         2,261               X
--------------------------------------------------------------------------------
Contrast                                   Difference           +/-  Limits
--------------------------------------------------------------------------------
C. album - D.erucoides                    *0,695333             0,516603          
C. album - S.media                        *0,549                0,516603          
C. album - S.oleraceus                    *-0,644               0,516603          
C. album - T.majus                        *0,689667             0,516603          
D.erucoides - S.media                      -0,146333            0,516603          
D.erucoides - S.oleraceus                 *-1,33933             0,516603          
D.erucoides - T.majus                      -0,00566667          0,516603          
S.media - S.oleraceus                     *-1,193               0,516603          
S.media - T.majus                          0,140667             0,516603          
S.oleraceus - T.majus                     *1,33367              0,516603          
--------------------------------------------------------------------------------
* denotes a statistically significant difference.


The StatAdvisor
---------------
   This table applies a multiple comparison procedure to determine
which means are significantly different from which others.  The bottom
half of the output shows the estimated difference between each pair of
means.  An asterisk has been placed next to 7 pairs, indicating that
these pairs show statistically significant differences at the 95,0%
confidence level.  At the top of the page, 3 homogenous groups are
identified using columns of X's.  Within each column, the levels
containing X's form a group of means within which there are no
statistically significant differences.  The method currently being
used to discriminate among the means is Fisher's least significant
difference (LSD) procedure.  With this method, there is a 5,0% risk of
calling each pair of means significantly different when the actual
difference equals 0.  


Variance Check

Cochran's C test: 0,456514   P-Value = 0,436238
Bartlett's test: 3,15979   P-Value = 0,047979
Hartley's test: 402,742
Levene's test: 1,06651   P-Value = 0,422264


The StatAdvisor
---------------
   The four statistics displayed in this table test the null
hypothesis that the standard deviations of Chl a _mg·g_1wf_ within
each of the 5 levels of Species is the same.  Of particular interest
are the three P-values.  Since the smallest of the P-values is less
than 0,05, there is a statistically significant difference amongst the
standard deviations at the 95,0% confidence level.  This violates one
of the important assumptions underlying the analysis of variance and
will invalidate most of the standard statistical tests.  


Kruskal-Wallis Test for Chl a _mg·g_1wf_ by Species

Species             Sample Size         Average Rank
------------------------------------------------------------
C. album            3                   10,6667             
D.erucoides         3                   3,66667             
S.media             3                   7,0                 
S.oleraceus         3                   14,0                
T.majus             3                   4,66667             
------------------------------------------------------------
Test statistic = 11,1   P-Value = 0,0254628


The StatAdvisor
---------------
   The Kruskal-Wallis test tests the null hypothesis that the medians
of Chl a _mg·g_1wf_ within each of the 5 levels of Species are the
same.  The data from all the levels is first combined and ranked from
smallest to largest.  The average rank is then computed for the data
at each level.  Since the P-value is less than 0,05, there is a
statistically significant difference amongst the medians at the 95,0%
confidence level.  To determine which medians are significantly
different from which others, select Box-and-Whisker Plot from the list
of Graphical Options and select the median notch option.


One-Way ANOVA - Chl b _mg·g_1wf_ by Species

Analysis Summary

Dependent variable: Chl b _mg·g_1wf_
Factor: Species

Number of observations: 15
Number of levels: 5


The StatAdvisor
---------------
   This procedure performs a one-way analysis of variance for Chl b
_mg·g_1wf_.  It constructs various tests and graphs to compare the
mean values of Chl b _mg·g_1wf_ for the 5 different levels of Species.
The F-test in the ANOVA table will test whether there are any
significant differences amongst the means.  If there are, the Multiple
Range Tests will tell you which means are significantly different from
which others.  If you are worried about the presence of outliers,
choose the Kruskal-Wallis Test which compares medians instead of
means.  The various plots will help you judge the practical
significance of the results, as well as allow you to look for possible
violations of the assumptions underlying the analysis of variance.  


Summary Statistics for Chl b _mg·g_1wf_

Species             Count               Average             Variance            Standard deviation  
----------------------------------------------------------------------------------------------------
C. album            3                   0,467333            0,0116243           0,107816            
D.erucoides         3                   0,325667            0,00296233          0,0544273           
S.media             3                   0,461333            0,00130433          0,0361156           
S.oleraceus         3                   0,832333            0,00258233          0,0508167           
T.majus             3                   0,808333            0,0836123           0,289158            
----------------------------------------------------------------------------------------------------
Total               15                  0,579               0,0589974           0,242894            

Species             Minimum             Maximum             Range               Stnd. skewness      
----------------------------------------------------------------------------------------------------
C. album            0,349               0,56                0,211               -0,714571           
D.erucoides         0,264               0,367               0,103               -1,02284            
S.media             0,427               0,499               0,072               0,291183            
S.oleraceus         0,774               0,867               0,093               -1,1605             
T.majus             0,565               1,128               0,563               0,781457            
----------------------------------------------------------------------------------------------------
Total               0,264               1,128               0,864               1,36011             

Species             Stnd. kurtosis      
----------------------------------------------------------------------------------------------------
C. album                                
D.erucoides                             
S.media                                 
S.oleraceus                             
T.majus                                 
----------------------------------------------------------------------------------------------------
Total               0,116581            


The StatAdvisor
---------------
   This table shows various statistics for Chl b _mg·g_1wf_ for each
of the 5 levels of Species.  The one-way analysis of variance is
primarily intended to compare the means of the different levels,
listed here under the Average column.  Select Means Plot from the list
of Graphical Options to display the means graphically.  

WARNING: There is more than a 3 to 1 difference between the smallest
standard deviation and the largest.  This may cause problems since the
analysis of variance assumes that the standard deviations at all
levels are equal.  Select Variance Check from the list of Tabular
Options to run a formal statistical test for differences among the
sigmas.  You may want to consider transforming the values of Chl b
_mg·g_1wf_ to remove any dependence of the standard deviation on the
mean.  


ANOVA Table for Chl b _mg·g_1wf_ by Species

                            Analysis of Variance
-----------------------------------------------------------------------------
Source             Sum of Squares     Df  Mean Square    F-Ratio      P-Value
-----------------------------------------------------------------------------
Between groups           0,621793      4     0,155448       7,61       0,0044
Within groups            0,204171     10    0,0204171
-----------------------------------------------------------------------------
Total (Corr.)            0,825964     14


The StatAdvisor
---------------
   The ANOVA table decomposes the variance of Chl b _mg·g_1wf_ into
two components: a between-group component and a within-group
component.  The F-ratio, which in this case equals 7,61361, is a ratio
of the between-group estimate to the within-group estimate.  Since the
P-value of the F-test is less than 0,05, there is a statistically
significant difference between the mean Chl b _mg·g_1wf_ from one
level of Species to another at the 95,0% confidence level.  To
determine which means are significantly different from which others,
select Multiple Range Tests from the list of Tabular Options.


Table of Means for Chl b _mg·g_1wf_ by Species
with 95,0 percent LSD intervals
--------------------------------------------------------------------------------
                                       Stnd. error
Species          Count         Mean     (pooled s)    Lower limit    Upper limit
--------------------------------------------------------------------------------
C. album             3     0,467333      0,0824967       0,337357        0,59731
D.erucoides          3     0,325667      0,0824967        0,19569       0,455643
S.media              3     0,461333      0,0824967       0,331357        0,59131
S.oleraceus          3     0,832333      0,0824967       0,702357        0,96231
T.majus              3     0,808333      0,0824967       0,678357        0,93831
--------------------------------------------------------------------------------
Total               15        0,579


The StatAdvisor
---------------
   This table shows the mean Chl b _mg·g_1wf_ for each level of
Species.  It also shows the standard error of each mean, which is a
measure of its sampling variability.  The standard error is formed by
dividing the pooled standard deviation by the square root of the
number of observations at each level.  The table also displays an
interval around each mean.  The intervals currently displayed are
based on Fisher's least significant difference (LSD) procedure.  They
are constructed in such a way that if two means are the same, their
intervals will overlap 95,0% of the time.  You can display the
intervals graphically by selecting Means Plot from the list of
Graphical Options.  In the Multiple Range Tests, these intervals are
used to determine which means are significantly different from which
others.


Multiple Range Tests for Chl b _mg·g_1wf_ by Species

--------------------------------------------------------------------------------
Method: 95,0 percent LSD
Species        Count     Mean              Homogeneous Groups
--------------------------------------------------------------------------------
D.erucoides    3         0,325667          X 
S.media        3         0,461333          X 
C. album       3         0,467333          X 
T.majus        3         0,808333           X
S.oleraceus    3         0,832333           X
--------------------------------------------------------------------------------
Contrast                                   Difference           +/-  Limits
--------------------------------------------------------------------------------
C. album - D.erucoides                     0,141667             0,259953          
C. album - S.media                         0,006                0,259953          
C. album - S.oleraceus                    *-0,365               0,259953          
C. album - T.majus                        *-0,341               0,259953          
D.erucoides - S.media                      -0,135667            0,259953          
D.erucoides - S.oleraceus                 *-0,506667            0,259953          
D.erucoides - T.majus                     *-0,482667            0,259953          
S.media - S.oleraceus                     *-0,371               0,259953          
S.media - T.majus                         *-0,347               0,259953          
S.oleraceus - T.majus                      0,024                0,259953          
--------------------------------------------------------------------------------
* denotes a statistically significant difference.


The StatAdvisor
---------------
   This table applies a multiple comparison procedure to determine
which means are significantly different from which others.  The bottom
half of the output shows the estimated difference between each pair of
means.  An asterisk has been placed next to 6 pairs, indicating that
these pairs show statistically significant differences at the 95,0%
confidence level.  At the top of the page, 2 homogenous groups are
identified using columns of X's.  Within each column, the levels
containing X's form a group of means within which there are no
statistically significant differences.  The method currently being
used to discriminate among the means is Fisher's least significant
difference (LSD) procedure.  With this method, there is a 5,0% risk of
calling each pair of means significantly different when the actual
difference equals 0.  


Variance Check

Cochran's C test: 0,819041   P-Value = 0,00536157
Bartlett's test: 3,25582   P-Value = 0,0432642
Hartley's test: 64,1035
Levene's test: 1,43611   P-Value = 0,291926


The StatAdvisor
---------------
   The four statistics displayed in this table test the null
hypothesis that the standard deviations of Chl b _mg·g_1wf_ within
each of the 5 levels of Species is the same.  Of particular interest
are the three P-values.  Since the smallest of the P-values is less
than 0,05, there is a statistically significant difference amongst the
standard deviations at the 95,0% confidence level.  This violates one
of the important assumptions underlying the analysis of variance and
will invalidate most of the standard statistical tests.  


Kruskal-Wallis Test for Chl b _mg·g_1wf_ by Species

Species             Sample Size         Average Rank
------------------------------------------------------------
C. album            3                   6,33333             
D.erucoides         3                   2,33333             
S.media             3                   6,33333             
S.oleraceus         3                   13,0                
T.majus             3                   12,0                
------------------------------------------------------------
Test statistic = 11,8   P-Value = 0,0189022


The StatAdvisor
---------------
   The Kruskal-Wallis test tests the null hypothesis that the medians
of Chl b _mg·g_1wf_ within each of the 5 levels of Species are the
same.  The data from all the levels is first combined and ranked from
smallest to largest.  The average rank is then computed for the data
at each level.  Since the P-value is less than 0,05, there is a
statistically significant difference amongst the medians at the 95,0%
confidence level.  To determine which medians are significantly
different from which others, select Box-and-Whisker Plot from the list
of Graphical Options and select the median notch option.


One-Way ANOVA - Total Chl _mg·g_1wf_ by Species

Analysis Summary

Dependent variable: Total Chl _mg·g_1wf_
Factor: Species

Number of observations: 15
Number of levels: 5


The StatAdvisor
---------------
   This procedure performs a one-way analysis of variance for Total
Chl _mg·g_1wf_.  It constructs various tests and graphs to compare the
mean values of Total Chl _mg·g_1wf_ for the 5 different levels of
Species.  The F-test in the ANOVA table will test whether there are
any significant differences amongst the means.  If there are, the
Multiple Range Tests will tell you which means are significantly
different from which others.  If you are worried about the presence of
outliers, choose the Kruskal-Wallis Test which compares medians
instead of means.  The various plots will help you judge the practical
significance of the results, as well as allow you to look for possible
violations of the assumptions underlying the analysis of variance.  


Summary Statistics for Total Chl _mg·g_1wf_

Species             Count               Average             Variance            Standard deviation  
----------------------------------------------------------------------------------------------------
C. album            3                   2,084               0,2869              0,53563             
D.erucoides         3                   1,24733             0,0619243           0,248846            
S.media             3                   1,529               0,001539            0,0392301           
S.oleraceus         3                   3,09233             0,0379843           0,194896            
T.majus             3                   1,73467             0,473694            0,688255            
----------------------------------------------------------------------------------------------------
Total               15                  1,93747             0,560173            0,748447            

Species             Minimum             Maximum             Range               Stnd. skewness      
----------------------------------------------------------------------------------------------------
C. album            1,534               2,604               1,07                -0,17766            
D.erucoides         0,96                1,393               0,433               -1,22439            
S.media             1,502               1,574               0,072               1,15263             
S.oleraceus         2,868               3,22                0,352               -1,18998            
T.majus             1,159               2,497               1,338               0,799527            
----------------------------------------------------------------------------------------------------
Total               0,96                3,22                2,26                1,02182             

Species             Stnd. kurtosis      
----------------------------------------------------------------------------------------------------
C. album                                
D.erucoides                             
S.media                                 
S.oleraceus                             
T.majus                                 
----------------------------------------------------------------------------------------------------
Total               -0,820446           


The StatAdvisor
---------------
   This table shows various statistics for Total Chl _mg·g_1wf_ for
each of the 5 levels of Species.  The one-way analysis of variance is
primarily intended to compare the means of the different levels,
listed here under the Average column.  Select Means Plot from the list
of Graphical Options to display the means graphically.  

WARNING: There is more than a 3 to 1 difference between the smallest
standard deviation and the largest.  This may cause problems since the
analysis of variance assumes that the standard deviations at all
levels are equal.  Select Variance Check from the list of Tabular
Options to run a formal statistical test for differences among the
sigmas.  You may want to consider transforming the values of Total Chl
_mg·g_1wf_ to remove any dependence of the standard deviation on the
mean.  


ANOVA Table for Total Chl _mg·g_1wf_ by Species

                            Analysis of Variance
-----------------------------------------------------------------------------
Source             Sum of Squares     Df  Mean Square    F-Ratio      P-Value
-----------------------------------------------------------------------------
Between groups            6,11834      4      1,52958       8,87       0,0025
Within groups             1,72408     10     0,172408
-----------------------------------------------------------------------------
Total (Corr.)             7,84242     14


The StatAdvisor
---------------
   The ANOVA table decomposes the variance of Total Chl _mg·g_1wf_
into two components: a between-group component and a within-group
component.  The F-ratio, which in this case equals 8,87187, is a ratio
of the between-group estimate to the within-group estimate.  Since the
P-value of the F-test is less than 0,05, there is a statistically
significant difference between the mean Total Chl _mg·g_1wf_ from one
level of Species to another at the 95,0% confidence level.  To
determine which means are significantly different from which others,
select Multiple Range Tests from the list of Tabular Options.


Table of Means for Total Chl _mg·g_1wf_ by Species
with 95,0 percent LSD intervals
--------------------------------------------------------------------------------
                                       Stnd. error
Species          Count         Mean     (pooled s)    Lower limit    Upper limit
--------------------------------------------------------------------------------
C. album             3        2,084       0,239728         1,7063         2,4617
D.erucoides          3      1,24733       0,239728       0,869633        1,62503
S.media              3        1,529       0,239728         1,1513         1,9067
S.oleraceus          3      3,09233       0,239728        2,71463        3,47003
T.majus              3      1,73467       0,239728        1,35697        2,11237
--------------------------------------------------------------------------------
Total               15      1,93747


The StatAdvisor
---------------
   This table shows the mean Total Chl _mg·g_1wf_ for each level of
Species.  It also shows the standard error of each mean, which is a
measure of its sampling variability.  The standard error is formed by
dividing the pooled standard deviation by the square root of the
number of observations at each level.  The table also displays an
interval around each mean.  The intervals currently displayed are
based on Fisher's least significant difference (LSD) procedure.  They
are constructed in such a way that if two means are the same, their
intervals will overlap 95,0% of the time.  You can display the
intervals graphically by selecting Means Plot from the list of
Graphical Options.  In the Multiple Range Tests, these intervals are
used to determine which means are significantly different from which
others.


Multiple Range Tests for Total Chl _mg·g_1wf_ by Species

--------------------------------------------------------------------------------
Method: 95,0 percent LSD
Species        Count     Mean              Homogeneous Groups
--------------------------------------------------------------------------------
D.erucoides    3         1,24733           X  
S.media        3         1,529             XX 
T.majus        3         1,73467           XX 
C. album       3         2,084              X 
S.oleraceus    3         3,09233             X
--------------------------------------------------------------------------------
Contrast                                   Difference           +/-  Limits
--------------------------------------------------------------------------------
C. album - D.erucoides                    *0,836667             0,7554            
C. album - S.media                         0,555                0,7554            
C. album - S.oleraceus                    *-1,00833             0,7554            
C. album - T.majus                         0,349333             0,7554            
D.erucoides - S.media                      -0,281667            0,7554            
D.erucoides - S.oleraceus                 *-1,845               0,7554            
D.erucoides - T.majus                      -0,487333            0,7554            
S.media - S.oleraceus                     *-1,56333             0,7554            
S.media - T.majus                          -0,205667            0,7554            
S.oleraceus - T.majus                     *1,35767              0,7554            
--------------------------------------------------------------------------------
* denotes a statistically significant difference.


The StatAdvisor
---------------
   This table applies a multiple comparison procedure to determine
which means are significantly different from which others.  The bottom
half of the output shows the estimated difference between each pair of
means.  An asterisk has been placed next to 5 pairs, indicating that
these pairs show statistically significant differences at the 95,0%
confidence level.  At the top of the page, 3 homogenous groups are
identified using columns of X's.  Within each column, the levels
containing X's form a group of means within which there are no
statistically significant differences.  The method currently being
used to discriminate among the means is Fisher's least significant
difference (LSD) procedure.  With this method, there is a 5,0% risk of
calling each pair of means significantly different when the actual
difference equals 0.  


Variance Check

Cochran's C test: 0,549503   P-Value = 0,205939
Bartlett's test: 3,14899   P-Value = 0,0485485
Hartley's test: 307,794
Levene's test: 1,11527   P-Value = 0,402019


The StatAdvisor
---------------
   The four statistics displayed in this table test the null
hypothesis that the standard deviations of Total Chl _mg·g_1wf_ within
each of the 5 levels of Species is the same.  Of particular interest
are the three P-values.  Since the smallest of the P-values is less
than 0,05, there is a statistically significant difference amongst the
standard deviations at the 95,0% confidence level.  This violates one
of the important assumptions underlying the analysis of variance and
will invalidate most of the standard statistical tests.  


Kruskal-Wallis Test for Total Chl _mg·g_1wf_ by Species

Species             Sample Size         Average Rank
------------------------------------------------------------
C. album            3                   9,66667             
D.erucoides         3                   2,66667             
S.media             3                   6,66667             
S.oleraceus         3                   14,0                
T.majus             3                   7,0                 
------------------------------------------------------------
Test statistic = 10,5   P-Value = 0,032797


The StatAdvisor
---------------
   The Kruskal-Wallis test tests the null hypothesis that the medians
of Total Chl _mg·g_1wf_ within each of the 5 levels of Species are the
same.  The data from all the levels is first combined and ranked from
smallest to largest.  The average rank is then computed for the data
at each level.  Since the P-value is less than 0,05, there is a
statistically significant difference amongst the medians at the 95,0%
confidence level.  To determine which medians are significantly
different from which others, select Box-and-Whisker Plot from the list
of Graphical Options and select the median notch option.
