# Supplementary material for: Five undervalued edible species inherent to autumn-winter season: nutritional composition, bioactive constituents and volatiles profile
Source: PeerJ. 2021 Nov 23;9:e12488. doi: 10.7717/peerj.12488 (PMC8621719; doi:10.7717/peerj.12488)
Supplement: Supplemental Information 2 [file peerj-09-12488-s002.rtf]

Principal Components Analysis

Analysis Summary

Data variables: 
     HM (89,841)
     AS (1,859)
     CP (1,901)
     FT (0,558)
     CF (4,390)
     CH (1,460)
     Ca (104,851)
     Mg (63,298)
     K (533,206)
     P (45,780)
     Na (16,360)
     Fe (0,540)
     Cu (0,049)
     Zn (0,630
     NO3- (60,932)
     pH (5,78)
     TA (0,1839)

Data input: observations
Number of complete cases: 14
Missing value treatment: listwise
Standardized: yes

Number of components extracted: 4

         Principal Components Analysis
-----------------------------------------------
Component               Percent of   Cumulative
 Number     Eigenvalue   Variance    Percentage
    1       9,26332       54,490       54,490
    2       3,63202       21,365       75,855
    3       1,55423        9,143       84,997
    4       1,21532        7,149       92,146
    5       0,514812       3,028       95,175
    6       0,421055       2,477       97,651
    7       0,141031       0,830       98,481
    8       0,0934958      0,550       99,031
    9       0,0733103      0,431       99,462
   10       0,0508605      0,299       99,761
   11       0,024635       0,145       99,906
   12       0,0146093      0,086       99,992
   13       0,00130455     0,008      100,000
   14       4,5363E-16     0,000      100,000
   15       2,54721E-16    0,000      100,000
   16       0,0            0,000      100,000
   17       0,0            0,000      100,000
-----------------------------------------------


The StatAdvisor
---------------
   This procedure performs a principal components analysis.  The
purpose of the analysis is to obtain a small number of linear
combinations of the 17 variables which account for most of the
variability in the data.  In this case, 4 components have been
extracted, since 0 components had eigenvalues greater than or equal to
1,0.  Together they account for 92,1464% of the variability in the
original data.


Table of Component Weights

               Component    Component    Component    Component    
                   1            2            3            4
               ------------ ------------ ------------ ------------ 
HM             -0,318908     0,111865    -0,0304418    -0,0126701  
AS              0,320338    -0,0510222   -0,130448    -0,0260653   
CP              0,158796    -0,289464     0,419479     0,260376    
FT             -0,177405     0,298891     0,178787     -0,479542   
CF              0,222935    -0,0195477    0,560273     0,0334755   
CH              0,262472    -0,0847786    -0,452166    -0,063473   
Ca              0,313944     0,0511465    0,0665361    -0,14927     
Mg              0,312762    -0,0674818    -0,0932858   -0,161385    
K               0,261059     0,277941    -0,0727835   -0,180044    
P               0,31942     -0,0101001    0,000400447  0,0177115    
Na             -0,133341     0,314004    -0,0664976   0,576976     
Fe              0,241712     0,071914    -0,404367    0,263672     
Cu              0,225493     0,00834135     0,168755     0,314231     
Zn              0,170537     0,344718     0,134139     0,239496     
NO3-            -0,11751     0,467127     -0,0272307   0,131531     
pH              0,254327     0,272818      0,138122     -0,181552    
TA             -0,138614     -0,45206      -0,0558331   0,0800763    


The StatAdvisor
---------------
   This table shows the equations of the principal components.  For
example, the first principal component has the equation 

-0,318908*HM + 0,320338*AS + 0,158796*CP - 0,177405*FT + 0,222935*CF + 0,262472*CH +
0,313944*Ca + 0,312762*Mg + 0,261059*K + 0,31942*P - 0,133341*Na + 0,241712*Fe + 0,225493*Cu
+ 0,170537*Zn - 0,11751*NO3- + 0,254327*pH - 0,138614*TA

where the values of the variables in the equation are standardized by
subtracting their means and dividing by their standard deviations.


Table of Principal Components

                         Component    Component    Component    Component    
Row     Label                1            2            3            4
------  ---------------  ------------ ------------ ------------ ------------ 
1       1                -1,16896      0,148208    2,38089      -0,218781    
2       1                -1,43915      0,597616    2,18012      -1,29516     
3       2                -3,58201      1,38376     -0,835963    -2,42949     
4       2                -2,23414      1,11188     -1,7479      -0,339116    
5       2                -0,611642     1,41922     -2,10001     0,724466     
6       3                -0,410622     1,96842     0,743789     1,56681      
7       3                -0,327702     2,31549     0,375188      1,5099       
8       3                -1,47585      1,19599    -0,00393528    0,50744      
9       4                -4,12268     -0,508352     -0,323249    -1,04885     
10      4                 5,97291     -0,502015     -0,648728    -0,511311    
11      4                 5,94251      0,359078    0,466416     0,039949     
12      5                -2,28282     -3,64011      0,00973911   0,22044      
13      5                -1,23973     -2,99151      -0,420252    0,239065     
14      5                -1,26548     -2,85769      -0,0761175   1,03464      


The StatAdvisor
---------------
   This table shows the values of the principal components for each
row of your data file.  Select Component Weights from the list of
Tabular Options to obtain the equations for each component.  Select 2D
Scatterplot or 3D Scatterplot from the list of Graphical Options to
plot this data.  You may save the components by pressing the fourth
button from the left on the analysis toolbar.


This plot shows the values of three principal components.  There is one point for each row in the data file.  You can list the component values by selecting Data Table from the list of Tabular Options.


Factorability Tests
Kaiser-Meyer-Olkin Measure of Sampling Adequacy
KMO = 1,#QNAN

Bartlett's Test of Sphericity
Chi-Square = 1,#QNAN
D.F. = 136
P-Value = 1,0

The StatAdvisor
The factorability tests provide indications of whether or not it is likely to be worthwhile attempting to extract factors from a set of variables.  The KMO statistic provides an indication of how much common variance is present.  For factorization to be worthwhile, KMO should normally be at least 0.6.  Since KMO = 1,#QNAN, factorization is not likely to provide much interesting information about any underlying factors.  

Bartlett's test for sphericity tests the hypothesis that the correlation matrix amongst the variables is an identity matrix, indicating that they share no common variance.  Since the P-value is < 0,05, that hypothesis is rejected.  Note: Bartlett's test is very sensitive and is usually ignored unless the number of samples per variable is no more than 5.  In this case, the number of samples per variable equals 0,823529


This plot shows the weights for selected principal components.  There is one point on the plot for each variable.  Reference lines have also been drawn at 0 in each dimension.  A weight close to 0 indicates little contribution of the variable to that component.


This plot shows the weights for selected principal components.  There is one point on the plot for each variable.  A weight close to 0 indicates little contribution of the variable to that component.
