# Supplementary material for: Five undervalued edible species inherent to autumn-winter season: nutritional composition, bioactive constituents and volatiles profile
Source: PeerJ. 2021 Nov 23;9:e12488. doi: 10.7717/peerj.12488 (PMC8621719; doi:10.7717/peerj.12488)
Supplement: Supplemental Information 3 [file peerj-09-12488-s003.rtf]

Principal Components Analysis
Data variables: 
     TAO
     TPP
     Chl a
     Chl b
     Total Chl

Data input: observations
Number of complete cases: 15
Missing value treatment: listwise
Standardized: yes

Number of components extracted: 2

Principal Components Analysis
Component		Percent of	Cumulative	
 Number	Eigenvalue	Variance	Percentage	
1	2,7614	55,228	55,228	
2	1,30547	26,109	81,337	
3	0,82919	16,584	97,921	
4	0,10394	2,079	100,000	
5	1,51036E-7	0,000	100,000	

The StatAdvisor
This procedure performs a principal components analysis.  The purpose of the analysis is to obtain a small number of linear combinations of the 5 variables which account for most of the variability in the data.  In this case, 2 components have been extracted, since 2 components had eigenvalues greater than or equal to 1,0.  Together they account for 81,3374% of the variability in the original data.


This plot shows the eigenvalues for each of the 5 principal components.  The eigenvalues are proportional to the percent of the variability in the data attributable to the components.  You can also see a horizontal line at 1,0, which was the value used to decide on extracting 2 components.


Table of Component Weights
	Component	Component	
	1	2	
TAO	-0,232742	0,773813	
TPP	-0,242603	-0,487416	
Chl a	0,579614	-0,164382	
Chl b	0,440146	0,369502	
Total Chl	0,597741	-0,009212	

The StatAdvisor
This table shows the equations of the principal components.  For example, the first principal component has the equation 

 - 0,232742*TAO - 0,242603*TPP + 0,579614*Chl a + 0,440146*Chl b + 0,597741*Total Chl

where the values of the variables in the equation are standardized by subtracting their means and dividing by their standard deviations.


This plot shows the values of two principal components.  There is one point for each row in the data file.  You can list the component values by selecting Data Table from the list of Tabular Options.


Table of Principal Components
		Component	Component	
Row	Label	1	2	
1	T.majus	-1,84019	-0,177903	
2	T.majus	-0,888161	1,60076	
3	T.majus	1,00408	2,44621	
4	S.media	-0,454253	-0,627745	
5	S.media	-0,694833	-0,653676	
6	S.media	-1,22105	-1,52489	
7	S.oleraceus	2,13205	-0,344185	
8	S.oleraceus	2,88373	0,179156	
9	S.oleraceus	2,81545	-0,338597	
10	C. album	0,481596	-0,719355	
11	C. album	1,36835	-0,743973	
12	C. album	-1,26177	-1,7015	
13	D.erucoides	-1,1166	0,959178	
14	D.erucoides	-2,06129	0,673143	
15	D.erucoides	-1,1471	0,973379	

The StatAdvisor
This table shows the values of the principal components for each row of your data file.  Select Component Weights from the list of Tabular Options to obtain the equations for each component.  Select 2D Scatterplot or 3D Scatterplot from the list of Graphical Options to plot this data.  You may save the components by pressing the fourth button from the left on the analysis toolbar.


This plot cannot be created since less than 3 components were extracted.  You can force other components to be extracted by pressing the alternate mouse button, selecting Analysis Options, and specifying the number of components to extract.


Factorability Tests
Kaiser-Meyer-Olkin Measure of Sampling Adequacy
KMO = 0,421277

Bartlett's Test of Sphericity
Chi-Square = 194,059
D.F. = 10
P-Value = 0,0

The StatAdvisor
The factorability tests provide indications of whether or not it is likely to be worthwhile attempting to extract factors from a set of variables.  The KMO statistic provides an indication of how much common variance is present.  For factorization to be worthwhile, KMO should normally be at least 0.6.  Since KMO = 0,421277, factorization is not likely to provide much interesting information about any underlying factors.  

Bartlett's test for sphericity tests the hypothesis that the correlation matrix amongst the variables is an identity matrix, indicating that they share no common variance.  Since the P-value is < 0,05, that hypothesis is rejected.  Note: Bartlett's test is very sensitive and is usually ignored unless the number of samples per variable is no more than 5.  In this case, the number of samples per variable equals 3,0.


This plot shows the weights for selected principal components.  There is one point on the plot for each variable.  Reference lines have also been drawn at 0 in each dimension.  A weight close to 0 indicates little contribution of the variable to that component.


This plot cannot be created since less than 3 components were extracted.  You can force other components to be extracted by pressing the alternate mouse button, selecting Analysis Options, and specifying the number of components to extract.
